# Pendular mechanism determinants and elastic energy usage during walking of obese and non‐obese children

**DOI:** 10.1113/EP091408

**Published:** 2023-09-18

**Authors:** Leonardo Alexandre Peyré‐Tartaruga, Henrique Bianchi Oliveira, Arthur H. Dewolf, Cosme Franklim Buzzachera, Flávia Gomes Martinez, André Ivaniski‐Mello

**Affiliations:** ^1^ LaBiodin Biodynamics Laboratory, School of Physical Education, Physiotherapy and Dance Universidade Federal do Rio Grande do Sul Porto Alegre Brazil; ^2^ Department of Public Health, Experimental Medicine and Forensic Sciences University of Pavia Pavia Italy; ^3^ Laboratory of Biomechanics and Physiology of Locomotion, Institute of NeuroScience Université Catholique de Louvain Louvain‐la‐Neuve Belgium

**Keywords:** biomechanics, energy, gait, locomotion, obesity, recovery

## Abstract

The mechanical and metabolic responses of walking by obese children are not yet well understood. The objectives of this study were (1) to compare the pendular mechanism (recovery, phase shift by α and β values, and ratio between forward and vertical mechanical work), the maximum possible elastic energy usage and the bilateral coordination during walking between non‐obese and obese children, and (2) to verify if the bilateral coordination could contribute to understanding the pendular mechanism and elastic energy usage in these populations. Nine obese (six female, 8.7 ± 0.5 years, 1.38 ± 0.04 m, 44.4 ± 6.3 kg and 24.1 ± 3.50 kg/m^2^) and eight non‐obese (four female, 7.4 ± 0.5 years, 1.31 ± 0.08 m, 26.6 ± 2.1 kg and 16.4 ± 1.40 kg/m^2^) children were analysed during walking on a treadmill at five speeds: 1, 2, 3, 4 and 5 km/h. The results indicated that although the mechanical energy response of the centre of mass during walking is similar between obese and non‐obese children, the obese children showed a lower pendulum‐like mechanism and greater elastic energy usage during level walking. Therefore, obese children seem to use more elastic energy during walking compared to non‐obese children, which may be related to their apparent higher positive work production during the double support phase. Finally, bilateral coordination presented high values at slow speeds in both groups and requires further attention due to its association with falls.

## Abstract (highlights)

- **What is the central question of this study?**
  Are there any differences of the pendular and elastic mechanisms and bilateral coordination during walking between non‐obese and obese children?
- **What is the main finding and its importance?**
  To our knowledge, this study is the first to analyse the mechanical energy usage and the bilateral coordination of obese and non‐obese children during walking. Obese children had a lower pendular recovery mechanism and used more elastic energy compared to non‐obese children. The bilateral coordination was higher at slow speeds in both groups and requires further attention due to its association with falls.

## 1 INTRODUCTION

Childhood obesity prevalence has grown, with increasing rates globally, mainly in middle‐ and low‐income countries (WHO, 2016), where the majority of obese children live. Obesity prevention is an international public health priority due to the negative impact of overweight on health and well‐being (Brown et al., 2019; WHO, 2016). As being overweight during childhood increases the odds of obesity during adult life, the prevention and treatment of childhood obesity are critical points for public health (Brown et al., 2019; WHO, 2016). Physical activity can reduce the risk of obesity in children from 6 to 12 years of age (Brown et al., 2019), and specifically, walking is a feasible physical activity to be implemented in the management of childhood obesity. Walking is an attractive and safe mode of aerobic training (Carlin et al., 2016), and it is a convenient form of daily physical activity that can be practiced in urban spaces (Kowaleski‐Jones et al., 2018).

Despite the indication of walking for weight management in obesity, the metabolic and mechanical responses to walking by obese people are not yet well understood. Obese adolescents (Peyrot et al., 2009) and obese adults (Browning et al., 2006; Mattsson et al., 1997) have greater energy expenditure compared to non‐obese counterparts during walking, whereas obese children seem to have similar walking energy expenditure to non‐obese children (Oliveira et al., 2020). Therefore, the greater energy expenditure of walking due to overweight could be associated with the maturation of the locomotor system, and this maturation occurs after the age of 10 (Schepens et al., 2004).

The mechanical determinants of walking energetics in obese individuals also are not fully understood. During walking, obese adults showed a recovery that was reduced at a self‐selected comfortable speed (Malatesta et al., 2009), unaltered at slow walking (2–4 km/h) (Menéndez et al., 2019) or increased at faster walking (5–6 km/h) (Menéndez et al., 2019). The use of an elastic mechanism to generate mechanical work during obese walking also seems to be influenced by the maturation of the locomotor system. The use of an elastic mechanism during walking can be inferred from the apparent mechanical efficiency, which is reduced in obese adolescents at slow speeds (2.7 and 3 km/h) (Peyrot et al., 2009) and in obese adults (Menéndez et al., 2019). On the other hand, obese adolescents at high speeds (4.5 and 5.4 km/h) (Peyrot et al., 2009) and obese children have unaltered efficiency compared to non‐obese counterparts (Oliveira et al., 2020). These findings suggest that obese children have a preserved elastic energy usage mechanism during walking, different from obese adults where this elastic mechanism is reduced and there is more reliance on the pendular mechanism (Menéndez et al., 2019).

The locomotor system can save mechanical energy during walking cycles due to its pendular behavior, transforming vertical mechanical energy into horizontal mechanical energy and vice versa along different phases of walking (Cavagna et al., 1976). The rate of conversion of one type of mechanical energy into another during a walking cycle can be measured by the recovery variable (Cavagna & Legramandi, 2020; Cavagna et al., 1976), which indicates the percentage of vertical mechanical energy that is being transformed into horizontal mechanical energy and vice versa.

The optimization of this conversion of one type of mechanical energy into another type is related to the phase shift between the vertical and horizontal mechanical energy curves, and their relative magnitude (Cavagna & Legramandi, 2020). The phase shift is the difference of phase between the curve's oscillation, and when the curves are oscillating completely out of phase the pendular mechanism is optimized (Cavagna & Legramandi, 2020). The phase shift can be measured by the α and β values, which indicate in degrees the synchronization between the maximum and minimum energy values of the mechanical curves (Cavagna & Legramandi, 2020). The relative magnitude of the mechanical energy curves can be verified by the ratio between forward (*W*_f_) and vertical (*W*_v_) mechanical work (Cavagna & Legramandi, 2020; Cavagna et al., 1983). The synchronization of timing and the magnitude of the pendulum‐like mechanism may be related to right–left stepping or bilateral coordination, as already observed indirectly in multiple sclerosis (Correale et al., 2022).

During walking an optimization of the storage energy and release of elastic energy may occur (Menéndez et al., 2019), which can be assessed by the maximum possible elastic energy usage (MPEEu) from the mechanical energy fluctuations of the body centre of mass (CoM) during the end of the contact phase. The MPEEu is based on the variation of the total mechanical energy of the body CoM during the contact phase, and it assess the mechanical energy absorbed during the single support phase and the energy released during the double contact phase (Menéndez et al., 2019). As this elastic energy usage is related to the temporal organization from the stride cycle, it may be that the coordination between lower limbs – assessed by the phase coordinative index (PCI) (Correale et al., 2022; Plotnik et al., 2007) – can influence on the usage of this stretch–shortening cycle.

The objectives of this study were (1) to compare the pendular mechanism (recovery, α, β, ratio *W*_f_/*W*_v_), the MPEEu and the PCI during walking between non‐obese and obese children, and (2) to verify if the PCI could help in understanding the pendular mechanism and elastic energy use in obese and non‐obese children.

The hypotheses were: (1) obese children would have a lower pendular mechanism and higher elastic energy usage (which could help to explain the similar metabolic energy expenditure found in (Oliveira et al., 2020), and (2) bilateral coordination is associated with the pendular mechanism and elastic energy usage.

## 2 METHODS

### 2.1 Ethical approval

The study was conducted in accordance with the latest revision of *Declaration of Helsinki* (October 2013), except for registration in a database. The dataset is available in Supporting information, Supplementary Material 1 and at FigShare (Ivaniski‐Mello et al., 2023). This study was approved by the Research Ethics Committee of the Universidade Federal do Rio Grande do Sul under protocol number 834854. The children and their respective legal guardians provided their written informed consent.

### 2.2 Participants and ethical aspects

Nine obese (OB) (six female, 8.7 ± 0.5 years, 1.38 ± 0.04 m, 44.4 ± 6.3 kg, and 24.1 ± 3.50 kg/m^2^) and eight non‐obese (Non‐OB) (four female, 7.4 ± 0.7 years, 1.31 ± 0.08 m, 26.6 ± 2.1 kg and 16.4 ± 1.40 kg/m^2^) children took part. The sample size of eight participants per group was calculated based on the recovery data of the study by Primavesi et al. (2021), using α of 0.05 and power of 0.90. The participants were chosen by convenience.

As inclusion criteria, the children should be between 7 and 9 years of age. This range was chosen because children younger than 6 years have very immature mechanics of walking; the differences between children and adults in walking disappear completely only around the age of 10 years (Cavagna et al., 1983; Schepens et al., 2004). The children included were all prebuscent youths, but we adopted the nomenclature of children (from 1 year through 12 years old) as suggested by the American Medical Association. Also, to participate the OB children had a body mass index percentile of ≥97, and the Non‐OB had a body mass index percentile >3 and <85 (De Onis et al., 2007). The exclusion criteria were any type of musculoskeletal injury, cardiovascular or respiratory diseases and/or diabetes.

### 2.3 Data collection

**FIGURE 1:**
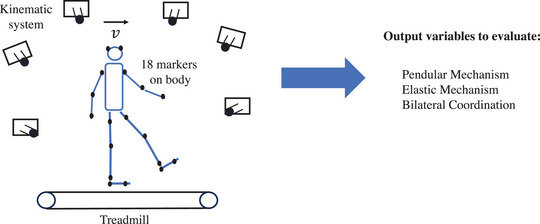
Schematic representation of the data collection set‐up, with the treadmill, kinematic capture system with six infra‐red cameras, and 18 reflexive markers placed on the participants’ body. The calculated variables were used to evaluate the pendular and elastic mechanism, and the bilateral coordination.

The children walked on a motorized treadmill (ATL Inbrasport, Medgraphics, Ann Arbor, MI, USA) at five speeds (1, 2, 3, 4, 5 km/h) in randomized order. The mean normalized walking speeds (Froude number) for the OB group were 0.01, 0.04, 0.10, 0.17 and 0.27, and for the Non‐OB group were 0.01, 0.05, 0.11, 0.19 and 0.30. In each speed condition, the children walked for 4 min (Oliveira et al., 2020), and during the last minute, the kinematic data were collected. The kinematic data were collected with six infrared cameras (200 Hz, Vicon, Oxford, UK) using 18 reflexive markers positioned bilaterally on the fifth metatarsal, calcaneus, lateral malleolus, femoral epicondyle, greater trochanter, acromion, lateral epicondyle of the humerus, midpoint of the distal radioulnar joint, and head (Minetti et al., 1993). Figure 1 shows a schematic model of the data collection and of the variables calculated.

### 2.4 Data analysis

The raw kinematic data were filtered with a low‐pass Butterworth filter (4° order, 5–10 Hz) and processed in a custom mathematical routine in MATLAB (v. 2020, The MathWorks Inc., Natick, MA, USA). The stride cycle events of foot touch‐down and take‐off were determined by a mathematical algorithm using foot marker position curves, and the quality of the events determination was also visually confirmed (Zeni et al., 2008). The stride cycle was initiated with the right foot touch‐down and lasted until the subsequent touch‐down of the same foot. The step cycle was initiated with the touch‐down of one foot and lasted until the touch‐down of the contralateral foot. The stride cycle was used to calculate the maximum possible elastic energy and the PCI, and the step cycle was used to calculate the pendular variables (recovery, α, β, *W*_f_/*W*_v_).

The CoM position was determined by a weighted average using a spatial body model of 11 rigid segments (Minetti et al., 1993) and an anthropometric data table for children (Schepens et al., 2001).

After the determination of the CoM position, the CoM mechanical energies were calculated (Cavagna & Kaneko, 1977; Minetti et al., 1993; Oliveira et al., 2020). The CoM position was differentiated in time to obtain the CoM speed in each axis, and then the antero‐posterior and vertical kinetic energy (*E*_kf_ and *E*_kv_, respectively) were calculated. The gravitational potential energy of the CoM (*E*_p_) was calculated with CoM height. The *E*_kv_ and *E*_p_ energies were summed to give the mechanical vertical of the CoM (*E*_v_), and the *E*_kf_ was considered the horizontal mechanical energy of the CoM. The sum of *E*_kf_ and *E*_v_ was the total mechanical energy (*E*_tot_). Finally, the summation of the positive increments during a step cycle of the *E*_kf_, *E*_v_ and *E*_tot_ resulted in the forward (*W*_f_), vertical (*W*_v_) and external (*W*_ext_) mechanical work, respectively.

#### 2.4.1 Pendular mechanism

To measure the capacity to recover mechanical energy during the gait cycles of walking by children, four parameters were calculated which are related to the capacity of the system to restore mechanical energy: the recovery (%), the phase shift between the vertical and horizontal mechanical energies curves (α and β in °), and the ratio between *W*_f_ and *W*_v_ (*W*_f_/*W*_v_, dimensionless).

The recovery (%) was calculated by Equation 1 (Cavagna et al., 1976), and represents the ability of the locomotor system to save mechanical energy during gait cycle due to its pendular behaviour:

(1) $$\mathrm{Recovery}\;=\frac{W_{f}+W_{v}-W_{\mathrm{ext}}}{W_{f}+W_{v}}\;\times 100$$

α and β represent the phase shift between the forward and vertical mechanical energy curves of the CoM during the walking cycle (Cavagna & Legramandi, 2020). α (in °) was calculated (Equation 2) from the time difference between the maximum *E*_f_ and the minimum *E*_v_ ($t_{\mathrm{pk}+}$) divided by the step time (ST) multiplied by 360°:

(2) $$\alpha =\frac{t_{\mathrm{pk}+}}{\mathrm{ST}}\;\times 360^{\circ }$$

β (in °) was calculated (Equation 3) similar to α, but taking into account the time difference between the minimum *E*_f_ and maximum *E*_v_ ($t_{\mathrm{pk}-}$):

(3) $$\beta =\frac{t_{\mathrm{pk}-}}{\mathrm{ST}}\;\times 360^{\circ }$$

α refers to the phase shift during the double contact phase, whereas β refers to the phase shift during the single contact phase. A positive α value suggests positive work being done to increase the kinetic energy and lift the CoM, and a positive β value indicates negative work being done to decelerate the body during the lowering of the CoM vertical position. The relative amplitude between *E_k_*_f_ and *E*_v_ curves during the walking step cycle was calculated from the ratio *W*_f_/*W*_v_ (Cavagna & Kaneko, 1977; Cavagna & Legramandi, 2020).

#### 2.4.2 Elastic energy

The capacity for storage and usage of elastic energy during walking can be measured with the parameter of maximum possible elastic energy usage (MPEEu) (Menéndez et al., 2019). The MPEEu has been used to estimate the storage and return of elastic energy during running on level and slope (Snyder & Farley, 2011; Snyder et al., 2012) and during adult obese walking (Menéndez et al., 2019). The negative energy fluctuation of the *E*_tot_ during the single support phase is considered the maximum possible elastic energy storage (MPEEs), and the positive energy fluctuation during the final double support phase is considered the maximum possible elastic energy released (MPEEr). The MPEEu during a stride cycle is the minimum value between MPEEs and MPEEr.

#### 2.4.3 Bilateral coordination

The coordination between lower limbs was measured with the PCI (Correale et al., 2022; Plotnik et al., 2007) using the step time and stride time. First, the φ value (in °) was calculated (Equation 4) for each gait cycle *i*:

(4) $$\varphi \left(i\right)=\frac{\mathrm{Step}\;\mathrm{time}\;\left({\mathrm{LL}}_{\mathrm{short}-\mathrm{swing}}\right)}{\mathrm{Stride}\;\mathrm{time}\;\left({\mathrm{LL}}_{\mathrm{long}-\mathrm{swing}}\right)}\;\times 360^{\circ }$$

where, LL_short‐swing_ and LL_long‐swing_ indicate the lower limb with shortest and longest swing time, respectively. Then, the accuracy (%) was calculated (Equation 5):

(5) $$\mathrm{Accuracy}\;=\frac{\left|\varphi -180\right|}{180}\;\times 100$$

Variability (%) was calculated with Equation 6:

(6) $$\mathrm{Variability}\;=\frac{\varphi_{\mathrm{SD}}}{\varphi_{\mathrm{mean}}}\;\times 100$$

Finally, PCI (%) was calculated by Equation 7:

(7) PCI=Accuracy+Variability

### 2.5 Statistical analyses

Generalized estimating equations (GEE) were used to test the effects of the group (obese and non‐obese), the walking speed and their interaction on the dependent variables. The covariance effects of the body size (lower limb length) on the main outcomes (recovery, MPEEu and PCI) were tested and were not statistically significant in all cases (*P* > 0.05). Therefore, for the sake of simplicity, only the results of the main effects are shown.

Pearson's correlation test was used to verify the correlation of PCI with the recovery and the MPEEu. The *r* coefficient was interpreted as negligible (0.00–0.30), low (0.30–0.50), moderate (0.50–0.70), high positive (0.70–0.90) and very high (0.90–1.00) (Hinkle et al., 2003). Second‐order polynomial regressions were calculated in the Python 3.8 programming language for each group separately, using the individual means from each child of the variables: recovery, MPEEu and PCI. All the analyses were performed in SPSS Statistics software (v. 22, IBM Corp., Armonk, NY, USA). The significance level adopted was α = 0.05.

## 3 RESULTS

All statistical results are provided in Supporting information, Supplementary Material 2.

### 3.1 Pendular mechanism

**FIGURE 2:**
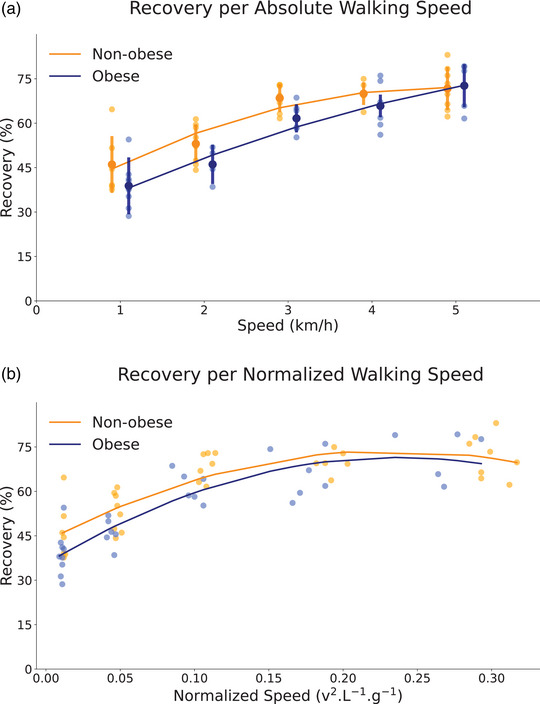
Recovery of mechanical energy by pendular mechanism of non‐obese and obese children as a function of absolute walking speed (1–5 km/h, a) and as function of Froude's number (squared walking speed normalized by lower limb length, L, and gravitational acceleration, g) (b). The large circles represent the group mean for each speed, and the error bars indicate the standard deviation. The small circles are the individual mean for each speed. The curves were estimated by a second‐order polynomial using the experimental data.

Figure 2 presents the recovery per walking speed of OB and Non‐OB children. The OB children had lower recovery compared to Non‐OB (*P* = 0.02), and both groups increased their recovery with the speed (*P* < 0.001). The α phase shift between the vertical and horizontal CoM energies was higher in OB children (*P* = 0.02), whereas the β phase shift was similar between groups (*P* = 0.51). The α and β reduced with the increase in speed (*P* < 0.001), but only the α reached negative values in the Non‐OB group (Table 1). The ratio between forward and vertical mechanical work (*W*_f_/*W*_v_) was greater at higher walking speeds (*P* < 0.001) and unaffected by the group (*P* = 0.58) (Table 1). The pendulum‐related variables had non‐significant interaction between group × speed (*P* = 0.11–0.98).

**TABLE 1:** Results (mean and SD) of α and β phase shift, the *W*_f_/*W*_v_ ratio, accuracy, and variability for non‐obese (Non‐OB) and obese (OB) children walking at different speeds.

		Speed (km/h)					*P*‐value		
Variables	Group	1	2	3	4	5	Group	Speed	Group × Speed
α (°)	Non‐OB	7.1 ± 18.0 ^a^	11.8 ± 15.7 ^a^	9.4 ± 19.0	0.0 ± 25.2 ^a^	−17.8 ± 13.5 ^b^	**0.016**	**<0.001**	0.107
	OB	20.8 ± 29.0	23.9 ± 9.5	14.0 ± 19.8	16.9 ± 12.7	8.0 ± 14.2			
β (°)	Non‐OB	24.2 ± 33.1 ^a, b^	28.8 ± 20.3 ^a^	9.2 ± 26.1 ^a, b^	8.3 ± 22.1 ^b^	0.6 ± 15.6 ^b^	0.507	**<0.001**	0.975
	OB	23.3 ± 35.1	34.8 ± 25.1	11.0 ± 17.6	16.8 ± 13.4	5.9 ± 8.5			
*W*_f_/*W*_v_ (ad)	Non‐OB	0.33 ± 0.10 ^a^	0.46 ± 0.12 ^b^	0.53 ± 0.09 ^b^	0.56 ± 0.09 ^c^	0.66 ± 0.11 ^c^	0.584	**<0.001**	0.230
	OB	0.34 ± 0.08	0.40 ± 0.13	0.49 ± 0.08	0.59 ± 0.10	0.64 ± 0.06			
Accuracy (%)	Non‐OB	2.5 ± 1.6 ^a^	2.3 ± 1.5 ^a^	1.1 ± 1.1 ^b^	1.4 ± 0.7 ^b^	0.9 ± 0.5 ^b^	0.571	**<0.001**	0.150
	OB	3.4 ± 1.7	2.9 ± 1.8	0.9 ± 0.8	1.1 ± 0.8	0.6 ± 0.5			
Variability (%)	Non‐OB	7.6 ± 3.6 ^a^	5.9 ± 4.0 ^b^	2.5 ± 1.7 ^b, c^	1.8 ± 0.6 ^c^	1.8 ± 0.8 ^c^	0.210	**<0.001**	**0.030**
	OB	6.2 ± 3.1 ^a^	3.2 ± 1.1 ^b^	2.7 ± 0.2 ^b^	2.5 ± 1.0 ^b^	1.4 ± 0.2 ^c^			

The *P*‐values for the factors group, speed and interaction group × speed are presented (in bold if *P* < 0.05). The superscript letters indicate statistically significant difference (*P* < 0.05) between the speeds in general (if interaction > 0.05) and within speeds between groups (if interaction < 0.05). Different letters indicate statistically significant difference between speeds (*P* < 0.05). Abbreviations: ad, adimensional unit; *W*_f_, forward mechanical work; *W*_v_, vertical mechanical work.

### 3.2 Elastic energy

**FIGURE 3:**
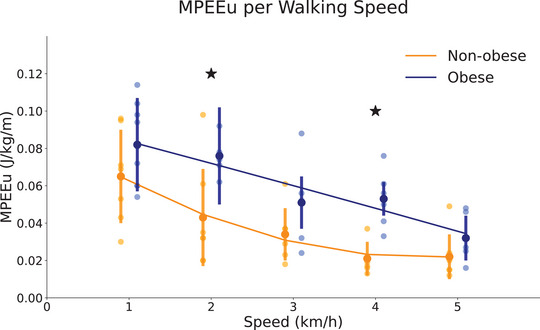
Maximum possible elastic energy usage (MPEEu) during walking by non‐obese and obese children walking at 1, 2, 3, 4 and 5 km/h. The large circles represent the group mean for each speed, and the error bars indicate the standard deviation. The small circles are the individual mean for each speed. The curves were estimated by a second‐order polynomial using the experimental data.

Figure 3 presents the MPEEu of each group at the different walking speeds. The MPEEs was unaffected by group (*P* = 0.23) and speed (*P* = 0.77). The MPEEr and MPEEu were greater in OB children (*P* < 0.001) and decreased with walking speed (*P* < 0.001). The interaction group × speed was significant only for MPEEu had significant interaction (*P* = 0.01), but not for MPEEs and MPEEr (*P* = 0.45 and *P* = 0.06, respectively).

### 3.3 Bilateral coordination

**FIGURE 4:**
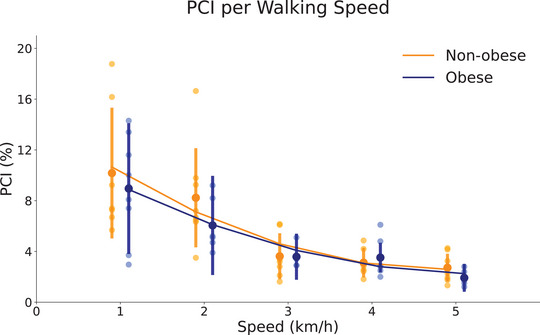
Phase coordinative index (PCI) during walking by non‐obese and obese children walking at 1, 2, 3, 4 and 5 km/h. The large circles represent the group mean for each speed, and the error bars indicate the standard deviation. The small circles are the individual mean for each speed. The curves were estimated by a second‐order polynomial using the experimental data.

The values of PCI (Figure 4), accuracy and variability (Table 1) were similar between groups (*P* = 0.29–0.57) and decreased with speed (*P* < 0.001). None of these coordinative variables had significant interaction group × speed (*P* = 0.15–0.38).

### 3.4 Correlation of bilateral coordination with pendular mechanism and elastic energy

The correlation tests between bilateral coordination (PCI) with pendular mechanism (recovery) and maximum possible elastic energy usage (MPEEu) indicated similar responses for groups of Non‐OB and OB children. The PCI was negatively correlated with recovery (Non‐OB: *r* = −0.61, *P* < 0.001; OB: *r* = −0.63, *P* < 0.001) and positively correlated with MPEEu (Non‐OB: *r* = 0.34, *P* = 0.04; OB: *r* = 0.49, *P* = 0.01).

## 4 DISCUSSION

In the present study, the pendular and elastic mechanisms and the bilateral coordination during walking were compared between obese and non‐obese children. The first hypothesis was confirmed showing that obese children slightly shift their walking pattern from a pendulum‐like to an elastic mechanism in comparison to non‐obese children. The second hypothesis was refuted because the bilateral coordination remained unchanged between obese and non‐obese children. Differently from obese adults, obese children walk with similar or lower pendulum‐like mechanisms partly explained by the less synchronized mechanical energies of the body centre of mass, particularly during the double stance phase of gait. To the best of our knowledge, this finding is the first report on factors that affect the pendular mechanism and elastic energy usage during walking in obese children.

Obese adults have a lower MPEE usage per kg of body mass than their lean counterparts, relying mostly on the pendular mechanism of walking, rather than on the storage and release of elastic energy, for decreasing the external mechanical work, and thus limiting the increase in the relative net energy cost of walking (Menéndez et al., 2019). In a recent paper, Oliveira et al. (2020) challenged these findings in obese children by demonstrating that total mechanical work was strictly related to the energy cost of walking. The present study shows that there is a worsening of the pendulum mechanism, at least at some speeds, and greater potential use of the elastic mechanism, resulting in compensatory effects that explain the similarity in the mechanical work produced per metre travelled and per kg of body mass carried. Probably this mechanism develops during growth. Instead, in severely obese adults, the pendulum mechanism seems to be enhanced (Primavesi et al., 2021). One mechanism involved in this improvement of the pendular mechanism might be related to the compliant characteristic of the fat tissue (Fu et al., 2015). This mechanical property of the fat tissue may cause an additional motion relative to the bone structure, similar to the wobbling mass motion already observed during jumping (Minetti & Belli, 1994). And, indeed, obese adults seem to perform this additional movement in comparison to non‐obese adults (Fu et al., 2015), justifying the higher pendular mechanism (Menéndez et al., 2019). Conversely, obese children may not generate this additional movement, resulting in a worse pendulum‐like walking mechanism compared to non‐obese children, but with an increased elastic energy usage. In other words, our study offers a reasonable explanation for the strict relationship between mechanical work and the energy cost of walking in obese children observed previously (Oliveira et al., 2020).

In general, the recovery values found in our study are similar to those of adults (Cavagna et al., 1976). One critical difference in walking mechanics related to the pendular mechanism is the forward and vertical work ratio, which in children ranged between ∼0.33 (1 km/h) and ∼0.66, showing lower values than adults (Cavagna & Legramandi, 2020) where the values are between ∼0.55 and ∼0.80 at the same speeds. Children seem to have a more bouncing gait, justifying the greater use of elastic energy, and thus generating more vertical work than horizontal proportionally, when compared to adults. To our knowledge, this is the first article that analyses the determinants of the pendulum mechanism and, consequently, the mechanical work and energy cost for walking in children. Another important mechanical difference between child and adult walking is in regard to the α and β phase shifts, which in children were more synchronized, finding perfect temporal synchronism at speed of 4 km/h, especially in α values, which indicate a better synchronization of the mechanical energies of the body centre of mass during double support in comparison to adult individuals (Gomeñuka et al., 2016).

Obese children a showed higher α phase shift than non‐obese children, which suggests a greater positive work during the double support phase by obese children (Cavagna & Legramandi, 2020). Given that the MPEEr and MPEEu of the obese children were higher, it is likely that elastic energy release performed this additional work during the double support phase. The pendular mechanism of the obese children also could be impaired by the fact that obese children have a longer double support time compared to non‐obese children to preserve stability (Boucher et al., 2015). Attention should be paid to this altered mechanics of walking by obese children, considering their increased risk of falls compared to non‐obese children due their altered postural control (Steinberg et al., 2018).

Although bilateral coordination was not different between the groups, the left–right step coordination was greatly influenced by speed. Obese and non‐obese children when walking at low speeds show impaired bilateral coordination with values even worse than subjects with balance impairment such as people with Parkinson's disease (Plotnik et al., 2007) and with multiple sclerosis (Correale et al., 2022; Plotnik et al., 2020). These are important findings given that obese children have a reduced effectiveness of mechanisms responsible for postural and movement control (Boucher et al., 2015) regardless of the bilateral coordination of gait. Previous findings have shown a higher step‐to‐step variability and low stability especially at slow speeds in obese children (McGraw et al., 2000). Thus, this outcome indicates special attention should be paid to low‐intensity locomotor activities in obese children.

Possible interference factors such as the measurement and analysis of lateral mechanical work (Tesio et al., 1998) and concomitant mechanical work during double contact (Donelan et al., 2002) have previously been clarified as unimportant for measures of external work and pendular mechanism in children (Bastien et al., 2003; Schepens et al., 2004). The present study has limitations that need to be discussed. First, the range of was limited to 7‐ to 9‐year‐old children, so the results cannot be extended to the entire childhood period. Also, this study does not provide primary functional results that would possibly be useful for future clinical trials. For future studies, it is suggested that how these mechanistic factors of gait performance relate to primary measures of functional mobility in obese and non‐obese children is verified.

### 4.1 Conclusions

The results indicated that although the mechanical energy response of the centre of mass during walking is similar between obese and non‐obese children, the obese children showed, on one side, a lower pendulum‐like mechanism. On the other side, obese children had more significant elastic energy usage during level walking. Therefore, obese children seem to use more elastic energy during walking compared to non‐obese children, which may be related to their apparent higher positive work production during the double support phase. Also, the timing of gravitational potential and kinetic energies was impaired during the double contact phase in obese in comparison to non‐obese children. Finally, bilateral coordination presented high values at slow speeds in both groups and requires further attention due to its association with falls.

## AUTHOR CONTRIBUTIONS

Leonardo Alexandre Peyré‐Tartaruga, Henrique Bianchi Oliveira, Arthur H. Dewolf, Cosme Franklim Buzzachera, Flávia Gomes Martinez and André Ivaniski‐Mello were involved with the conception. Leonardo Alexandre Peyré‐Tartaruga, Henrique Bianchi Oliveira and André Ivaniski‐Mello were involved with the data analysis and drafting. Leonardo Alexandre Peyré‐Tartaruga and Henrique Bianchi Oliveira were responsible for data acquisition. Leonardo Alexandre Peyré‐Tartaruga, Henrique Bianchi Oliveira, Arthur H. Dewolf, Cosme Franklim Buzzachera, Flávia Gomes Martinez and André Ivaniski‐Mello were responsible for results interpretation and critically revising important intellectual content. All persons designated as authors qualify for authorship, and all those who qualify for authorship are listed. All authors approved the final version of the manuscript and agree to be accountable for all aspects of the work in ensuring that questions related to the accuracy or integrity of any part of the work are appropriately investigated and resolved.

## CONFLICT OF INTEREST

The authors declare no conflict of interest.

## Supporting information

Supplementary Material 1

Supplementary Material 2

## Data Availability

The dataset with the individual data is available in Supplementary Material 1 and in FigShare (Ivaniski‐Mello et al., 2023) at https://doi.org/10.6084/m9.figshare.23667075.v1.
