# Supplementary material for: Pendular mechanism determinants and elastic energy usage during walking of obese and non‐obese children
Source: Exp Physiol. 2023 Sep 18;108(11):1400–8. doi: 10.1113/EP091408 (PMC10988495; doi:10.1113/EP091408)
Supplement: Supplementary file 2 — Supplementary Material 2 [file EPH-108-1400-s001.pdf]

Supplementary Material 2
Statistical
Output Sumamry SPSS software Title: Pendular mechanism determinants and elastic energy usage during walking of obese and non-obese children Authors: Peyré-Tartaruga et al. This documents includes all statistical procedures: 1. GEE for comparison
2. Pearson's correlation test

```
 * Generalized Estimating Equations.
GENLIN Recovery BY Group Speed (ORDER=ASCENDING)
  /MODEL Group Speed Group*Speed INTERCEPT=YES
 DISTRIBUTION=NORMAL LINK=IDENTITY
  /CRITERIA SCALE=MLE PCONVERGE=1E-006(ABSOLUTE) SINGULAR=1E-012 ANALYSISTY
PE=3(WALD) CILEVEL=95
    LIKELIHOOD=FULL
  /EMMEANS TABLES=Group SCALE=ORIGINAL COMPARE=Group CONTRAST=PAIRWISE PADJ
UST=BONFERRONI
  /EMMEANS TABLES=Speed SCALE=ORIGINAL COMPARE=Speed CONTRAST=PAIRWISE PADJ
UST=BONFERRONI
  /EMMEANS TABLES=Group*Speed SCALE=ORIGINAL COMPARE=Group CONTRAST=PAIRWIS
E
    PADJUST=BONFERRONI
      /EMMEANS TABLES=Group*Speed SCALE=ORIGINAL COMPARE=Speed CONTRAST=PAI
RWISE
    PADJUST=BONFERRONI
  /REPEATED SUBJECT=Subject SORT=YES CORRTYPE=INDEPENDENT ADJUSTCORR=YES CO
VB=ROBUST
  /MISSING CLASSMISSING=EXCLUDE
  /PRINT CPS DESCRIPTIVES MODELINFO FIT SUMMARY SOLUTION.
```

Generalized Linear Models

Supplementary Material 2
Statistical
Output Sumamry SPSS software Title: Pendular mechanism determinants and elastic energy usage during walking of obese and non-obese children Authors: Peyré-Tartaruga et al. This documents includes all statistical procedures: 1. GEE for comparison
2. Pearson's correlation test

Notes

Output Created		12-MAY-2023 12:20:46
Comments		
Input	Data	C: \Users\andre\Documents\Andre\Pesquisa\Artigos para Publicar\Henrique Bianchi Mestrado\Statistics\Statistics Sheets\Statistics_Sheet.sav
	Active Dataset	DataSet1
	Filter	<none>
	Weight	<none>
	Split File	<none>
	N of Rows in Working Data File	68
Missing Value Handling	Definition of Missing	User-defined missing values for factor, subject and within-subject variables are treated as missing.
	Cases Used	Statistics are based on cases with valid data for all variables in the model.
Weight Handling		not applicable

Supplementary Material 2
Statistical
Output Sumamry SPSS software Title: Pendular mechanism determinants and elastic energy usage during walking of obese and non-obese children Authors: Peyré-Tartaruga et al. This documents includes all statistical procedures: 1. GEE for comparison
2. Pearson's correlation test

Notes

Syntax	GENLIN Recovery BY Group Speed (ORDER=ASCENDING)<br>/MODEL Group Speed Group*Speed INTERCEPT=YES<br><br>DISTRIBUTION=NORMAL LINK=IDENTITY<br>/CRITERIA SCALE=MLE PCONVERGE=1E-006 (ABSOLUTE) SINGULAR=1E-012 ANALYSISTYPE=3 (WALD) CILEVEL=95<br>LIKELIHOOD=FULL<br>/EMMEANS TABLES=Group SCALE=ORIGINAL COMPARE=Group CONTRAST=PAIRWISE PADJUST=BONFERRONI<br>/EMMEANS TABLES=Speed SCALE=ORIGINAL COMPARE=Speed CONTRAST=PAIRWISE PADJUST=BONFERRONI<br>/EMMEANS TABLES=Group*Speed SCALE=ORIGINAL COMPARE=Group CONTRAST=PAIRWISE<br><br>PADJUST=BONFERRONI<br>/EMMEANS TABLES=Group*Speed SCALE=ORIGINAL COMPARE=Speed CONTRAST=PAIRWISE<br><br>PADJUST=BONFERRONI<br>/REPEATED SUBJECT=Subject SORT=YES CORRTYPE=INDEPENDENT ADJUSTCORR=YES COVB=ROBUST<br>/MISSING CLASSMISSING=EXCLUDE<br>/PRINT CPS DESCRIPTIVES MODELINFO FIT SUMMARY SOLUTION.

Supplementary Material 2
Statistical
Output Sumamry SPSS software Title: Pendular mechanism determinants and elastic energy usage during walking of obese and non-obese children Authors: Peyré-Tartaruga et al. This documents includes all statistical procedures: 1. GEE for comparison
2. Pearson's correlation test

Notes

Resources	Processor Time	00:00:00.05
	Elapsed Time	00:00:00.06

Model Information

Dependent Variable		Recovery (%)
Probability Distribution		Normal
Link Function		Identity
Subject Effect	1	Subject
Working Correlation Matrix Structure		Independent

Case Processing Summary

	N	Percent
Included	68	100.0%
Excluded	0	0.0%
Total	68	100.0%

Correlated Data Summary

Number of Levels	Subject Effect	Subject	17
Number of Subjects			17
Number of Measurements per Subject	Minimum		2
	Maximum		5
Correlation Matrix Dimension			5

Categorical Variable Information

			N	Percent
Factor	Group	EUT	36	52.9%
		OB	32	47.1%
		Total	68	100.0%
	Speed	1	16	23.5%
		2	14	20.6%
		3	13	19.1%
		4	12	17.6%
		5	13	19.1%
		Total	68	100.0%

Continuous Variable Information

		N	Minimum	Maximum	Mean	Std. Deviation
Dependent Variable	Recovery (%)	68	28.7	83.1	58.441	13.7730

Goodness of Fit[a]

	Value
Quasi Likelihood under Independence Model Criterion (QIC)[b]	2742.916
Corrected Quasi Likelihood under Independence Model Criterion (QICC)[b]	2743.240

Dependent Variable: Recovery (%)
Model: (Intercept), Group, Speed, Group * Speed[a]

a. Information criteria are in smaller-is-better form.

b. Computed using the full log quasi-likelihood function.

Tests of Model Effects

Source	Type III		
	Wald Chi-Square	df	Sig.
(Intercept)	3377.347	1	.000
Group	5.597	1	.018
Speed	277.048	4	.000
Group * Speed	3.963	4	.411

Dependent Variable: Recovery (%)
Model: (Intercept), Group, Speed, Group * Speed

Parameter Estimates

Parameter	B	Std. Error	95% Wald Confidence Interval		Hypothesis Test
			Lower	Upper	Wald Chi-Square
(Intercept)	72.660	3.3353	66.123	79.197	474.597
[Group=1]	-.949	4.1117	-9.008	7.110	.053
[Group=2]	0[a]	.	.	.	.
[Speed=1]	-33.801	3.6677	-40.990	-26.613	84.934
[Speed=2]	-26.568	3.1844	-32.810	-20.327	69.609
[Speed=3]	-11.018	3.4333	-17.747	-4.289	10.299
[Speed=4]	-6.840	3.8926	-14.469	.789	3.088
[Speed=5]	0[a]	.	.	.	.
[Group=1] * [Speed=1]	8.138	5.7677	-3.166	19.443	1.991
[Group=1] * [Speed=2]	7.903	4.0162	.032	15.775	3.872
[Group=1] * [Speed=3]	7.793	4.6272	-1.276	16.862	2.836
[Group=1] * [Speed=4]	5.104	4.5301	-3.775	13.983	1.269
[Group=1] * [Speed=5]	0[a]	.	.	.	.
[Group=2] * [Speed=1]	0[a]	.	.	.	.
[Group=2] * [Speed=2]	0[a]	.	.	.	.
[Group=2] * [Speed=3]	0[a]	.	.	.	.
[Group=2] * [Speed=4]	0[a]	.	.	.	.
[Group=2] * [Speed=5]	0[a]	.	.	.	.
(Scale)	46.952				

Parameter Estimates

	Hypothesis Test	
Parameter	df	Sig.
(Intercept)	1	.000
[Group=1]	1	.818
[Group=2]	.	.
[Speed=1]	1	.000
[Speed=2]	1	.000
[Speed=3]	1	.001
[Speed=4]	1	.079
[Speed=5]	.	.
[Group=1] * [Speed=1]	1	.158
[Group=1] * [Speed=2]	1	.049
[Group=1] * [Speed=3]	1	.092
[Group=1] * [Speed=4]	1	.260
[Group=1] * [Speed=5]	.	.
[Group=2] * [Speed=1]	.	.
[Group=2] * [Speed=2]	.	.
[Group=2] * [Speed=3]	.	.
[Group=2] * [Speed=4]	.	.
[Group=2] * [Speed=5]	.	.
(Scale)		

Dependent Variable: Recovery (%)
Model: (Intercept), Group, Speed, Group * Speed

a. Set to zero because this parameter is redundant.

Estimated Marginal Means 1: Group

Estimates

			95% Wald Confidence Interval	
Group	Mean	Std. Error	Lower	Upper
EUT	61.853	1.3142	59.278	64.429
OB	57.014	1.5673	53.943	60.086

Pairwise Comparisons

(I) Group	(J) Group	Mean Difference (I-J)	Std. Error	df	Bonferroni Sig.	95% Wald Confidence ... Lower
EUT	OB	4.839[a]	2.0454	1	.018	.830
OB	EUT	-4.839[a]	2.0454	1	.018	-8.848

Pairwise Comparisons

(I) Group	(J) Group	95% Wald Confidence ... Upper
EUT	OB	8.848
OB	EUT	-.830

Pairwise comparisons of estimated marginal means based on the original scale of dependent variable Recovery (%)

a. The mean difference is significant at the .05 level.

Overall Test Results

Wald Chi-Square	df	Sig.
5.597	1	.018

The Wald chi-square tests the effect of Group. This test is based on the linearly independent pairwise comparisons among the estimated marginal means.

Estimated Marginal Means 2: Speed

Estimates

Speed	Mean	Std. Error	95% Wald Confidence Interval	
			Lower	Upper
1	42.454	2.0471	38.442	46.466
2	49.569	1.3945	46.836	52.302
3	65.064	1.2582	62.598	67.530
4	67.898	1.6694	64.625	71.170
5	72.186	2.0559	68.156	76.215

Pairwise Comparisons

(I) Speed	(J) Speed	Mean Difference (I-J)	Std. Error	df	Bonferroni Sig.	95% Wald Confidence ...
						Lower
1	2	-7.115[a]	2.0617	1	.006	-12.902
	3	-22.610[a]	1.8801	1	.000	-27.888
	4	-25.444[a]	2.5884	1	.000	-32.709
	5	-29.732[a]	2.8839	1	.000	-37.827
2	1	7.115[a]	2.0617	1	.006	1.328
	3	-15.495[a]	1.5127	1	.000	-19.741
	4	-18.329[a]	2.1819	1	.000	-24.453
	5	-22.617[a]	2.0081	1	.000	-28.254
3	1	22.610[a]	1.8801	1	.000	17.332
	2	15.495[a]	1.5127	1	.000	11.249
	4	-2.834	1.7736	1	1.000	-7.812
	5	-7.122[a]	2.3136	1	.021	-13.616
4	1	25.444[a]	2.5884	1	.000	18.178
	2	18.329[a]	2.1819	1	.000	12.204
	3	2.834	1.7736	1	1.000	-2.145
	5	-4.288	2.2650	1	.583	-10.646
5	1	29.732[a]	2.8839	1	.000	21.637
	2	22.617[a]	2.0081	1	.000	16.980
	3	7.122[a]	2.3136	1	.021	.628
	4	4.288	2.2650	1	.583	-2.070

Pairwise Comparisons

(I) Speed	(J) Speed	95% Wald Confidence ... Upper
1	2	-1.328
	3	-17.332
	4	-18.178
	5	-21.637
2	1	12.902
	3	-11.249
	4	-12.204
	5	-16.980
3	1	27.888
	2	19.741
	4	2.145
	5	-.628
4	1	32.709
	2	24.453
	3	7.812
	5	2.070
5	1	37.827
	2	28.254
	3	13.616
	4	10.646

Pairwise comparisons of estimated marginal means based on the original scale of dependent variable Recovery (%)

a. The mean difference is significant at the .05 level.

Overall Test Results

Wald Chi-Square	df	Sig.
277.048	4	.000

The Wald chi-square tests the effect of Speed. This test is based on the linearly independent pairwise comparisons among the estimated marginal means.

Estimated Marginal Means 3: Group* Speed

Estimates

Group	Speed	Mean	Std. Error	95% Wald Confidence Interval Lower	95% Wald Confidence Interval Upper
EUT	1	46.049	3.3588	39.466	52.632
	2	53.046	2.1811	48.771	57.321
	3	68.486	1.6596	65.233	71.738
	4	69.975	1.4242	67.184	72.766
	5	71.711	2.4046	66.998	76.424
OB	1	38.859	2.3412	34.270	43.447
	2	46.092	1.7380	42.685	49.498
	3	61.642	1.8916	57.934	65.349
	4	65.820	3.0199	59.901	71.739
	5	72.660	3.3353	66.123	79.197

Pairwise Comparisons

Speed	(I) Group	(J) Group	Mean Difference (I-J)	Std. Error	df	Bonferroni Sig.
1	EUT	OB	7.190	4.0942	1	.079
	OB	EUT	-7.190	4.0942	1	.079
2	EUT	OB	6.955[a]	2.7889	1	.013
	OB	EUT	-6.955[a]	2.7889	1	.013
3	EUT	OB	6.844[a]	2.5164	1	.007
	OB	EUT	-6.844[a]	2.5164	1	.007
4	EUT	OB	4.155	3.3389	1	.213
	OB	EUT	-4.155	3.3389	1	.213
5	EUT	OB	-.949	4.1117	1	.818
	OB	EUT	.949	4.1117	1	.818

Pairwise Comparisons

Speed	(I) Group	(J) Group	95% Wald Confidence Interval for Difference	
			Lower	Upper
1	EUT	OB	-.835	15.214
	OB	EUT	-15.214	.835
2	EUT	OB	1.488	12.421
	OB	EUT	-12.421	-1.488
3	EUT	OB	1.912	11.776
	OB	EUT	-11.776	-1.912
4	EUT	OB	-2.389	10.699
	OB	EUT	-10.699	2.389
5	EUT	OB	-9.008	7.110
	OB	EUT	-7.110	9.008

Pairwise comparisons of estimated marginal means based on the original scale of dependent variable Recovery (%)

a. The mean difference is significant at the .05 level.

Overall Test Results

Speed	Wald Chi-Square	df	Sig.
1	3.084	1	.079
2	6.218	1	.013
3	7.397	1	.007
4	1.549	1	.213
5	.053	1	.818

Each Wald chi-square tests the simple effects of Group within each level combination of the other factors shown. These tests are based on the linearly independent pairwise comparisons among the estimated marginal means.

Estimated Marginal Means 4: Group* Speed

Estimates

Group	Speed	Mean	Std. Error	95% Wald Confidence Interval	
				Lower	Upper
EUT	1	46.049	3.3588	39.466	52.632
	2	53.046	2.1811	48.771	57.321
	3	68.486	1.6596	65.233	71.738
	4	69.975	1.4242	67.184	72.766
	5	71.711	2.4046	66.998	76.424
OB	1	38.859	2.3412	34.270	43.447
	2	46.092	1.7380	42.685	49.498
	3	61.642	1.8916	57.934	65.349
	4	65.820	3.0199	59.901	71.739
	5	72.660	3.3353	66.123	79.197

Pairwise Comparisons

Group	(I) Speed	(J) Speed	Mean Difference (I-J)	Std. Error	df	Bonferroni Sig.
EUT	1	2	-6.998	2.5631	1	.063
		3	-22.437[a]	2.6823	1	.000
		4	-23.926[a]	4.3391	1	.000
		5	-25.663[a]	4.4514	1	.000
	2	1	6.998	2.5631	1	.063
		3	-15.439[a]	2.2416	1	.000
		4	-16.929[a]	2.5305	1	.000
		5	-18.665[a]	2.4474	1	.000
	3	1	22.437[a]	2.6823	1	.000
		2	15.439[a]	2.2416	1	.000
		4	-1.489	2.2406	1	1.000
		5	-3.226	3.1022	1	1.000
	4	1	23.926[a]	4.3391	1	.000
		2	16.929[a]	2.5305	1	.000
		3	1.489	2.2406	1	1.000
		5	-1.736	2.3171	1	1.000
	5	1	25.663[a]	4.4514	1	.000
		2	18.665[a]	2.4474	1	.000
		3	3.226	3.1022	1	1.000
		4	1.736	2.3171	1	1.000
OB	1	2	-7.233	3.2299	1	.251
		3	-22.783[a]	2.6353	1	.000
		4	-26.961[a]	2.8232	1	.000
		5	-33.801[a]	3.6677	1	.000
	2	1	7.233	3.2299	1	.251
		3	-15.550[a]	2.0318	1	.000
		4	-19.728[a]	3.5552	1	.000
		5	-26.568[a]	3.1844	1	.000

Pairwise Comparisons

Group	(I) Speed	(J) Speed	95% Wald Confidence Interval for Difference	
			Lower	Upper
EUT	1	2	-14.193	.197
		3	-29.966	-14.908
		4	-36.106	-11.746
		5	-38.158	-13.168
	2	1	-.197	14.193
		3	-21.732	-9.147
		4	-24.032	-9.826
		5	-25.535	-11.795
	3	1	14.908	29.966
		2	9.147	21.732
		4	-7.779	4.800
		5	-11.934	5.483
	4	1	11.746	36.106
		2	9.826	24.032
		3	-4.800	7.779
		5	-8.241	4.768
	5	1	13.168	38.158
		2	11.795	25.535
		3	-5.483	11.934
		4	-4.768	8.241
OB	1	2	-16.299	1.834
		3	-30.180	-15.385
		4	-34.886	-19.036
		5	-44.096	-23.506
	2	1	-1.834	16.299
		3	-21.253	-9.847
		4	-29.708	-9.749
		5	-35.507	-17.630

Pairwise Comparisons

Group	(I) Speed	(J) Speed	Mean Difference (I-J)	Std. Error	df	Bonferroni Sig.
	3	1	22.783[a]	2.6353	1	.000
		2	15.550[a]	2.0318	1	.000
		4	-4.178	2.7499	1	1.000
		5	-11.018[a]	3.4333	1	.013
	4	1	26.961[a]	2.8232	1	.000
		2	19.728[a]	3.5552	1	.000
		3	4.178	2.7499	1	1.000
		5	-6.840	3.8926	1	.789
	5	1	33.801[a]	3.6677	1	.000
		2	26.568[a]	3.1844	1	.000
		3	11.018[a]	3.4333	1	.013
		4	6.840	3.8926	1	.789

Pairwise Comparisons

Group	(I) Speed	(J) Speed	95% Wald Confidence Interval for Difference	
			Lower	Upper
	3	1	15.385	30.180
		2	9.847	21.253
		4	-11.897	3.541
		5	-20.656	-1.381
	4	1	19.036	34.886
		2	9.749	29.708
		3	-3.541	11.897
		5	-17.767	4.087
	5	1	23.506	44.096
		2	17.630	35.507
		3	1.381	20.656
		4	-4.087	17.767

Supplementary Material 2
Statistical
Output Sumamry SPSS software Title: Pendular mechanism determinants and elastic energy usage during walking of obese and non-obese children Authors: Peyré-Tartaruga et al. This documents includes all statistical procedures: 1. GEE for comparison

Pairwise comparisons of estimated marginal means based on the original scale of dependent variable Recovery (%)

a. The mean difference is significant at the .05 level.

Overall Test Results

Group	Wald Chi-Square	df	Sig.
EUT	109.121	4	.000
OB	235.416	4	.000

Each Wald chi-square tests the simple effects of Speed within each level combination of the other factors shown. These tests are based on the linearly independent pairwise comparisons among the estimated marginal means.

```
 * Generalized Estimating Equations.
GENLIN Alfa BY Group Speed (ORDER=ASCENDING)
  /MODEL Group Speed Group*Speed INTERCEPT=YES
 DISTRIBUTION=NORMAL LINK=IDENTITY
  /CRITERIA SCALE=MLE PCONVERGE=1E-006(ABSOLUTE) SINGULAR=1E-012 ANALYSISTY
PE=3(WALD) CILEVEL=95
    LIKELIHOOD=FULL
  /EMMEANS TABLES=Group SCALE=ORIGINAL COMPARE=Group CONTRAST=PAIRWISE PADJ
UST=BONFERRONI
  /EMMEANS TABLES=Speed SCALE=ORIGINAL COMPARE=Speed CONTRAST=PAIRWISE PADJ
UST=BONFERRONI
  /EMMEANS TABLES=Group*Speed SCALE=ORIGINAL COMPARE=Group CONTRAST=PAIRWIS
E
    PADJUST=BONFERRONI
      /EMMEANS TABLES=Group*Speed SCALE=ORIGINAL COMPARE=Speed CONTRAST=PAI
RWISE
    PADJUST=BONFERRONI
  /REPEATED SUBJECT=Subject SORT=YES CORRTYPE=INDEPENDENT ADJUSTCORR=YES CO
VB=ROBUST
  /MISSING CLASSMISSING=EXCLUDE
```

```
/PRINT CPS DESCRIPTIVES MODELINFO FIT SUMMARY SOLUTION.
```

Generalized Linear Models

Notes

Output Created		12-MAY-2023 12:20:46
Comments		
Input	Data	C:<br>\Users\andre\Documents\Andre\Pesquisa\Artigos para Publicar\Henrique Bianchi Mestrado\Statistics\Statistics Sheets\Statistics_Sheet.sav
	Active Dataset	DataSet1
	Filter	<none>
	Weight	<none>
	Split File	<none>
	N of Rows in Working Data File	68
Missing Value Handling	Definition of Missing	User-defined missing values for factor, subject and within-subject variables are treated as missing.
	Cases Used	Statistics are based on cases with valid data for all variables in the model.
Weight Handling		not applicable

Supplementary Material 2
Statistical
Output Sumamry SPSS software Title: Pendular mechanism determinants and elastic energy usage during walking of obese and non-obese children Authors: Peyré-Tartaruga et al. This documents includes all statistical procedures: 1. GEE for comparison
2. Pearson's correlation test

Notes

Syntax	GENLIN Alfa BY Group Speed (ORDER=ASCENDING)<br>/MODEL Group Speed Group*Speed INTERCEPT=YES<br>DISTRIBUTION=NORMAL LINK=IDENTITY<br>/CRITERIA SCALE=MLE PCONVERGE=1E-006 (ABSOLUTE) SINGULAR=1E-012 ANALYSISTYPE=3 (WALD) CILEVEL=95<br>LIKELIHOOD=FULL<br>/EMMEANS TABLES=Group SCALE=ORIGINAL COMPARE=Group CONTRAST=PAIRWISE PADJUST=BONFERRONI<br>/EMMEANS TABLES=Speed SCALE=ORIGINAL COMPARE=Speed CONTRAST=PAIRWISE PADJUST=BONFERRONI<br>/EMMEANS TABLES=Group*Speed SCALE=ORIGINAL COMPARE=Group CONTRAST=PAIRWISE<br>PADJUST=BONFERRONI<br>/EMMEANS TABLES=Group*Speed SCALE=ORIGINAL COMPARE=Speed CONTRAST=PAIRWISE<br>PADJUST=BONFERRONI<br>/REPEATED SUBJECT=Subject SORT=YES CORRTYPE=INDEPENDENT ADJUSTCORR=YES COVB=ROBUST<br>/MISSING CLASSMISSING=EXCLUDE<br>/PRINT CPS DESCRIPTIVES MODELINFO FIT SUMMARY SOLUTION.

Notes

Resources	Processor Time	00:00:00.05
	Elapsed Time	00:00:00.06

Model Information

Dependent Variable		Alfa (°)
Probability Distribution		Normal
Link Function		Identity
Subject Effect	1	Subject
Working Correlation Matrix Structure		Independent

Case Processing Summary

	N	Percent
Included	68	100.0%
Excluded	0	0.0%
Total	68	100.0%

Correlated Data Summary

Number of Levels	Subject Effect	Subject	17
Number of Subjects			17
Number of Measurements per Subject	Minimum		2
	Maximum		5
Correlation Matrix Dimension			5

Supplementary Material 2
Statistical
Output Sumamry SPSS software Title: Pendular mechanism determinants and elastic energy usage during walking of obese and non-obese children Authors: Peyré-Tartaruga et al. This documents includes all statistical procedures: 1. GEE for comparison
2. Pearson's correlation test

Categorical Variable Information

			N	Percent
Factor	Group	EUT	36	52.9%
		OB	32	47.1%
		Total	68	100.0%
	Speed	1	16	23.5%
		2	14	20.6%
		3	13	19.1%
		4	12	17.6%
		5	13	19.1%
		Total	68	100.0%

Continuous Variable Information

		N	Minimum	Maximum	Mean	Std. Deviation
Dependent Variable	Alfa (°)	68	-31.1	67.4	9.160	21.3264

Goodness of Fit[a]

	Value
Quasi Likelihood under Independence Model Criterion (QIC)[b]	21058.053
Corrected Quasi Likelihood under Independence Model Criterion (QICC)[b]	21059.077

Dependent Variable: Alfa (°)
Model: (Intercept), Group, Speed, Group * Speed[a]

a. Information criteria are in smaller-is-better form.

b. Computed using the full log quasi-likelihood function.

Tests of Model Effects

Source	Type III		
	Wald Chi-Square	df	Sig.
(Intercept)	9.569	1	.002
Group	5.784	1	.016
Speed	54.555	4	.000
Group * Speed	7.617	4	.107

Dependent Variable: Alfa (°)
Model: (Intercept), Group, Speed, Group * Speed

Parameter Estimates

Parameter	B	Std. Error	95% Wald Confidence Interval		Hypothesis Test
			Lower	Upper	Wald Chi-Square
(Intercept)	7.996	5.6639	-3.105	19.097	1.993
[Group=1]	-25.831	7.2091	-39.961	-11.701	12.839
[Group=2]	0[a]	.	.	.	.
[Speed=1]	12.792	6.6884	-.317	25.901	3.658
[Speed=2]	15.862	4.6086	6.830	24.895	11.847
[Speed=3]	6.047	8.9183	-11.432	23.527	.460
[Speed=4]	8.876	6.6407	-4.140	21.891	1.786
[Speed=5]	0[a]	.	.	.	.
[Group=1] * [Speed=1]	12.146	8.8596	-5.218	29.511	1.880
[Group=1] * [Speed=2]	13.763	6.3145	1.387	26.139	4.750
[Group=1] * [Speed=3]	21.148	10.3668	.829	41.466	4.161
[Group=1] * [Speed=4]	9.003	11.7207	-13.969	31.975	.590
[Group=1] * [Speed=5]	0[a]	.	.	.	.
[Group=2] * [Speed=1]	0[a]	.	.	.	.
[Group=2] * [Speed=2]	0[a]	.	.	.	.
[Group=2] * [Speed=3]	0[a]	.	.	.	.
[Group=2] * [Speed=4]	0[a]	.	.	.	.
[Group=2] * [Speed=5]	0[a]	.	.	.	.
(Scale)	362.743				

Parameter Estimates

Parameter	Hypothesis Test df	Sig.
(Intercept)	1	.158
[Group=1]	1	.000
[Group=2]	.	.
[Speed=1]	1	.056
[Speed=2]	1	.001
[Speed=3]	1	.498
[Speed=4]	1	.181
[Speed=5]	.	.
[Group=1] * [Speed=1]	1	.170
[Group=1] * [Speed=2]	1	.029
[Group=1] * [Speed=3]	1	.041
[Group=1] * [Speed=4]	1	.442
[Group=1] * [Speed=5]	.	.
[Group=2] * [Speed=1]	.	.
[Group=2] * [Speed=2]	.	.
[Group=2] * [Speed=3]	.	.
[Group=2] * [Speed=4]	.	.
[Group=2] * [Speed=5]	.	.
(Scale)		

Dependent Variable: Alfa (°)
Model: (Intercept), Group, Speed, Group * Speed

a. Set to zero because this parameter is redundant.

Estimated Marginal Means 1: Group

Estimates

Group	Mean	Std. Error	95% Wald Confidence Interval Lower	Upper
EUT	2.092	4.3090	-6.353	10.538
OB	16.711	4.2874	8.308	25.115

Pairwise Comparisons

(I) Group	(J) Group	Mean Difference (I-J)	Std. Error	df	Bonferroni Sig.	95% Wald Confidence ... Lower	95% Wald Confidence ... Upper
EUT	OB	-14.619[a]	6.0786	1	.016	-26.533	-2.705
OB	EUT	14.619[a]	6.0786	1	.016	2.705	26.533

Pairwise comparisons of estimated marginal means based on the original scale of dependent variable Alfa (°)

a. The mean difference is significant at the .05 level.

Overall Test Results

Wald Chi-Square	df	Sig.
5.784	1	.016

The Wald chi-square tests the effect of Group. This test is based on the linearly independent pairwise comparisons among the estimated marginal means.

Estimated Marginal Means 2: Speed

Estimates

Speed	Mean	Std. Error	95% Wald Confidence Interval Lower	95% Wald Confidence Interval Upper
1	13.945	5.5442	3.079	24.812
2	17.824	3.1383	11.673	23.975
3	11.702	4.9650	1.970	21.433
4	8.458	5.2550	-1.842	18.757
5	-4.920	3.6046	-11.984	2.145

Pairwise Comparisons

(I) Speed	(J) Speed	Mean Difference (I-J)	Std. Error	df	Bonferroni Sig.	95% Wald Confidence ... Lower
1	2	-3.879	3.6402	1	1.000	-14.097
	3	2.244	6.4372	1	1.000	-15.826
	4	5.488	7.6096	1	1.000	-15.873
	5	18.865[a]	4.4298	1	.000	6.430
2	1	3.879	3.6402	1	1.000	-6.339
	3	6.123	5.4235	1	1.000	-9.101
	4	9.367	5.9603	1	1.000	-7.364
	5	22.744[a]	3.1572	1	.000	13.881
3	1	-2.244	6.4372	1	1.000	-20.313
	2	-6.123	5.4235	1	1.000	-21.346
	4	3.244	5.4733	1	1.000	-12.120
	5	16.621[a]	5.1834	1	.013	2.071
4	1	-5.488	7.6096	1	1.000	-26.848
	2	-9.367	5.9603	1	1.000	-26.098
	3	-3.244	5.4733	1	1.000	-18.608
	5	13.377	5.8603	1	.225	-3.073
5	1	-18.865[a]	4.4298	1	.000	-31.299
	2	-22.744[a]	3.1572	1	.000	-31.606
	3	-16.621[a]	5.1834	1	.013	-31.171
	4	-13.377	5.8603	1	.225	-29.827

Pairwise Comparisons

(I) Speed	(J) Speed	95% Wald Confidence ... Upper
1	2	6.339
	3	20.313
	4	26.848
	5	31.299
2	1	14.097
	3	21.346
	4	26.098
	5	31.606
3	1	15.826
	2	9.101
	4	18.608
	5	31.171
4	1	15.873
	2	7.364
	3	12.120
	5	29.827
5	1	-6.430
	2	-13.881
	3	-2.071
	4	3.073

Pairwise comparisons of estimated marginal means based on the original scale of dependent variable Alfa (°)

a. The mean difference is significant at the .05 level.

Overall Test Results

Wald Chi-Square	df	Sig.
54.555	4	.000

The Wald chi-square tests the effect of Speed. This test is based on the linearly independent pairwise comparisons among the estimated marginal means.

Estimated Marginal Means 3: Group* Speed

Estimates

Group	Speed	Mean	Std. Error	95% Wald Confidence Interval	
				Lower	Upper
EUT	1	7.103	6.2965	-5.238	19.444
	2	11.790	5.1943	1.609	21.971
	3	9.360	6.6424	-3.659	22.379
	4	.043	9.3840	-18.349	18.436
	5	-17.835	4.4601	-26.577	-9.093
OB	1	20.788	9.1272	2.899	38.677
	2	23.858	3.5233	16.953	30.764
	3	14.043	7.3813	-.424	28.510
	4	16.872	4.7328	7.596	26.148
	5	7.996	5.6639	-3.105	19.097

Pairwise Comparisons

Speed	(I) Group	(J) Group	Mean Difference (I-J)	Std. Error	df	Bonferroni Sig.
1	EUT	OB	-13.685	11.0883	1	.217
	OB	EUT	13.685	11.0883	1	.217
2	EUT	OB	-12.068	6.2765	1	.055
	OB	EUT	12.068	6.2765	1	.055
3	EUT	OB	-4.683	9.9300	1	.637
	OB	EUT	4.683	9.9300	1	.637
4	EUT	OB	-16.828	10.5100	1	.109
	OB	EUT	16.828	10.5100	1	.109
5	EUT	OB	-25.831[a]	7.2091	1	.000
	OB	EUT	25.831[a]	7.2091	1	.000

Pairwise Comparisons

Speed	(I) Group	(J) Group	95% Wald Confidence Interval for Difference	
			Lower	Upper
1	EUT	OB	-35.418	8.048
	OB	EUT	-8.048	35.418
2	EUT	OB	-24.370	.233
	OB	EUT	-.233	24.370
3	EUT	OB	-24.146	14.779
	OB	EUT	-14.779	24.146
4	EUT	OB	-37.428	3.771
	OB	EUT	-3.771	37.428
5	EUT	OB	-39.961	-11.701
	OB	EUT	11.701	39.961

Pairwise comparisons of estimated marginal means based on the original scale of dependent variable Alfa (°)

a. The mean difference is significant at the .05 level.

Overall Test Results

Speed	Wald Chi-Square	df	Sig.
1	1.523	1	.217
2	3.697	1	.055
3	.222	1	.637
4	2.564	1	.109
5	12.839	1	.000

Each Wald chi-square tests the simple effects of Group within each level combination of the other factors shown. These tests are based on the linearly independent pairwise comparisons among the estimated marginal means.

Estimated Marginal Means 4: Group* Speed

Estimates

Group	Speed	Mean	Std. Error	95% Wald Confidence Interval	
				Lower	Upper
EUT	1	7.103	6.2965	-5.238	19.444
	2	11.790	5.1943	1.609	21.971
	3	9.360	6.6424	-3.659	22.379
	4	.043	9.3840	-18.349	18.436
	5	-17.835	4.4601	-26.577	-9.093
OB	1	20.788	9.1272	2.899	38.677
	2	23.858	3.5233	16.953	30.764
	3	14.043	7.3813	-.424	28.510
	4	16.872	4.7328	7.596	26.148
	5	7.996	5.6639	-3.105	19.097

Pairwise Comparisons

Group	(I) Speed	(J) Speed	Mean Difference (I-J)	Std. Error	df	Bonferroni Sig.
EUT	1	2	-4.687	2.6868	1	.811
		3	-2.257	7.0911	1	1.000
		4	7.060	12.1088	1	1.000
		5	24.938[a]	5.8101	1	.000
	2	1	4.687	2.6868	1	.811
		3	2.430	6.4122	1	1.000
		4	11.747	10.1851	1	1.000
		5	29.625[a]	4.3167	1	.000
	3	1	2.257	7.0911	1	1.000
		2	-2.430	6.4122	1	1.000
		4	9.317	10.3314	1	1.000
		5	27.195[a]	5.2854	1	.000
	4	1	-7.060	12.1088	1	1.000
		2	-11.747	10.1851	1	1.000
		3	-9.317	10.3314	1	1.000
		5	17.878	9.6580	1	.641
	5	1	-24.938[a]	5.8101	1	.000
		2	-29.625[a]	4.3167	1	.000
		3	-27.195[a]	5.2854	1	.000
		4	-17.878	9.6580	1	.641
OB	1	2	-3.071	6.7664	1	1.000
		3	6.744	10.7456	1	1.000
		4	3.916	9.2198	1	1.000
		5	12.792	6.6884	1	.558
	2	1	3.071	6.7664	1	1.000
		3	9.815	8.7488	1	1.000
		4	6.987	6.1941	1	1.000
		5	15.862[a]	4.6086	1	.006
	3	1	-6.744	10.7456	1	1.000
		2	-9.815	8.7488	1	1.000

Pairwise Comparisons

Group	(I) Speed	(J) Speed	95% Wald Confidence Interval for Difference	
			Lower	Upper
EUT	1	2	-12.229	2.855
		3	-22.162	17.648
		4	-26.930	41.049
		5	8.629	41.247
	2	1	-2.855	12.229
		3	-15.569	20.429
		4	-16.843	40.337
		5	17.508	41.742
	3	1	-17.648	22.162
		2	-20.429	15.569
		4	-19.684	38.317
		5	12.359	42.031
	4	1	-41.049	26.930
		2	-40.337	16.843
		3	-38.317	19.684
		5	-9.232	44.989
	5	1	-41.247	-8.629
		2	-41.742	-17.508
		3	-42.031	-12.359
		4	-44.989	9.232
OB	1	2	-22.064	15.923
		3	-23.419	36.908
		4	-21.964	29.796
		5	-5.983	31.566
	2	1	-15.923	22.064
		3	-14.743	34.373
		4	-10.400	24.374
		5	2.926	28.799
	3	1	-36.908	23.419
		2	-34.373	14.743

Pairwise Comparisons

Group	(I) Speed	(J) Speed	Mean Difference (I-J)	Std. Error	df	Bonferroni Sig.
		4	-2.828	3.6182	1	1.000
		5	6.047	8.9183	1	1.000
	4	1	-3.916	9.2198	1	1.000
		2	-6.987	6.1941	1	1.000
		3	2.828	3.6182	1	1.000
		5	8.876	6.6407	1	1.000
	5	1	-12.792	6.6884	1	.558
		2	-15.862[a]	4.6086	1	.006
		3	-6.047	8.9183	1	1.000
		4	-8.876	6.6407	1	1.000

Pairwise Comparisons

Group	(I) Speed	(J) Speed	95% Wald Confidence Interval for Difference	
			Lower	Upper
		4	-12.985	7.328
		5	-18.987	31.081
	4	1	-29.796	21.964
		2	-24.374	10.400
		3	-7.328	12.985
		5	-9.765	27.516
	5	1	-31.566	5.983
		2	-28.799	-2.926
		3	-31.081	18.987
		4	-27.516	9.765

Pairwise comparisons of estimated marginal means based on the original scale of dependent variable Alfa (°)

a. The mean difference is significant at the .05 level.

Overall Test Results

Group	Wald Chi-Square	df	Sig.
EUT	106.466	4	.000
OB	13.028	4	.011

Each Wald chi-square tests the simple effects of Speed within each level combination of the other factors shown. These tests are based on the linearly independent pairwise comparisons among the estimated marginal means.

```
GENLIN Beta BY Group Speed (ORDER=ASCENDING)
  /MODEL Group Speed Group*Speed INTERCEPT=YES
 DISTRIBUTION=NORMAL LINK=IDENTITY
  /CRITERIA SCALE=MLE PCONVERGE=1E-006(ABSOLUTE) SINGULAR=1E-012 ANALYSISTY
PE=3(WALD) CILEVEL=95
    LIKELIHOOD=FULL
  /EMMEANS TABLES=Group SCALE=ORIGINAL COMPARE=Group CONTRAST=PAIRWISE PADJ
UST=BONFERRONI
  /EMMEANS TABLES=Speed SCALE=ORIGINAL COMPARE=Speed CONTRAST=PAIRWISE PADJ
UST=BONFERRONI
  /EMMEANS TABLES=Group*Speed SCALE=ORIGINAL COMPARE=Group*Speed CONTRAST=P
AIRWISE
    PADJUST=BONFERRONI
  /REPEATED SUBJECT=Subject SORT=YES CORRTYPE=INDEPENDENT ADJUSTCORR=YES CO
VB=ROBUST
  /MISSING CLASSMISSING=EXCLUDE
  /PRINT CPS DESCRIPTIVES MODELINFO FIT SUMMARY SOLUTION.
```

```
GENLIN Ratio_Wf_Wv BY Group Speed (ORDER=ASCENDING)
  /MODEL Group Speed Group*Speed INTERCEPT=YES
 DISTRIBUTION=NORMAL LINK=IDENTITY
  /CRITERIA SCALE=MLE PCONVERGE=1E-006(ABSOLUTE) SINGULAR=1E-012 ANALYSISTY
PE=3(WALD) CILEVEL=95
```

```
    LIKELIHOOD=FULL
  /EMMEANS TABLES=Group SCALE=ORIGINAL COMPARE=Group CONTRAST=PAIRWISE PADJ
UST=BONFERRONI
  /EMMEANS TABLES=Speed SCALE=ORIGINAL COMPARE=Speed CONTRAST=PAIRWISE PADJ
UST=BONFERRONI
  /EMMEANS TABLES=Group*Speed SCALE=ORIGINAL COMPARE=Group CONTRAST=PAIRWIS
E
    PADJUST=BONFERRONI
      /EMMEANS TABLES=Group*Speed SCALE=ORIGINAL COMPARE=Speed CONTRAST=PAI
RWISE
    PADJUST=BONFERRONI
  /REPEATED SUBJECT=Subject SORT=YES CORRTYPE=INDEPENDENT ADJUSTCORR=YES CO
VB=ROBUST
  /MISSING CLASSMISSING=EXCLUDE
  /PRINT CPS DESCRIPTIVES MODELINFO FIT SUMMARY SOLUTION.
```

```
 GENLIN MPEEUsaged BY Group Speed (ORDER=ASCENDING)
  /MODEL Group Speed Group*Speed INTERCEPT=YES
 DISTRIBUTION=NORMAL LINK=IDENTITY
  /CRITERIA SCALE=MLE PCONVERGE=1E-006(ABSOLUTE) SINGULAR=1E-012 ANALYSISTY
PE=3(WALD) CILEVEL=95
    LIKELIHOOD=FULL
  /EMMEANS TABLES=Group SCALE=ORIGINAL COMPARE=Group CONTRAST=PAIRWISE PADJ
UST=BONFERRONI
  /EMMEANS TABLES=Speed SCALE=ORIGINAL COMPARE=Speed CONTRAST=PAIRWISE PADJ
UST=BONFERRONI
  /EMMEANS TABLES=Group*Speed SCALE=ORIGINAL COMPARE=Group CONTRAST=PAIRWIS
E
    PADJUST=BONFERRONI
      /EMMEANS TABLES=Group*Speed SCALE=ORIGINAL COMPARE=Speed CONTRAST=PAI
RWISE
    PADJUST=BONFERRONI
  /REPEATED SUBJECT=Subject SORT=YES CORRTYPE=INDEPENDENT ADJUSTCORR=YES CO
VB=ROBUST
  /MISSING CLASSMISSING=EXCLUDE
  /PRINT CPS DESCRIPTIVES MODELINFO FIT SUMMARY SOLUTION.
```

Supplementary Material 2
Statistical
Output Sumamry SPSS software Title: Pendular mechanism determinants and elastic energy usage during walking of obese and non-obese children Authors: Peyré-Tartaruga et al. This documents includes all statistical procedures: 1. GEE for comparison
2. Pearson's correlation test

```
GENLIN Phi BY Group Speed (ORDER=ASCENDING)
  /MODEL Group Speed Group*Speed INTERCEPT=YES
 DISTRIBUTION=NORMAL LINK=IDENTITY
  /CRITERIA SCALE=MLE PCONVERGE=1E-006(ABSOLUTE) SINGULAR=1E-012 ANALYSISTY
PE=3(WALD) CILEVEL=95
    LIKELIHOOD=FULL
  /EMMEANS TABLES=Group SCALE=ORIGINAL COMPARE=Group CONTRAST=PAIRWISE PADJ
UST=BONFERRONI
  /EMMEANS TABLES=Speed SCALE=ORIGINAL COMPARE=Speed CONTRAST=PAIRWISE PADJ
UST=BONFERRONI
  /EMMEANS TABLES=Group*Speed SCALE=ORIGINAL COMPARE=Group*Speed CONTRAST=P
AIRWISE
    PADJUST=BONFERRONI
  /REPEATED SUBJECT=Subject SORT=YES CORRTYPE=INDEPENDENT ADJUSTCORR=YES CO
VB=ROBUST
  /MISSING CLASSMISSING=EXCLUDE
  /PRINT CPS DESCRIPTIVES MODELINFO FIT SUMMARY SOLUTION.
```

Generalized Linear Models

Notes

Output Created		12-MAY-2023 12:20:46
Comments		
Input	Data	C:\Users\andre\Documents\Andre\Pesquisa\Artigos para Publicar\Henrique Bianchi Mestrado\Statistics\Statistics Sheets\Statistics_Sheet.sav
	Active Dataset	DataSet1
	Filter	<none>
	Weight	<none>
	Split File	<none>
	N of Rows in Working Data File	68
Missing Value Handling	Definition of Missing	User-defined missing values for factor, subject and within-subject variables are treated as missing.
	Cases Used	Statistics are based on cases with valid data for all variables in the model.
Weight Handling		not applicable

Notes

Syntax	GENLIN Phi BY Group Speed (ORDER=ASCENDING)<br>/MODEL Group Speed Group*Speed INTERCEPT=YES<br><br>DISTRIBUTION=NORMAL LINK=IDENTITY<br>/CRITERIA SCALE=MLE PCONVERGE=1E-006 (ABSOLUTE) SINGULAR=1E-012 ANALYSISTYPE=3 (WALD) CILEVEL=95<br>LIKELIHOOD=FULL<br>/EMMEANS TABLES=Group SCALE=ORIGINAL COMPARE=Group CONTRAST=PAIRWISE PADJUST=BONFERRONI<br>/EMMEANS TABLES=Speed SCALE=ORIGINAL COMPARE=Speed CONTRAST=PAIRWISE PADJUST=BONFERRONI<br>/EMMEANS TABLES=Group*Speed SCALE=ORIGINAL COMPARE=Group*Speed CONTRAST=PAIRWISE<br><br>PADJUST=BONFERRONI<br>/REPEATED SUBJECT=Subject SORT=YES CORRTYPE=INDEPENDENT ADJUSTCORR=YES COVB=ROBUST<br>/MISSING CLASSMISSING=EXCLUDE<br>/PRINT CPS DESCRIPTIVES MODELINFO FIT SUMMARY SOLUTION.

Notes

Resources	Processor Time	00:00:00.06
	Elapsed Time	00:00:00.06

Model Information

Dependent Variable		Phi (°)
Probability Distribution		Normal
Link Function		Identity
Subject Effect	1	Subject
Working Correlation Matrix Structure		Independent

Case Processing Summary

	N	Percent
Included	68	100.0%
Excluded	0	0.0%
Total	68	100.0%

Correlated Data Summary

Number of Levels	Subject Effect	Subject	17
Number of Subjects			17
Number of Measurements per Subject	Minimum		2
	Maximum		5
Correlation Matrix Dimension			5

Categorical Variable Information

			N	Percent
Factor	Group	EUT	36	52.9%
		OB	32	47.1%
		Total	68	100.0%
	Speed	1	16	23.5%
		2	14	20.6%
		3	13	19.1%
		4	12	17.6%
		5	13	19.1%
		Total	68	100.0%

Continuous Variable Information

		N	Minimum	Maximum	Mean	Std. Deviation
Dependent Variable	Phi (°)	68	174.1	190.3	181.598	3.9325

Goodness of Fit[a]

	Value
Quasi Likelihood under Independence Model Criterion (QIC)[b]	914.168
Corrected Quasi Likelihood under Independence Model Criterion (QICC)[b]	915.519

Dependent Variable: Phi (°)
Model: (Intercept), Group, Speed, Group * Speed[a]

a. Information criteria are in smaller-is-better form.

b. Computed using the full log quasi-likelihood function.

Tests of Model Effects

Source	Type III		
	Wald Chi-Square	df	Sig.
(Intercept)	133527.803	1	.000
Group	.172	1	.678
Speed	9.771	4	.044
Group * Speed	1.531	4	.821

Dependent Variable: Phi (°)
Model: (Intercept), Group, Speed, Group * Speed

Parameter Estimates

Parameter	B	Std. Error	95% Wald Confidence Interval		Hypothesis Test
			Lower	Upper	Wald Chi-Square
(Intercept)	180.580	.5118	179.577	181.583	124500.368
[Group=1]	-.800	.8204	-2.408	.808	.951
[Group=2]	0[a]	.	.	.	.
[Speed=1]	3.275	2.1701	-.978	7.529	2.278
[Speed=2]	1.853	2.3202	-2.694	6.401	.638
[Speed=3]	.853	.6507	-.422	2.129	1.720
[Speed=4]	-.480	.6843	-1.821	.861	.492
[Speed=5]	0[a]	.	.	.	.
[Group=1] * [Speed=1]	-.198	2.8017	-5.690	5.293	.005
[Group=1] * [Speed=2]	.965	3.0392	-4.991	6.922	.101
[Group=1] * [Speed=3]	.964	1.1012	-1.194	3.122	.766
[Group=1] * [Speed=4]	.208	1.2519	-2.245	2.662	.028
[Group=1] * [Speed=5]	0[a]	.	.	.	.
[Group=2] * [Speed=1]	0[a]	.	.	.	.
[Group=2] * [Speed=2]	0[a]	.	.	.	.
[Group=2] * [Speed=3]	0[a]	.	.	.	.
[Group=2] * [Speed=4]	0[a]	.	.	.	.
[Group=2] * [Speed=5]	0[a]	.	.	.	.
(Scale)	15.440				

Parameter Estimates

Parameter	Hypothesis Test df	Sig.
(Intercept)	1	.000
[Group=1]	1	.329
[Group=2]	.	.
[Speed=1]	1	.131
[Speed=2]	1	.424
[Speed=3]	1	.190
[Speed=4]	1	.483
[Speed=5]	.	.
[Group=1] * [Speed=1]	1	.944
[Group=1] * [Speed=2]	1	.751
[Group=1] * [Speed=3]	1	.381
[Group=1] * [Speed=4]	1	.868
[Group=1] * [Speed=5]	.	.
[Group=2] * [Speed=1]	.	.
[Group=2] * [Speed=2]	.	.
[Group=2] * [Speed=3]	.	.
[Group=2] * [Speed=4]	.	.
[Group=2] * [Speed=5]	.	.
(Scale)		

Dependent Variable: Phi (°)
Model: (Intercept), Group, Speed, Group * Speed

a. Set to zero because this parameter is redundant.

Estimated Marginal Means 1: Group

Estimates

Group	Mean	Std. Error	95% Wald Confidence Interval Lower	Upper
EUT	181.268	.7396	179.819	182.718
OB	181.680	.6630	180.381	182.980

Pairwise Comparisons

(I) Group	(J) Group	Mean Difference (I-J)	Std. Error	df	Bonferroni Sig.	95% Wald Confidence ... Lower
EUT	OB	-.412	.9933	1	.678	-2.359
OB	EUT	.412	.9933	1	.678	-1.535

Pairwise Comparisons

(I) Group	(J) Group	95% Wald Confidence ... Upper
EUT	OB	1.535
OB	EUT	2.359

Pairwise comparisons of estimated marginal means based on the original scale of dependent variable Phi (°)

Overall Test Results

Wald Chi-Square	df	Sig.
.172	1	.678

The Wald chi-square tests the effect of Group. This test is based on the linearly independent pairwise comparisons among the estimated marginal means.

Estimated Marginal Means 2: Speed

Estimates

Speed	Mean	Std. Error	95% Wald Confidence Interval Lower	Upper
1	183.356	1.2515	180.903	185.809
2	182.516	1.3466	179.877	185.155
3	181.515	.5129	180.510	182.521
4	179.804	.7209	178.391	181.217
5	180.180	.4102	179.376	180.984

Pairwise Comparisons

(I) Speed	(J) Speed	Mean Difference (I-J)	Std. Error	df	Bonferroni Sig.	95% Wald Confidence ...
						Lower
1	2	.840	1.6380	1	1.000	-3.758
	3	1.841	1.1295	1	1.000	-1.329
	4	3.552	1.3747	1	.098	-.307
	5	3.176	1.4008	1	.234	-.756
2	1	-.840	1.6380	1	1.000	-5.438
	3	1.001	1.4599	1	1.000	-3.097
	4	2.712	1.4142	1	.552	-1.258
	5	2.336	1.5196	1	1.000	-1.929
3	1	-1.841	1.1295	1	1.000	-5.012
	2	-1.001	1.4599	1	1.000	-5.099
	4	1.711	.7356	1	.200	-.354
	5	1.335	.5506	1	.153	-.210
4	1	-3.552	1.3747	1	.098	-7.411
	2	-2.712	1.4142	1	.552	-6.682
	3	-1.711	.7356	1	.200	-3.776
	5	-.376	.6259	1	1.000	-2.133
5	1	-3.176	1.4008	1	.234	-7.109
	2	-2.336	1.5196	1	1.000	-6.602
	3	-1.335	.5506	1	.153	-2.881
	4	.376	.6259	1	1.000	-1.381

Pairwise Comparisons

(I) Speed	(J) Speed	95% Wald Confidence ... Upper
1	2	5.438
	3	5.012
	4	7.411
	5	7.109
2	1	3.758
	3	5.099
	4	6.682
	5	6.602
3	1	1.329
	2	3.097
	4	3.776
	5	2.881
4	1	.307
	2	1.258
	3	.354
	5	1.381
5	1	.756
	2	1.929
	3	.210
	4	2.133

Pairwise comparisons of estimated marginal means based on the original scale of dependent variable Phi (°)

Overall Test Results

Wald Chi-Square	df	Sig.
9.771	4	.044

The Wald chi-square tests the effect of Speed. This test is based on the linearly independent pairwise comparisons among the estimated marginal means.

Supplementary Material 2
Statistical
Output Sumamry SPSS software Title: Pendular mechanism determinants and elastic energy usage during walking of obese and non-obese children Authors: Peyré-Tartaruga et al. This documents includes all statistical procedures: 1. GEE for comparison 2. Pearson's correlation test

Estimated Marginal Means 3: Group* Speed

Estimates

Group	Speed	Mean	Std. Error	95% Wald Confidence Interval	
				Lower	Upper
EUT	1	182.857	1.6652	179.593	186.121
	2	182.599	1.4851	179.688	185.510
	3	181.597	.7995	180.030	183.164
	4	179.508	1.0915	177.369	181.648
	5	179.780	.6412	178.523	181.037
OB	1	183.855	1.8688	180.193	187.518
	2	182.433	2.2468	178.030	186.837
	3	181.433	.6428	180.174	182.693
	4	180.100	.9419	178.254	181.946
	5	180.580	.5118	179.577	181.583

Pairwise Comparisons

(I) Group*Speed	(J) Group*Speed	Mean Difference (I-J)	Std. Error	df
[Group=1]*[Speed=1]	[Group=1]*[Speed=2]	.258	1.7662	1
	[Group=1]*[Speed=3]	1.260	1.3893	1
	[Group=1]*[Speed=4]	3.349	1.2622	1
	[Group=1]*[Speed=5]	3.077	1.7720	1
	[Group=2]*[Speed=1]	-.998	2.5030	1
	[Group=2]*[Speed=2]	.424	2.7966	1
	[Group=2]*[Speed=3]	1.424	1.7849	1
	[Group=2]*[Speed=4]	2.757	1.9131	1
	[Group=2]*[Speed=5]	2.277	1.7421	1
[Group=1]*[Speed=2]	[Group=1]*[Speed=1]	-.258	1.7662	1
	[Group=1]*[Speed=3]	1.002	1.7422	1
	[Group=1]*[Speed=4]	3.090	1.5582	1
	[Group=1]*[Speed=5]	2.819	1.9629	1
	[Group=2]*[Speed=1]	-1.257	2.3870	1
	[Group=2]*[Speed=2]	.165	2.6933	1

Pairwise Comparisons

(I) Group*Speed	(J) Group*Speed	Bonferroni Sig.	95% Wald Confidence Interval for Difference	
			Lower	Upper
[Group=1]*[Speed=1]	[Group=1]*[Speed=2]	1.000	-5.501	6.018
	[Group=1]*[Speed=3]	1.000	-3.270	5.790
	[Group=1]*[Speed=4]	.359	-.767	7.464
	[Group=1]*[Speed=5]	1.000	-2.701	8.855
	[Group=2]*[Speed=1]	1.000	-9.160	7.163
	[Group=2]*[Speed=2]	1.000	-8.695	9.543
	[Group=2]*[Speed=3]	1.000	-4.396	7.244
	[Group=2]*[Speed=4]	1.000	-3.481	8.995
	[Group=2]*[Speed=5]	1.000	-3.403	7.958
[Group=1]*[Speed=2]	[Group=1]*[Speed=1]	1.000	-6.018	5.501
	[Group=1]*[Speed=3]	1.000	-4.679	6.682
	[Group=1]*[Speed=4]	1.000	-1.991	8.171
	[Group=1]*[Speed=5]	1.000	-3.582	9.219
	[Group=2]*[Speed=1]	1.000	-9.040	6.527
	[Group=2]*[Speed=2]	1.000	-8.617	8.948

Pairwise Comparisons

(I) Group*Speed	(J) Group*Speed	Mean Difference (I-J)	Std. Error	df
	[Group=2]*[Speed=3]	1.165	1.6183	1
	[Group=2]*[Speed=4]	2.499	1.7586	1
	[Group=2]*[Speed=5]	2.019	1.5708	1
[Group=1]*[Speed=3]	[Group=1]*[Speed=1]	-1.260	1.3893	1
	[Group=1]*[Speed=2]	-1.002	1.7422	1
	[Group=1]*[Speed=4]	2.089	1.1999	1
	[Group=1]*[Speed=5]	1.817	.8883	1
	[Group=2]*[Speed=1]	-2.258	2.0326	1
	[Group=2]*[Speed=2]	-.836	2.3848	1
	[Group=2]*[Speed=3]	.164	1.0258	1
	[Group=2]*[Speed=4]	1.497	1.2355	1
	[Group=2]*[Speed=5]	1.017	.9492	1
[Group=1]*[Speed=4]	[Group=1]*[Speed=1]	-3.349	1.2622	1
	[Group=1]*[Speed=2]	-3.090	1.5582	1
	[Group=1]*[Speed=3]	-2.089	1.1999	1
	[Group=1]*[Speed=5]	-.272	1.0483	1
	[Group=2]*[Speed=1]	-4.347	2.1642	1
	[Group=2]*[Speed=2]	-2.925	2.4979	1
	[Group=2]*[Speed=3]	-1.925	1.2667	1
	[Group=2]*[Speed=4]	-.592	1.4417	1
	[Group=2]*[Speed=5]	-1.072	1.2055	1
[Group=1]*[Speed=5]	[Group=1]*[Speed=1]	-3.077	1.7720	1
	[Group=1]*[Speed=2]	-2.819	1.9629	1
	[Group=1]*[Speed=3]	-1.817	.8883	1
	[Group=1]*[Speed=4]	.272	1.0483	1
	[Group=2]*[Speed=1]	-4.075	1.9757	1
	[Group=2]*[Speed=2]	-2.653	2.3365	1
	[Group=2]*[Speed=3]	-1.653	.9079	1
	[Group=2]*[Speed=4]	-.320	1.1395	1
	[Group=2]*[Speed=5]	-.800	.8204	1

Pairwise Comparisons

(I) Group*Speed	(J) Group*Speed	Bonferroni Sig.	95% Wald Confidence Interval for Difference Lower	Upper
	[Group=2]*[Speed=3]	1.000	-4.111	6.442
	[Group=2]*[Speed=4]	1.000	-3.236	8.233
	[Group=2]*[Speed=5]	1.000	-3.103	7.141
[Group=1]*[Speed=3]	[Group=1]*[Speed=1]	1.000	-5.790	3.270
	[Group=1]*[Speed=2]	1.000	-6.682	4.679
	[Group=1]*[Speed=4]	1.000	-1.824	6.002
	[Group=1]*[Speed=5]	1.000	-1.079	4.714
	[Group=2]*[Speed=1]	1.000	-8.886	4.370
	[Group=2]*[Speed=2]	1.000	-8.612	6.940
	[Group=2]*[Speed=3]	1.000	-3.181	3.509
	[Group=2]*[Speed=4]	1.000	-2.531	5.526
	[Group=2]*[Speed=5]	1.000	-2.078	4.112
[Group=1]*[Speed=4]	[Group=1]*[Speed=1]	.359	-7.464	.767
	[Group=1]*[Speed=2]	1.000	-8.171	1.991
	[Group=1]*[Speed=3]	1.000	-6.002	1.824
	[Group=1]*[Speed=5]	1.000	-3.690	3.147
	[Group=2]*[Speed=1]	1.000	-11.404	2.710
	[Group=2]*[Speed=2]	1.000	-11.070	5.220
	[Group=2]*[Speed=3]	1.000	-6.055	2.205
	[Group=2]*[Speed=4]	1.000	-5.293	4.109
	[Group=2]*[Speed=5]	1.000	-5.003	2.859
[Group=1]*[Speed=5]	[Group=1]*[Speed=1]	1.000	-8.855	2.701
	[Group=1]*[Speed=2]	1.000	-9.219	3.582
	[Group=1]*[Speed=3]	1.000	-4.714	1.079
	[Group=1]*[Speed=4]	1.000	-3.147	3.690
	[Group=2]*[Speed=1]	1.000	-10.518	2.367
	[Group=2]*[Speed=2]	1.000	-10.272	4.965
	[Group=2]*[Speed=3]	1.000	-4.614	1.307
	[Group=2]*[Speed=4]	1.000	-4.035	3.395
	[Group=2]*[Speed=5]	1.000	-3.475	1.875

Pairwise Comparisons

(I) Group*Speed	(J) Group*Speed	Mean Difference (I-J)	Std. Error	df
[Group=2]*[Speed=1]	[Group=1]*[Speed=1]	.998	2.5030	1
	[Group=1]*[Speed=2]	1.257	2.3870	1
	[Group=1]*[Speed=3]	2.258	2.0326	1
	[Group=1]*[Speed=4]	4.347	2.1642	1
	[Group=1]*[Speed=5]	4.075	1.9757	1
	[Group=2]*[Speed=2]	1.422	2.7591	1
	[Group=2]*[Speed=3]	2.422	1.7813	1
	[Group=2]*[Speed=4]	3.755	2.4426	1
	[Group=2]*[Speed=5]	3.275	2.1701	1
[Group=2]*[Speed=2]	[Group=1]*[Speed=1]	-.424	2.7966	1
	[Group=1]*[Speed=2]	-.165	2.6933	1
	[Group=1]*[Speed=3]	.836	2.3848	1
	[Group=1]*[Speed=4]	2.925	2.4979	1
	[Group=1]*[Speed=5]	2.653	2.3365	1
	[Group=2]*[Speed=1]	-1.422	2.7591	1
	[Group=2]*[Speed=3]	1.000	2.3432	1
	[Group=2]*[Speed=4]	2.333	2.3605	1
	[Group=2]*[Speed=5]	1.853	2.3202	1
[Group=2]*[Speed=3]	[Group=1]*[Speed=1]	-1.424	1.7849	1
	[Group=1]*[Speed=2]	-1.165	1.6183	1
	[Group=1]*[Speed=3]	-.164	1.0258	1
	[Group=1]*[Speed=4]	1.925	1.2667	1
	[Group=1]*[Speed=5]	1.653	.9079	1
	[Group=2]*[Speed=1]	-2.422	1.7813	1
	[Group=2]*[Speed=2]	-1.000	2.3432	1
	[Group=2]*[Speed=4]	1.333	.8514	1
	[Group=2]*[Speed=5]	.853	.6507	1
[Group=2]*[Speed=4]	[Group=1]*[Speed=1]	-2.757	1.9131	1
	[Group=1]*[Speed=2]	-2.499	1.7586	1
	[Group=1]*[Speed=3]	-1.497	1.2355	1
	[Group=1]*[Speed=4]	.592	1.4417	1

Pairwise Comparisons

(I) Group*Speed	(J) Group*Speed	Bonferroni Sig.	95% Wald Confidence Interval for Difference	
			Lower	Upper
[Group=2]*[Speed=1]	[Group=1]*[Speed=1]	1.000	-7.163	9.160
	[Group=1]*[Speed=2]	1.000	-6.527	9.040
	[Group=1]*[Speed=3]	1.000	-4.370	8.886
	[Group=1]*[Speed=4]	1.000	-2.710	11.404
	[Group=1]*[Speed=5]	1.000	-2.367	10.518
	[Group=2]*[Speed=2]	1.000	-7.575	10.419
	[Group=2]*[Speed=3]	1.000	-3.386	8.231
	[Group=2]*[Speed=4]	1.000	-4.209	11.720
	[Group=2]*[Speed=5]	1.000	-3.801	10.352
[Group=2]*[Speed=2]	[Group=1]*[Speed=1]	1.000	-9.543	8.695
	[Group=1]*[Speed=2]	1.000	-8.948	8.617
	[Group=1]*[Speed=3]	1.000	-6.940	8.612
	[Group=1]*[Speed=4]	1.000	-5.220	11.070
	[Group=1]*[Speed=5]	1.000	-4.965	10.272
	[Group=2]*[Speed=1]	1.000	-10.419	7.575
	[Group=2]*[Speed=3]	1.000	-6.640	8.640
	[Group=2]*[Speed=4]	1.000	-5.364	10.030
	[Group=2]*[Speed=5]	1.000	-5.712	9.419
[Group=2]*[Speed=3]	[Group=1]*[Speed=1]	1.000	-7.244	4.396
	[Group=1]*[Speed=2]	1.000	-6.442	4.111
	[Group=1]*[Speed=3]	1.000	-3.509	3.181
	[Group=1]*[Speed=4]	1.000	-2.205	6.055
	[Group=1]*[Speed=5]	1.000	-1.307	4.614
	[Group=2]*[Speed=1]	1.000	-8.231	3.386
	[Group=2]*[Speed=2]	1.000	-8.640	6.640
	[Group=2]*[Speed=4]	1.000	-1.443	4.109
	[Group=2]*[Speed=5]	1.000	-1.269	2.975
[Group=2]*[Speed=4]	[Group=1]*[Speed=1]	1.000	-8.995	3.481
	[Group=1]*[Speed=2]	1.000	-8.233	3.236
	[Group=1]*[Speed=3]	1.000	-5.526	2.531
	[Group=1]*[Speed=4]	1.000	-4.109	5.293

Pairwise Comparisons

(I) Group*Speed	(J) Group*Speed	Mean Difference (I-J)	Std. Error	df
	[Group=1]*[Speed=5]	.320	1.1395	1
	[Group=2]*[Speed=1]	-3.755	2.4426	1
	[Group=2]*[Speed=2]	-2.333	2.3605	1
	[Group=2]*[Speed=3]	-1.333	.8514	1
	[Group=2]*[Speed=5]	-.480	.6843	1
[Group=2]*[Speed=5]	[Group=1]*[Speed=1]	-2.277	1.7421	1
	[Group=1]*[Speed=2]	-2.019	1.5708	1
	[Group=1]*[Speed=3]	-1.017	.9492	1
	[Group=1]*[Speed=4]	1.072	1.2055	1
	[Group=1]*[Speed=5]	.800	.8204	1
	[Group=2]*[Speed=1]	-3.275	2.1701	1
	[Group=2]*[Speed=2]	-1.853	2.3202	1
	[Group=2]*[Speed=3]	-.853	.6507	1
	[Group=2]*[Speed=4]	.480	.6843	1

Pairwise Comparisons

(I) Group*Speed	(J) Group*Speed	Bonferroni Sig.	95% Wald Confidence Interval for Difference	
			Lower	Upper
	[Group=1]*[Speed=5]	1.000	-3.395	4.035
	[Group=2]*[Speed=1]	1.000	-11.720	4.209
	[Group=2]*[Speed=2]	1.000	-10.030	5.364
	[Group=2]*[Speed=3]	1.000	-4.109	1.443
	[Group=2]*[Speed=5]	1.000	-2.711	1.751
[Group=2]*[Speed=5]	[Group=1]*[Speed=1]	1.000	-7.958	3.403
	[Group=1]*[Speed=2]	1.000	-7.141	3.103
	[Group=1]*[Speed=3]	1.000	-4.112	2.078
	[Group=1]*[Speed=4]	1.000	-2.859	5.003
	[Group=1]*[Speed=5]	1.000	-1.875	3.475
	[Group=2]*[Speed=1]	1.000	-10.352	3.801
	[Group=2]*[Speed=2]	1.000	-9.419	5.712
	[Group=2]*[Speed=3]	1.000	-2.975	1.269
	[Group=2]*[Speed=4]	1.000	-1.751	2.711

Pairwise comparisons of estimated marginal means based on the original scale of dependent variable Phi (°)

Overall Test Results

Wald Chi-Square	df	Sig.
13.050	9	.160

The Wald chi-square tests the effect of Group*Speed. This test is based on the linearly independent pairwise comparisons among the estimated marginal means.

```
GENLIN Accuracy BY Group Speed (ORDER=ASCENDING)
 /MODEL Group Speed Group*Speed INTERCEPT=YES
DISTRIBUTION=NORMAL LINK=IDENTITY
```

```
  /CRITERIA SCALE=MLE PCONVERGE=1E-006(ABSOLUTE) SINGULAR=1E-012 ANALYSISTY
PE=3(WALD) CILEVEL=95
    LIKELIHOOD=FULL
  /EMMEANS TABLES=Group SCALE=ORIGINAL COMPARE=Group CONTRAST=PAIRWISE PADJ
UST=BONFERRONI
  /EMMEANS TABLES=Speed SCALE=ORIGINAL COMPARE=Speed CONTRAST=PAIRWISE PADJ
UST=BONFERRONI
  /EMMEANS TABLES=Group*Speed SCALE=ORIGINAL COMPARE=Group CONTRAST=PAIRWIS
E
    PADJUST=BONFERRONI
      /EMMEANS TABLES=Group*Speed SCALE=ORIGINAL COMPARE=Speed CONTRAST=PAI
RWISE
    PADJUST=BONFERRONI
  /REPEATED SUBJECT=Subject SORT=YES CORRTYPE=INDEPENDENT ADJUSTCORR=YES CO
VB=ROBUST
  /MISSING CLASSMISSING=EXCLUDE
  /PRINT CPS DESCRIPTIVES MODELINFO FIT SUMMARY SOLUTION.
```

Generalized Linear Models

Notes

Output Created		12-MAY-2023 12:20:46
Comments		
Input	Data	C:\Users\andre\Documents\Andre\Pesquisa\Artigos para Publicar\Henrique Bianchi Mestrado\Statistics\Statistics Sheets\Statistics_Sheet.sav
	Active Dataset	DataSet1
	Filter	<none>
	Weight	<none>
	Split File	<none>
	N of Rows in Working Data File	68

Notes

Missing Value Handling	Definition of Missing	User-defined missing values for factor, subject and within-subject variables are treated as missing.
	Cases Used	Statistics are based on cases with valid data for all variables in the model.
Weight Handling		not applicable

Supplementary Material 2
Statistical
Output Sumamry SPSS software Title: Pendular mechanism determinants and elastic energy usage during walking of obese and non-obese children Authors: Peyré-Tartaruga et al. This documents includes all statistical procedures: 1. GEE for comparison
2. Pearson's correlation test

Notes

Syntax	GENLIN Accuracy BY Group Speed (ORDER=ASCENDING)<br>/MODEL Group Speed Group*Speed INTERCEPT=YES<br><br>DISTRIBUTION=NORMAL LINK=IDENTITY<br>/CRITERIA SCALE=MLE PCONVERGE=1E-006 (ABSOLUTE) SINGULAR=1E-012 ANALYSISTYPE=3 (WALD) CILEVEL=95<br>LIKELIHOOD=FULL<br>/EMMEANS TABLES=Group SCALE=ORIGINAL COMPARE=Group CONTRAST=PAIRWISE PADJUST=BONFERRONI<br>/EMMEANS TABLES=Speed SCALE=ORIGINAL COMPARE=Speed CONTRAST=PAIRWISE PADJUST=BONFERRONI<br>/EMMEANS TABLES=Group*Speed SCALE=ORIGINAL COMPARE=Group CONTRAST=PAIRWISE<br><br>PADJUST=BONFERRONI<br>/EMMEANS TABLES=Group*Speed SCALE=ORIGINAL COMPARE=Speed CONTRAST=PAIRWISE<br><br>PADJUST=BONFERRONI<br>/REPEATED SUBJECT=Subject SORT=YES CORRTYPE=INDEPENDENT ADJUSTCORR=YES COVB=ROBUST<br>/MISSING CLASSMISSING=EXCLUDE<br>/PRINT CPS DESCRIPTIVES MODELINFO FIT SUMMARY SOLUTION.

Notes

Resources	Processor Time	00:00:00.05
	Elapsed Time	00:00:00.06

Model Information

Dependent Variable		Accuracy (°)
Probability Distribution		Normal
Link Function		Identity
Subject Effect	1	Subject
Working Correlation Matrix Structure		Independent

Case Processing Summary

	N	Percent
Included	68	100.0%
Excluded	0	0.0%
Total	68	100.0%

Correlated Data Summary

Number of Levels	Subject Effect	Subject	17
Number of Subjects			17
Number of Measurements per Subject	Minimum		2
	Maximum		5
Correlation Matrix Dimension			5

Supplementary Material 2
Statistical
Output Sumamry SPSS software Title: Pendular mechanism determinants and elastic energy usage during walking of obese and non-obese children Authors: Peyré-Tartaruga et al. This documents includes all statistical procedures: 1. GEE for comparison
2. Pearson's correlation test

Categorical Variable Information

			N	Percent
Factor	Group	EUT	36	52.9%
		OB	32	47.1%
		Total	68	100.0%
	Speed	1	16	23.5%
		2	14	20.6%
		3	13	19.1%
		4	12	17.6%
		5	13	19.1%
		Total	68	100.0%

Continuous Variable Information

		N	Minimum	Maximum	Mean	Std. Deviation
Dependent Variable	Accuracy (°)	68	.1	5.7	1.798	1.5138

Goodness of Fit[a]

	Value
Quasi Likelihood under Independence Model Criterion (QIC)[b]	108.588
Corrected Quasi Likelihood under Independence Model Criterion (QICC)[b]	109.804

Dependent Variable: Accuracy (°)
Model: (Intercept), Group, Speed, Group * Speed[a]

a. Information criteria are in smaller-is-better form.

b. Computed using the full log quasi-likelihood function.

Tests of Model Effects

Source	Type III		
	Wald Chi-Square	df	Sig.
(Intercept)	226.935	1	.000
Group	.321	1	.571
Speed	103.521	4	.000
Group * Speed	6.742	4	.150

Dependent Variable: Accuracy (°)
Model: (Intercept), Group, Speed, Group * Speed

Parameter Estimates

Parameter	B	Std. Error	95% Wald Confidence Interval		Hypothesis Test
			Lower	Upper	Wald Chi-Square
(Intercept)	.560	.1951	.178	.942	8.235
[Group=1]	.325	.2619	-.188	.838	1.540
[Group=2]	0[a]	.	.	.	.
[Speed=1]	2.845	.5494	1.768	3.922	26.815
[Speed=2]	2.357	.7129	.959	3.754	10.927
[Speed=3]	.340	.3311	-.309	.989	1.054
[Speed=4]	.523	.2455	.042	1.004	4.544
[Speed=5]	0[a]	.	.	.	.
[Group=1] * [Speed=1]	-1.198	.7328	-2.635	.238	2.675
[Group=1] * [Speed=2]	-.894	.9091	-2.676	.888	.967
[Group=1] * [Speed=3]	-.122	.4638	-1.031	.787	.069
[Group=1] * [Speed=4]	-.053	.4287	-.894	.787	.015
[Group=1] * [Speed=5]	0[a]	.	.	.	.
[Group=2] * [Speed=1]	0[a]	.	.	.	.
[Group=2] * [Speed=2]	0[a]	.	.	.	.
[Group=2] * [Speed=3]	0[a]	.	.	.	.
[Group=2] * [Speed=4]	0[a]	.	.	.	.
[Group=2] * [Speed=5]	0[a]	.	.	.	.
(Scale)	1.548				

Parameter Estimates

Parameter	Hypothesis Test df	Sig.
(Intercept)	1	.004
[Group=1]	1	.215
[Group=2]	.	.
[Speed=1]	1	.000
[Speed=2]	1	.001
[Speed=3]	1	.305
[Speed=4]	1	.033
[Speed=5]	.	.
[Group=1] * [Speed=1]	1	.102
[Group=1] * [Speed=2]	1	.325
[Group=1] * [Speed=3]	1	.792
[Group=1] * [Speed=4]	1	.901
[Group=1] * [Speed=5]	.	.
[Group=2] * [Speed=1]	.	.
[Group=2] * [Speed=2]	.	.
[Group=2] * [Speed=3]	.	.
[Group=2] * [Speed=4]	.	.
[Group=2] * [Speed=5]	.	.
(Scale)		

Dependent Variable: Accuracy (°)
Model: (Intercept), Group, Speed, Group * Speed

a. Set to zero because this parameter is redundant.

Estimated Marginal Means 1: Group

Estimates

Group	Mean	Std. Error	95% Wald Confidence Interval Lower	Upper
EUT	1.644	.1760	1.299	1.989
OB	1.773	.1431	1.493	2.053

Pairwise Comparisons

(I) Group	(J) Group	Mean Difference (I-J)	Std. Error	df	Bonferroni Sig.	95% Wald Confidence ... Lower	95% Wald Confidence ... Upper
EUT	OB	-.129	.2268	1	.571	-.573	.316
OB	EUT	.129	.2268	1	.571	-.316	.573

Pairwise comparisons of estimated marginal means based on the original scale of dependent variable Accuracy (°)

Overall Test Results

Wald Chi-Square	df	Sig.
.321	1	.571

The Wald chi-square tests the effect of Group. This test is based on the linearly independent pairwise comparisons among the estimated marginal means.

Estimated Marginal Means 2: Speed

Estimates

Speed	Mean	Std. Error	95% Wald Confidence Interval Lower	95% Wald Confidence Interval Upper
1	2.968	.3858	2.212	3.724
2	2.632	.4172	1.814	3.450
3	1.001	.2428	.526	1.477
4	1.219	.2016	.824	1.614
5	.723	.1309	.466	.979

Pairwise Comparisons

(I) Speed	(J) Speed	Mean Difference (I-J)	Std. Error	df	Bonferroni Sig.	95% Wald Confidence ...
						Lower
1	2	.336	.7059	1	1.000	-1.645
	3	1.967[a]	.3297	1	.000	1.041
	4	1.749[a]	.4230	1	.000	.562
	5	2.246[a]	.3664	1	.000	1.217
2	1	-.336	.7059	1	1.000	-2.317
	3	1.631[a]	.5793	1	.049	.005
	4	1.413[a]	.4724	1	.028	.087
	5	1.910[a]	.4545	1	.000	.634
3	1	-1.967[a]	.3297	1	.000	-2.892
	2	-1.631[a]	.5793	1	.049	-3.257
	4	-.218	.3022	1	1.000	-1.066
	5	.279	.2319	1	1.000	-.372
4	1	-1.749[a]	.4230	1	.000	-2.936
	2	-1.413[a]	.4724	1	.028	-2.739
	3	.218	.3022	1	1.000	-.631
	5	.497	.2143	1	.205	-.105
5	1	-2.246[a]	.3664	1	.000	-3.274
	2	-1.910[a]	.4545	1	.000	-3.185
	3	-.279	.2319	1	1.000	-.930
	4	-.497	.2143	1	.205	-1.098

Pairwise Comparisons

(I) Speed	(J) Speed	95% Wald Confidence ... Upper
1	2	2.317
	3	2.892
	4	2.936
	5	3.274
2	1	1.645
	3	3.257
	4	2.739
	5	3.185
3	1	-1.041
	2	-.005
	4	.631
	5	.930
4	1	-.562
	2	-.087
	3	1.066
	5	1.098
5	1	-1.217
	2	-.634
	3	.372
	4	.105

Pairwise comparisons of estimated marginal means based on the original scale of dependent variable Accuracy (°)

a. The mean difference is significant at the .05 level.

Overall Test Results

Wald Chi-Square	df	Sig.
103.521	4	.000

The Wald chi-square tests the effect of Speed. This test is based on the linearly independent pairwise comparisons among the estimated marginal means.

Estimated Marginal Means 3: Group* Speed

Estimates

Group	Speed	Mean	Std. Error	95% Wald Confidence Interval Lower	Upper
EUT	1	2.531	.5483	1.457	3.606
	2	2.348	.5043	1.359	3.336
	3	1.103	.3692	.379	1.827
	4	1.355	.2721	.822	1.888
	5	.885	.1747	.543	1.227
OB	1	3.405	.5428	2.341	4.469
	2	2.917	.6648	1.614	4.220
	3	.900	.3153	.282	1.518
	4	1.083	.2976	.500	1.667
	5	.560	.1951	.178	.942

Pairwise Comparisons

Speed	(I) Group	(J) Group	Mean Difference (I-J)	Std. Error	df	Bonferroni Sig.
1	EUT	OB	-.873	.7716	1	.258
	OB	EUT	.873	.7716	1	.258
2	EUT	OB	-.569	.8344	1	.495
	OB	EUT	.569	.8344	1	.495
3	EUT	OB	.203	.4856	1	.676
	OB	EUT	-.203	.4856	1	.676
4	EUT	OB	.272	.4033	1	.501
	OB	EUT	-.272	.4033	1	.501
5	EUT	OB	.325	.2619	1	.215
	OB	EUT	-.325	.2619	1	.215

Pairwise Comparisons

Speed	(I) Group	(J) Group	95% Wald Confidence Interval for Difference	
			Lower	Upper
1	EUT	OB	-2.386	.639
	OB	EUT	-.639	2.386
2	EUT	OB	-2.205	1.066
	OB	EUT	-1.066	2.205
3	EUT	OB	-.749	1.155
	OB	EUT	-1.155	.749
4	EUT	OB	-.519	1.062
	OB	EUT	-1.062	.519
5	EUT	OB	-.188	.838
	OB	EUT	-.838	.188

Pairwise comparisons of estimated marginal means based on the original scale of dependent variable Accuracy (°)

Overall Test Results

Speed	Wald Chi-Square	df	Sig.
1	1.282	1	.258
2	.465	1	.495
3	.175	1	.676
4	.454	1	.501
5	1.540	1	.215

Each Wald chi-square tests the simple effects of Group within each level combination of the other factors shown. These tests are based on the linearly independent pairwise comparisons among the estimated marginal means.

Estimated Marginal Means 4: Group* Speed

Estimates

Group	Speed	Mean	Std. Error	95% Wald Confidence Interval	
				Lower	Upper
EUT	1	2.531	.5483	1.457	3.606
	2	2.348	.5043	1.359	3.336
	3	1.103	.3692	.379	1.827
	4	1.355	.2721	.822	1.888
	5	.885	.1747	.543	1.227
OB	1	3.405	.5428	2.341	4.469
	2	2.917	.6648	1.614	4.220
	3	.900	.3153	.282	1.518
	4	1.083	.2976	.500	1.667
	5	.560	.1951	.178	.942

Pairwise Comparisons

Group	(I) Speed	(J) Speed	Mean Difference (I-J)	Std. Error	df	Bonferroni Sig.
EUT	1	2	.184	.8579	1	1.000
		3	1.429[a]	.4664	1	.022
		4	1.176	.4711	1	.125
		5	1.646[a]	.4849	1	.007
	2	1	-.184	.8579	1	1.000
		3	1.245	.8232	1	1.000
		4	.993	.6690	1	1.000
		5	1.463	.5641	1	.095
	3	1	-1.429[a]	.4664	1	.022
		2	-1.245	.8232	1	1.000
		4	-.252	.3489	1	1.000
		5	.218	.3248	1	1.000
	4	1	-1.176	.4711	1	.125
		2	-.993	.6690	1	1.000
		3	.252	.3489	1	1.000
		5	.470	.3514	1	1.000
	5	1	-1.646[a]	.4849	1	.007
		2	-1.463	.5641	1	.095
		3	-.218	.3248	1	1.000
		4	-.470	.3514	1	1.000
OB	1	2	.488	1.1211	1	1.000
		3	2.505[a]	.4662	1	.000
		4	2.322[a]	.7026	1	.010
		5	2.845[a]	.5494	1	.000
	2	1	-.488	1.1211	1	1.000
		3	2.017	.8153	1	.134
		4	1.833	.6673	1	.060
		5	2.357[a]	.7129	1	.009
	3	1	-2.505[a]	.4662	1	.000
		2	-2.017	.8153	1	.134

Pairwise Comparisons

Group	(I) Speed	(J) Speed	95% Wald Confidence Interval for Difference Lower	Upper
EUT	1	2	-2.224	2.592
		3	.119	2.738
		4	-.146	2.499
		5	.285	3.008
	2	1	-2.592	2.224
		3	-1.066	3.555
		4	-.885	2.870
		5	-.121	3.046
	3	1	-2.738	-.119
		2	-3.555	1.066
		4	-1.232	.727
		5	-.694	1.129
	4	1	-2.499	.146
		2	-2.870	.885
		3	-.727	1.232
		5	-.516	1.456
	5	1	-3.008	-.285
		2	-3.046	.121
		3	-1.129	.694
		4	-1.456	.516
OB	1	2	-2.659	3.635
		3	1.196	3.814
		4	.349	4.294
		5	1.303	4.387
	2	1	-3.635	2.659
		3	-.272	4.305
		4	-.040	3.706
		5	.355	4.358
	3	1	-3.814	-1.196
		2	-4.305	.272

Pairwise Comparisons

Group	(I) Speed	(J) Speed	Mean Difference (I-J)	Std. Error	df	Bonferroni Sig.
		4	-.183	.4935	1	1.000
		5	.340	.3311	1	1.000
	4	1	-2.322[a]	.7026	1	.010
		2	-1.833	.6673	1	.060
		3	.183	.4935	1	1.000
		5	.523	.2455	1	.330
	5	1	-2.845[a]	.5494	1	.000
		2	-2.357[a]	.7129	1	.009
		3	-.340	.3311	1	1.000
		4	-.523	.2455	1	.330

Pairwise Comparisons

Group	(I) Speed	(J) Speed	95% Wald Confidence Interval for Difference	
			Lower	Upper
		4	-1.569	1.202
		5	-.590	1.270
	4	1	-4.294	-.349
		2	-3.706	.040
		3	-1.202	1.569
		5	-.166	1.212
	5	1	-4.387	-1.303
		2	-4.358	-.355
		3	-1.270	.590
		4	-1.212	.166

Pairwise comparisons of estimated marginal means based on the original scale of dependent variable Accuracy (°)

a. The mean difference is significant at the .05 level.

Overall Test Results

Group	Wald Chi-Square	df	Sig.
EUT	39.880	4	.000
OB	116.458	4	.000

Each Wald chi-square tests the simple effects of Speed within each level combination of the other factors shown. These tests are based on the linearly independent pairwise comparisons among the estimated marginal means.

```
 GENLIN PCI BY Group Speed (ORDER=ASCENDING)
  /MODEL Group Speed Group*Speed INTERCEPT=YES
 DISTRIBUTION=NORMAL LINK=IDENTITY
  /CRITERIA SCALE=MLE PCONVERGE=1E-006(ABSOLUTE) SINGULAR=1E-012 ANALYSISTY
PE=3(WALD) CILEVEL=95
    LIKELIHOOD=FULL
  /EMMEANS TABLES=Group SCALE=ORIGINAL COMPARE=Group CONTRAST=PAIRWISE PADJ
UST=BONFERRONI
  /EMMEANS TABLES=Speed SCALE=ORIGINAL COMPARE=Speed CONTRAST=PAIRWISE PADJ
UST=BONFERRONI
  /EMMEANS TABLES=Group*Speed SCALE=ORIGINAL COMPARE=Group CONTRAST=PAIRWIS
E
    PADJUST=BONFERRONI
      /EMMEANS TABLES=Group*Speed SCALE=ORIGINAL COMPARE=Speed CONTRAST=PAI
RWISE
    PADJUST=BONFERRONI
  /REPEATED SUBJECT=Subject SORT=YES CORRTYPE=INDEPENDENT ADJUSTCORR=YES CO
VB=ROBUST
  /MISSING CLASSMISSING=EXCLUDE
  /PRINT CPS DESCRIPTIVES MODELINFO FIT SUMMARY SOLUTION.
```

Generalized Linear Models

Notes

Output Created		12-MAY-2023 12:20:46
Comments		
Input	Data	C:\Users\andre\Documents\Andre\Pesquisa\Artigos para Publicar\Henrique Bianchi Mestrado\Statistics\Statistics Sheets\Statistics_Sheet.sav
	Active Dataset	DataSet1
	Filter	<none>
	Weight	<none>
	Split File	<none>
	N of Rows in Working Data File	68
Missing Value Handling	Definition of Missing	User-defined missing values for factor, subject and within-subject variables are treated as missing.
	Cases Used	Statistics are based on cases with valid data for all variables in the model.
Weight Handling		not applicable

Supplementary Material 2
Statistical
Output Sumamry SPSS software Title: Pendular mechanism determinants and elastic energy usage during walking of obese and non-obese children Authors: Peyré-Tartaruga et al. This documents includes all statistical procedures: 1. GEE for comparison
2. Pearson's correlation test

Notes

Syntax	GENLIN PCI BY Group Speed (ORDER=ASCENDING)<br>/MODEL Group Speed Group*Speed INTERCEPT=YES<br>DISTRIBUTION=NORMAL LINK=IDENTITY<br>/CRITERIA SCALE=MLE PCONVERGE=1E-006 (ABSOLUTE) SINGULAR=1E-012 ANALYSISTYPE=3 (WALD) CILEVEL=95<br>LIKELIHOOD=FULL<br>/EMMEANS TABLES=Group SCALE=ORIGINAL COMPARE=Group CONTRAST=PAIRWISE PADJUST=BONFERRONI<br>/EMMEANS TABLES=Speed SCALE=ORIGINAL COMPARE=Speed CONTRAST=PAIRWISE PADJUST=BONFERRONI<br>/EMMEANS TABLES=Group*Speed SCALE=ORIGINAL COMPARE=Group CONTRAST=PAIRWISE<br>PADJUST=BONFERRONI<br>/EMMEANS TABLES=Group*Speed SCALE=ORIGINAL COMPARE=Speed CONTRAST=PAIRWISE<br>PADJUST=BONFERRONI<br>/REPEATED SUBJECT=Subject SORT=YES CORRTYPE=INDEPENDENT ADJUSTCORR=YES COVB=ROBUST<br>/MISSING CLASSMISSING=EXCLUDE<br>/PRINT CPS DESCRIPTIVES MODELINFO FIT SUMMARY SOLUTION.

Notes

Resources	Processor Time	00:00:00.08
	Elapsed Time	00:00:00.08

Model Information

Dependent Variable		PCI (°)
Probability Distribution		Normal
Link Function		Identity
Subject Effect	1	Subject
Working Correlation Matrix Structure		Independent

Case Processing Summary

	N	Percent
Included	68	100.0%
Excluded	0	0.0%
Total	68	100.0%

Correlated Data Summary

Number of Levels	Subject Effect	Subject	17
Number of Subjects			17
Number of Measurements per Subject	Minimum		2
	Maximum		5
Correlation Matrix Dimension			5

Supplementary Material 2
Statistical
Output Sumamry SPSS software Title: Pendular mechanism determinants and elastic energy usage during walking of obese and non-obese children Authors: Peyré-Tartaruga et al. This documents includes all statistical procedures: 1. GEE for comparison 2. Pearson's correlation test

Categorical Variable Information

			N	Percent
Factor	Group	EUT	36	52.9%
		OB	32	47.1%
		Total	68	100.0%
	Speed	1	16	23.5%
		2	14	20.6%
		3	13	19.1%
		4	12	17.6%
		5	13	19.1%
		Total	68	100.0%

Continuous Variable Information

		N	Minimum	Maximum	Mean	Std. Deviation
Dependent Variable	PCI (°)	68	1.2	18.8	5.470	3.8941

Goodness of Fit[a]

	Value
Quasi Likelihood under Independence Model Criterion (QIC)[b]	482.241
Corrected Quasi Likelihood under Independence Model Criterion (QICC)[b]	484.215

Dependent Variable: PCI (°)
Model: (Intercept), Group, Speed, Group * Speed[a]

a. Information criteria are in smaller-is-better form.

b. Computed using the full log quasi-likelihood function.

Tests of Model Effects

Source	Type III		
	Wald Chi-Square	df	Sig.
(Intercept)	206.524	1	.000
Group	1.131	1	.287
Speed	78.208	4	.000
Group * Speed	4.201	4	.379

Dependent Variable: PCI (°)
Model: (Intercept), Group, Speed, Group * Speed

Parameter Estimates

Parameter	B	Std. Error	95% Wald Confidence Interval		Hypothesis Test
			Lower	Upper	Wald Chi-Square
(Intercept)	1.920	.2644	1.402	2.438	52.723
[Group=1]	.800	.4485	-.079	1.679	3.182
[Group=2]	0[a]	.	.	.	.
[Speed=1]	7.032	1.3694	4.348	9.716	26.373
[Speed=2]	4.130	.9219	2.323	5.937	20.069
[Speed=3]	1.663	.3543	.969	2.358	22.040
[Speed=4]	1.597	.6033	.414	2.779	7.005
[Speed=5]	0[a]	.	.	.	.
[Group=1] * [Speed=1]	.420	2.0825	-3.661	4.502	.041
[Group=1] * [Speed=2]	1.380	1.4898	-1.540	4.300	.858
[Group=1] * [Speed=3]	-.763	.8556	-2.440	.914	.796
[Group=1] * [Speed=4]	-1.197	.7729	-2.711	.318	2.397
[Group=1] * [Speed=5]	0[a]	.	.	.	.
[Group=2] * [Speed=1]	0[a]	.	.	.	.
[Group=2] * [Speed=2]	0[a]	.	.	.	.
[Group=2] * [Speed=3]	0[a]	.	.	.	.
[Group=2] * [Speed=4]	0[a]	.	.	.	.
[Group=2] * [Speed=5]	0[a]	.	.	.	.
(Scale)	8.004				

Parameter Estimates

Parameter	Hypothesis Test df	Sig.
(Intercept)	1	.000
[Group=1]	1	.074
[Group=2]	.	.
[Speed=1]	1	.000
[Speed=2]	1	.000
[Speed=3]	1	.000
[Speed=4]	1	.008
[Speed=5]	.	.
[Group=1] * [Speed=1]	1	.840
[Group=1] * [Speed=2]	1	.354
[Group=1] * [Speed=3]	1	.372
[Group=1] * [Speed=4]	1	.122
[Group=1] * [Speed=5]	.	.
[Group=2] * [Speed=1]	.	.
[Group=2] * [Speed=2]	.	.
[Group=2] * [Speed=3]	.	.
[Group=2] * [Speed=4]	.	.
[Group=2] * [Speed=5]	.	.
(Scale)		

Dependent Variable: PCI (°)
Model: (Intercept), Group, Speed, Group * Speed

a. Set to zero because this parameter is redundant.

Estimated Marginal Means 1: Group

Estimates

Group	Mean	Std. Error	95% Wald Confidence Interval Lower	Upper
EUT	5.573	.6656	4.268	6.877
OB	4.804	.2799	4.256	5.353

Pairwise Comparisons

(I) Group	(J) Group	Mean Difference (I-J)	Std. Error	df	Bonferroni Sig.	95% Wald Confidence ... Lower	95% Wald Confidence ... Upper
EUT	OB	.768	.7221	1	.287	-.647	2.183
OB	EUT	-.768	.7221	1	.287	-2.183	.647

Pairwise comparisons of estimated marginal means based on the original scale of dependent variable PCI (°)

Overall Test Results

Wald Chi-Square	df	Sig.
1.131	1	.287

The Wald chi-square tests the effect of Group. This test is based on the linearly independent pairwise comparisons among the estimated marginal means.

Estimated Marginal Means 2: Speed

Estimates

Speed	Mean	Std. Error	95% Wald Confidence Interval Lower	95% Wald Confidence Interval Upper
1	9.563	1.0919	7.423	11.703
2	7.140	.7578	5.655	8.625
3	3.602	.3515	2.913	4.291
4	3.318	.3662	2.601	4.036
5	2.320	.2242	1.880	2.760

Pairwise Comparisons

(I) Speed	(J) Speed	Mean Difference (I-J)	Std. Error	df	Bonferroni Sig.	95% Wald Confidence ... Lower
1	2	2.423	.9584	1	.115	-.268
	3	5.961[a]	1.1273	1	.000	2.797
	4	6.244[a]	1.0599	1	.000	3.269
	5	7.243[a]	1.0412	1	.000	4.320
2	1	-2.423	.9584	1	.115	-5.113
	3	3.538[a]	.9154	1	.001	.969
	4	3.822[a]	.8044	1	.000	1.564
	5	4.820[a]	.7449	1	.000	2.729
3	1	-5.961[a]	1.1273	1	.000	-9.125
	2	-3.538[a]	.9154	1	.001	-6.108
	4	.283	.5867	1	1.000	-1.364
	5	1.282[a]	.4278	1	.027	.081
4	1	-6.244[a]	1.0599	1	.000	-9.219
	2	-3.822[a]	.8044	1	.000	-6.080
	3	-.283	.5867	1	1.000	-1.930
	5	.998	.3864	1	.098	-.086
5	1	-7.243[a]	1.0412	1	.000	-10.165
	2	-4.820[a]	.7449	1	.000	-6.911
	3	-1.282[a]	.4278	1	.027	-2.483
	4	-.998	.3864	1	.098	-2.083

Pairwise Comparisons

(I) Speed	(J) Speed	95% Wald Confidence ... Upper
1	2	5.113
	3	9.125
	4	9.219
	5	10.165
2	1	.268
	3	6.108
	4	6.080
	5	6.911
3	1	-2.797
	2	-.969
	4	1.930
	5	2.483
4	1	-3.269
	2	-1.564
	3	1.364
	5	2.083
5	1	-4.320
	2	-2.729
	3	-.081
	4	.086

Pairwise comparisons of estimated marginal means based on the original scale of dependent variable PCI (°)

a. The mean difference is significant at the .05 level.

Overall Test Results

Wald Chi-Square	df	Sig.
78.208	4	.000

The Wald chi-square tests the effect of Speed. This test is based on the linearly independent pairwise comparisons among the estimated marginal means.

Estimated Marginal Means 3: Group* Speed

Estimates

Group	Speed	Mean	Std. Error	95% Wald Confidence Interval Lower	95% Wald Confidence Interval Upper
EUT	1	10.173	1.8025	6.640	13.706
	2	8.230	1.2919	5.698	10.762
	3	3.620	.6348	2.376	4.864
	4	3.120	.4336	2.270	3.970
	5	2.720	.3622	2.010	3.430
OB	1	8.952	1.2328	6.536	11.369
	2	6.050	.7926	4.497	7.603
	3	3.583	.3022	2.991	4.176
	4	3.517	.5904	2.360	4.674
	5	1.920	.2644	1.402	2.438

Pairwise Comparisons

Speed	(I) Group	(J) Group	Mean Difference (I-J)	Std. Error	df	Bonferroni Sig.
1	EUT	OB	1.220	2.1837	1	.576
	OB	EUT	-1.220	2.1837	1	.576
2	EUT	OB	2.180	1.5156	1	.150
	OB	EUT	-2.180	1.5156	1	.150
3	EUT	OB	.037	.7031	1	.958
	OB	EUT	-.037	.7031	1	.958
4	EUT	OB	-.397	.7325	1	.588
	OB	EUT	.397	.7325	1	.588
5	EUT	OB	.800	.4485	1	.074
	OB	EUT	-.800	.4485	1	.074

Pairwise Comparisons

Speed	(I) Group	(J) Group	95% Wald Confidence Interval for Difference	
			Lower	Upper
1	EUT	OB	-3.060	5.500
	OB	EUT	-5.500	3.060
2	EUT	OB	-.791	5.151
	OB	EUT	-5.151	.791
3	EUT	OB	-1.341	1.415
	OB	EUT	-1.415	1.341
4	EUT	OB	-1.832	1.039
	OB	EUT	-1.039	1.832
5	EUT	OB	-.079	1.679
	OB	EUT	-1.679	.079

Pairwise comparisons of estimated marginal means based on the original scale of dependent variable PCI (°)

Overall Test Results

Speed	Wald Chi-Square	df	Sig.
1	.312	1	.576
2	2.069	1	.150
3	.003	1	.958
4	.293	1	.588
5	3.182	1	.074

Each Wald chi-square tests the simple effects of Group within each level combination of the other factors shown. These tests are based on the linearly independent pairwise comparisons among the estimated marginal means.

Estimated Marginal Means 4: Group* Speed

Estimates

Group	Speed	Mean	Std. Error	95% Wald Confidence Interval	
				Lower	Upper
EUT	1	10.173	1.8025	6.640	13.706
	2	8.230	1.2919	5.698	10.762
	3	3.620	.6348	2.376	4.864
	4	3.120	.4336	2.270	3.970
	5	2.720	.3622	2.010	3.430
OB	1	8.952	1.2328	6.536	11.369
	2	6.050	.7926	4.497	7.603
	3	3.583	.3022	2.991	4.176
	4	3.517	.5904	2.360	4.674
	5	1.920	.2644	1.402	2.438

Pairwise Comparisons

Group	(I) Speed	(J) Speed	Mean Difference (I-J)	Std. Error	df	Bonferroni Sig.
EUT	1	2	1.943	1.2371	1	1.000
		3	6.553[a]	1.9168	1	.006
		4	7.053[a]	1.5638	1	.000
		5	7.453[a]	1.5689	1	.000
	2	1	-1.943	1.2371	1	1.000
		3	4.610[a]	1.5724	1	.034
		4	5.110[a]	1.2626	1	.001
		5	5.510[a]	1.1703	1	.000
	3	1	-6.553[a]	1.9168	1	.006
		2	-4.610[a]	1.5724	1	.034
		4	.500	.8579	1	1.000
		5	.900	.7788	1	1.000
	4	1	-7.053[a]	1.5638	1	.000
		2	-5.110[a]	1.2626	1	.001
		3	-.500	.8579	1	1.000
		5	.400	.4831	1	1.000
	5	1	-7.453[a]	1.5689	1	.000
		2	-5.510[a]	1.1703	1	.000
		3	-.900	.7788	1	1.000
		4	-.400	.4831	1	1.000
OB	1	2	2.902	1.4642	1	.475
		3	5.369[a]	1.1872	1	.000
		4	5.436[a]	1.4311	1	.001
		5	7.032[a]	1.3694	1	.000
	2	1	-2.902	1.4642	1	.475
		3	2.467	.9375	1	.085
		4	2.533	.9970	1	.111
		5	4.130[a]	.9219	1	.000

Pairwise Comparisons

Group	(I) Speed	(J) Speed	95% Wald Confidence Interval for Difference	
			Lower	Upper
EUT	1	2	-1.530	5.415
		3	1.172	11.933
		4	2.663	11.443
		5	3.049	11.857
	2	1	-5.415	1.530
		3	.196	9.024
		4	1.566	8.654
		5	2.225	8.795
	3	1	-11.933	-1.172
		2	-9.024	-.196
		4	-1.908	2.908
		5	-1.286	3.086
	4	1	-11.443	-2.663
		2	-8.654	-1.566
		3	-2.908	1.908
		5	-.956	1.756
	5	1	-11.857	-3.049
		2	-8.795	-2.225
		3	-3.086	1.286
		4	-1.756	.956
OB	1	2	-1.208	7.013
		3	2.037	8.702
		4	1.419	9.453
		5	3.188	10.876
	2	1	-7.013	1.208
		3	-.165	5.098
		4	-.265	5.332
		5	1.542	6.718

Pairwise Comparisons

Group	(I) Speed	(J) Speed	Mean Difference (I-J)	Std. Error	df	Bonferroni Sig.
	3	1	-5.369[a]	1.1872	1	.000
		2	-2.467	.9375	1	.085
		4	.067	.8006	1	1.000
		5	1.663[a]	.3543	1	.000
	4	1	-5.436[a]	1.4311	1	.001
		2	-2.533	.9970	1	.111
		3	-.067	.8006	1	1.000
		5	1.597	.6033	1	.081
	5	1	-7.032[a]	1.3694	1	.000
		2	-4.130[a]	.9219	1	.000
		3	-1.663[a]	.3543	1	.000
		4	-1.597	.6033	1	.081

Pairwise Comparisons

Group	(I) Speed	(J) Speed	95% Wald Confidence Interval for Difference	
			Lower	Upper
	3	1	-8.702	-2.037
		2	-5.098	.165
		4	-2.181	2.314
		5	.669	2.658
	4	1	-9.453	-1.419
		2	-5.332	.265
		3	-2.314	2.181
		5	-.097	3.290
	5	1	-10.876	-3.188
		2	-6.718	-1.542
		3	-2.658	-.669
		4	-3.290	.097

Pairwise comparisons of estimated marginal means based on the original scale of dependent variable PCI (°)

a. The mean difference is significant at the .05 level.

Overall Test Results

Group	Wald Chi-Square	df	Sig.
EUT	35.295	4	.000
OB	51.000	4	.000

Each Wald chi-square tests the simple effects of Speed within each level combination of the other factors shown. These tests are based on the linearly independent pairwise comparisons among the estimated marginal means.

```
GENLIN Variability BY Group Speed (ORDER=ASCENDING)
  /MODEL Group Speed Group*Speed INTERCEPT=YES
 DISTRIBUTION=NORMAL LINK=IDENTITY
  /CRITERIA SCALE=MLE PCONVERGE=1E-006(ABSOLUTE) SINGULAR=1E-012 ANALYSISTY
PE=3(WALD) CILEVEL=95
    LIKELIHOOD=FULL
  /EMMEANS TABLES=Group SCALE=ORIGINAL COMPARE=Group CONTRAST=PAIRWISE PADJ
UST=BONFERRONI
  /EMMEANS TABLES=Speed SCALE=ORIGINAL COMPARE=Speed CONTRAST=PAIRWISE PADJ
UST=BONFERRONI
  /EMMEANS TABLES=Group*Speed SCALE=ORIGINAL COMPARE=Group CONTRAST=PAIRWIS
E
    PADJUST=BONFERRONI
      /EMMEANS TABLES=Group*Speed SCALE=ORIGINAL COMPARE=Speed CONTRAST=PAI
RWISE
    PADJUST=BONFERRONI
  /REPEATED SUBJECT=Subject SORT=YES CORRTYPE=INDEPENDENT ADJUSTCORR=YES CO
VB=ROBUST
  /MISSING CLASSMISSING=EXCLUDE
  /PRINT CPS DESCRIPTIVES MODELINFO FIT SUMMARY SOLUTION.
```

Generalized Linear Models

Notes

Output Created		13-MAY-2023 12:07:05
Comments		
Input	Data	C:\Users\andre\Documents\Andre\Pesquisa\Artigos para Publicar\Henrique Bianchi Mestrado\Statistics\Statistics Sheets\Statistics_Sheet.sav
	Active Dataset	DataSet1
	Filter	<none>
	Weight	<none>
	Split File	<none>
	N of Rows in Working Data File	68
Missing Value Handling	Definition of Missing	User-defined missing values for factor, subject and within-subject variables are treated as missing.
	Cases Used	Statistics are based on cases with valid data for all variables in the model.
Weight Handling		not applicable

Supplementary Material 2
Statistical
Output Sumamry SPSS software Title: Pendular mechanism determinants and elastic energy usage during walking of obese and non-obese children Authors: Peyré-Tartaruga et al. This documents includes all statistical procedures: 1. GEE for comparison
2. Pearson's correlation test

Notes

Syntax	GENLIN Variability BY Group Speed (ORDER=ASCENDING)<br>/MODEL Group Speed Group*Speed INTERCEPT=YES<br><br>DISTRIBUTION=NORMAL LINK=IDENTITY<br>/CRITERIA SCALE=MLE PCONVERGE=1E-006 (ABSOLUTE) SINGULAR=1E-012 ANALYSISTYPE=3 (WALD) CILEVEL=95<br>LIKELIHOOD=FULL<br>/EMMEANS TABLES=Group SCALE=ORIGINAL COMPARE=Group CONTRAST=PAIRWISE PADJUST=BONFERRONI<br>/EMMEANS TABLES=Speed SCALE=ORIGINAL COMPARE=Speed CONTRAST=PAIRWISE PADJUST=BONFERRONI<br>/EMMEANS TABLES=Group*Speed SCALE=ORIGINAL COMPARE=Group CONTRAST=PAIRWISE<br><br>PADJUST=BONFERRONI<br>/EMMEANS TABLES=Group*Speed SCALE=ORIGINAL COMPARE=Speed CONTRAST=PAIRWISE<br><br>PADJUST=BONFERRONI<br>/REPEATED SUBJECT=Subject SORT=YES CORRTYPE=INDEPENDENT ADJUSTCORR=YES COVB=ROBUST<br>/MISSING CLASSMISSING=EXCLUDE<br>/PRINT CPS DESCRIPTIVES MODELINFO FIT SUMMARY SOLUTION.

Notes

Resources	Processor Time	00:00:00.13
	Elapsed Time	00:00:00.09

```
[DataSet1] C:\Users\andre\Documents\Andre\Pesquisa\Artigos para Publicar\He
nrique Bianchi Mestrado\Statistics\Statistics Sheets\Statistics_Sheet.sav
```

Model Information

Dependent Variable		Variability (°)
Probability Distribution		Normal
Link Function		Identity
Subject Effect	1	Subject
Working Correlation Matrix Structure		Independent

Case Processing Summary

	N	Percent
Included	67	98.5%
Excluded	1	1.5%
Total	68	100.0%

Correlated Data Summary

Number of Levels	Subject Effect	Subject	17
Number of Subjects			17
Number of Measurements per Subject	Minimum		1
	Maximum		5
Correlation Matrix Dimension			5

Supplementary Material 2
Statistical
Output Sumamry SPSS software Title: Pendular mechanism determinants and elastic energy usage during walking of obese and non-obese children Authors: Peyré-Tartaruga et al. This documents includes all statistical procedures: 1. GEE for comparison 2. Pearson's correlation test

Categorical Variable Information

			N	Percent
Factor	Group	EUT	36	53.7%
		OB	31	46.3%
		Total	67	100.0%
	Speed	1	15	22.4%
		2	14	20.9%
		3	13	19.4%
		4	12	17.9%
		5	13	19.4%
		Total	67	100.0%

Continuous Variable Information

		N	Minimum	Maximum	Mean	Std. Deviation
Dependent Variable	Variability (°)	67	.1	13.3	3.731	3.0005

Goodness of Fit[a]

	Value
Quasi Likelihood under Independence Model Criterion (QIC)[b]	310.312
Corrected Quasi Likelihood under Independence Model Criterion (QICC)[b]	312.503

Dependent Variable: Variability (°)
Model: (Intercept), Group, Speed, Group * Speed[a]

a. Information criteria are in smaller-is-better form.

b. Computed using the full log quasi-likelihood function.

Tests of Model Effects

Source	Type III		
	Wald Chi-Square	df	Sig.
(Intercept)	142.780	1	.000
Group	1.574	1	.210
Speed	62.933	4	.000
Group * Speed	10.704	4	.030

Dependent Variable: Variability (°)
Model: (Intercept), Group, Speed, Group * Speed

Parameter Estimates

Parameter	B	Std. Error	95% Wald Confidence Interval		Hypothesis Test
			Lower	Upper	Wald Chi-Square
(Intercept)	1.360	.0829	1.197	1.523	268.837
[Group=1]	.474	.2680	-.052	.999	3.124
[Group=2]	0[a]	.	.	.	.
[Speed=1]	4.856	1.0677	2.763	6.949	20.684
[Speed=2]	1.790	.4199	.967	2.613	18.177
[Speed=3]	1.357	.1473	1.068	1.645	84.773
[Speed=4]	1.107	.3791	.364	1.850	8.520
[Speed=5]	0[a]	.	.	.	.
[Group=1] * [Speed=1]	.952	1.5539	-2.094	3.997	.375
[Group=1] * [Speed=2]	2.260	1.2858	-.260	4.780	3.089
[Group=1] * [Speed=3]	-.672	.7615	-2.164	.821	.778
[Group=1] * [Speed=4]	-1.175	.4388	-2.035	-.315	7.175
[Group=1] * [Speed=5]	0[a]	.	.	.	.
[Group=2] * [Speed=1]	0[a]	.	.	.	.
[Group=2] * [Speed=2]	0[a]	.	.	.	.
[Group=2] * [Speed=3]	0[a]	.	.	.	.
[Group=2] * [Speed=4]	0[a]	.	.	.	.
[Group=2] * [Speed=5]	0[a]	.	.	.	.
(Scale)	5.132				

Parameter Estimates

Parameter	Hypothesis Test df	Sig.
(Intercept)	1	.000
[Group=1]	1	.077
[Group=2]	.	.
[Speed=1]	1	.000
[Speed=2]	1	.000
[Speed=3]	1	.000
[Speed=4]	1	.004
[Speed=5]	.	.
[Group=1] * [Speed=1]	1	.540
[Group=1] * [Speed=2]	1	.079
[Group=1] * [Speed=3]	1	.378
[Group=1] * [Speed=4]	1	.007
[Group=1] * [Speed=5]	.	.
[Group=2] * [Speed=1]	.	.
[Group=2] * [Speed=2]	.	.
[Group=2] * [Speed=3]	.	.
[Group=2] * [Speed=4]	.	.
[Group=2] * [Speed=5]	.	.
(Scale)		

Dependent Variable: Variability (°)
Model: (Intercept), Group, Speed, Group * Speed

a. Set to zero because this parameter is redundant.

Estimated Marginal Means 1: Group

Estimates

Group	Mean	Std. Error	95% Wald Confidence Interval Lower	Upper
EUT	3.929	.5427	2.865	4.992
OB	3.182	.2441	2.703	3.660

Pairwise Comparisons

(I) Group	(J) Group	Mean Difference (I-J)	Std. Error	df	Bonferroni Sig.	95% Wald Confidence ... Lower	95% Wald Confidence ... Upper
EUT	OB	.747	.5951	1	.210	-.420	1.913
OB	EUT	-.747	.5951	1	.210	-1.913	.420

Pairwise comparisons of estimated marginal means based on the original scale of dependent variable Variability (°)

Overall Test Results

Wald Chi-Square	df	Sig.
1.574	1	.210

The Wald chi-square tests the effect of Group. This test is based on the linearly independent pairwise comparisons among the estimated marginal means.

Estimated Marginal Means 2: Speed

Estimates

Speed	Mean	Std. Error	95% Wald Confidence Interval Lower	95% Wald Confidence Interval Upper
1	6.929	.8148	5.332	8.526
2	4.517	.6896	3.165	5.869
3	2.618	.3089	2.012	3.223
4	2.116	.2193	1.686	2.546
5	1.597	.1340	1.334	1.860

Pairwise Comparisons

(I) Speed	(J) Speed	Mean Difference (I-J)	Std. Error	df	Bonferroni Sig.	95% Wald Confidence ...
						Lower
1	2	2.412[a]	.6204	1	.001	.670
	3	4.311[a]	.9168	1	.000	1.737
	4	4.813[a]	.7602	1	.000	2.679
	5	5.332[a]	.7769	1	.000	3.151
2	1	-2.412[a]	.6204	1	.001	-4.153
	3	1.899	.7662	1	.132	-.252
	4	2.401[a]	.6944	1	.005	.452
	5	2.920[a]	.6429	1	.000	1.115
3	1	-4.311[a]	.9168	1	.000	-6.885
	2	-1.899	.7662	1	.132	-4.050
	4	.502	.4567	1	1.000	-.780
	5	1.021	.3808	1	.073	-.048
4	1	-4.813[a]	.7602	1	.000	-6.947
	2	-2.401[a]	.6944	1	.005	-4.350
	3	-.502	.4567	1	1.000	-1.784
	5	.519	.2194	1	.180	-.097
5	1	-5.332[a]	.7769	1	.000	-7.513
	2	-2.920[a]	.6429	1	.000	-4.725
	3	-1.021	.3808	1	.073	-2.090
	4	-.519	.2194	1	.180	-1.135

Pairwise Comparisons

(I) Speed	(J) Speed	95% Wald Confidence ... Upper
1	2	4.153
	3	6.885
	4	6.947
	5	7.513
2	1	-.670
	3	4.050
	4	4.350
	5	4.725
3	1	-1.737
	2	.252
	4	1.784
	5	2.090
4	1	-2.679
	2	-.452
	3	.780
	5	1.135
5	1	-3.151
	2	-1.115
	3	.048
	4	.097

Pairwise comparisons of estimated marginal means based on the original scale of dependent variable Variability (°)

a. The mean difference is significant at the .05 level.

Overall Test Results

Wald Chi-Square	df	Sig.
62.933	4	.000

The Wald chi-square tests the effect of Speed. This test is based on the linearly independent pairwise comparisons among the estimated marginal means.

Estimated Marginal Means 3: Group* Speed

Estimates

Group	Speed	Mean	Std. Error	95% Wald Confidence Interval Lower	Upper
EUT	1	7.641	1.2761	5.140	10.143
	2	5.884	1.3181	3.300	8.467
	3	2.519	.6112	1.321	3.716
	4	1.765	.2351	1.304	2.226
	5	1.834	.2549	1.334	2.333
OB	1	6.216	1.0134	4.230	8.202
	2	3.150	.4060	2.354	3.946
	3	2.717	.0895	2.541	2.892
	4	2.467	.3702	1.741	3.192
	5	1.360	.0829	1.197	1.523

Pairwise Comparisons

Speed	(I) Group	(J) Group	Mean Difference (I-J)	Std. Error	df	Bonferroni Sig.
1	EUT	OB	1.426	1.6296	1	.382
	OB	EUT	-1.426	1.6296	1	.382
2	EUT	OB	2.734[a]	1.3793	1	.047
	OB	EUT	-2.734[a]	1.3793	1	.047
3	EUT	OB	-.198	.6177	1	.748
	OB	EUT	.198	.6177	1	.748
4	EUT	OB	-.702	.4386	1	.110
	OB	EUT	.702	.4386	1	.110
5	EUT	OB	.474	.2680	1	.077
	OB	EUT	-.474	.2680	1	.077

Pairwise Comparisons

Speed	(I) Group	(J) Group	95% Wald Confidence Interval for Difference	
			Lower	Upper
1	EUT	OB	-1.768	4.619
	OB	EUT	-4.619	1.768
2	EUT	OB	.030	5.437
	OB	EUT	-5.437	-.030
3	EUT	OB	-1.409	1.013
	OB	EUT	-1.013	1.409
4	EUT	OB	-1.561	.158
	OB	EUT	-.158	1.561
5	EUT	OB	-.052	.999
	OB	EUT	-.999	.052

Pairwise comparisons of estimated marginal means based on the original scale of dependent variable Variability (°)

a. The mean difference is significant at the .05 level.

Overall Test Results

Speed	Wald Chi-Square	df	Sig.
1	.765	1	.382
2	3.928	1	.047
3	.103	1	.748
4	2.560	1	.110
5	3.124	1	.077

Each Wald chi-square tests the simple effects of Group within each level combination of the other factors shown. These tests are based on the linearly independent pairwise comparisons among the estimated marginal means.

Estimated Marginal Means 4: Group* Speed

Estimates

Group	Speed	Mean	Std. Error	95% Wald Confidence Interval	
				Lower	Upper
EUT	1	7.641	1.2761	5.140	10.143
	2	5.884	1.3181	3.300	8.467
	3	2.519	.6112	1.321	3.716
	4	1.765	.2351	1.304	2.226
	5	1.834	.2549	1.334	2.333
OB	1	6.216	1.0134	4.230	8.202
	2	3.150	.4060	2.354	3.946
	3	2.717	.0895	2.541	2.892
	4	2.467	.3702	1.741	3.192
	5	1.360	.0829	1.197	1.523

Pairwise Comparisons

Group	(I) Speed	(J) Speed	Mean Difference (I-J)	Std. Error	df	Bonferroni Sig.
EUT	1	2	1.758[a]	.6087	1	.039
		3	5.123[a]	1.5450	1	.009
		4	5.876[a]	1.1589	1	.000
		5	5.808[a]	1.1289	1	.000
	2	1	-1.758[a]	.6087	1	.039
		3	3.365	1.4704	1	.221
		4	4.119[a]	1.2833	1	.013
		5	4.050[a]	1.2154	1	.009
	3	1	-5.123[a]	1.5450	1	.009
		2	-3.365	1.4704	1	.221
		4	.754	.8172	1	1.000
		5	.685	.7471	1	1.000
	4	1	-5.876[a]	1.1589	1	.000
		2	-4.119[a]	1.2833	1	.013
		3	-.754	.8172	1	1.000
		5	-.069	.2209	1	1.000
	5	1	-5.808[a]	1.1289	1	.000
		2	-4.050[a]	1.2154	1	.009
		3	-.685	.7471	1	1.000
		4	.069	.2209	1	1.000
OB	1	2	3.066[a]	1.0812	1	.046
		3	3.499[a]	.9875	1	.004
		4	3.749[a]	.9842	1	.001
		5	4.856[a]	1.0677	1	.000
	2	1	-3.066[a]	1.0812	1	.046
		3	.433	.4315	1	1.000
		4	.683	.5313	1	1.000
		5	1.790[a]	.4199	1	.000

Pairwise Comparisons

Group	(I) Speed	(J) Speed	95% Wald Confidence Interval for Difference	
			Lower	Upper
EUT	1	2	.049	3.466
		3	.786	9.460
		4	2.623	9.130
		5	2.639	8.977
	2	1	-3.466	-.049
		3	-.762	7.493
		4	.517	7.721
		5	.638	7.462
	3	1	-9.460	-.786
		2	-7.493	.762
		4	-1.540	3.047
		5	-1.412	2.782
	4	1	-9.130	-2.623
		2	-7.721	-.517
		3	-3.047	1.540
		5	-.689	.551
	5	1	-8.977	-2.639
		2	-7.462	-.638
		3	-2.782	1.412
		4	-.551	.689
OB	1	2	.031	6.101
		3	.727	6.271
		4	.986	6.512
		5	1.859	7.853
	2	1	-6.101	-.031
		3	-.778	1.644
		4	-.808	2.175
		5	.611	2.969

Pairwise Comparisons

Group	(I) Speed	(J) Speed	Mean Difference (I-J)	Std. Error	df	Bonferroni Sig.
	3	1	-3.499[a]	.9875	1	.004
		2	-.433	.4315	1	1.000
		4	.250	.4081	1	1.000
		5	1.357[a]	.1473	1	.000
	4	1	-3.749[a]	.9842	1	.001
		2	-.683	.5313	1	1.000
		3	-.250	.4081	1	1.000
		5	1.107[a]	.3791	1	.035
	5	1	-4.856[a]	1.0677	1	.000
		2	-1.790[a]	.4199	1	.000
		3	-1.357[a]	.1473	1	.000
		4	-1.107[a]	.3791	1	.035

Pairwise Comparisons

Group	(I) Speed	(J) Speed	95% Wald Confidence Interval for Difference	
			Lower	Upper
	3	1	-6.271	-.727
		2	-1.644	.778
		4	-.895	1.395
		5	.943	1.770
	4	1	-6.512	-.986
		2	-2.175	.808
		3	-1.395	.895
		5	.042	2.171
	5	1	-7.853	-1.859
		2	-2.969	-.611
		3	-1.770	-.943
		4	-2.171	-.042

Supplementary Material 2
Statistical
Output Sumamry SPSS software Title: Pendular mechanism determinants and elastic energy usage during walking of obese and non-obese children Authors: Peyré-Tartaruga et al. This documents includes all statistical procedures: 1. GEE for comparison

Pairwise comparisons of estimated marginal means based on the original scale of dependent variable Variability (°)

a. The mean difference is significant at the .05 level.

Overall Test Results

Group	Wald Chi-Square	df	Sig.
EUT	50.786	4	.000
OB	113.850	4	.000

Each Wald chi-square tests the simple effects of Speed within each level combination of the other factors shown. These tests are based on the linearly independent pairwise comparisons among the estimated marginal means.

```
 GET
  FILE='C:\Users\andre\Documents\Andre\Pesquisa\Artigos para Publicar\Henri
que Bianchi Mestrado\Statistics\Statistics Sheets\Statistics_Sheet.sav'.
DATASET NAME DataSet1 WINDOW=FRONT.
GENLIN MPEEStored BY Group Speed (ORDER=ASCENDING)
  /MODEL Group Speed Group*Speed INTERCEPT=YES
 DISTRIBUTION=NORMAL LINK=IDENTITY
  /CRITERIA SCALE=MLE PCONVERGE=1E-006(ABSOLUTE) SINGULAR=1E-012 ANALYSISTY
PE=3(WALD) CILEVEL=95
    LIKELIHOOD=FULL
  /EMMEANS TABLES=Group SCALE=ORIGINAL COMPARE=Group CONTRAST=PAIRWISE PADJ
UST=BONFERRONI
  /EMMEANS TABLES=Speed SCALE=ORIGINAL COMPARE=Speed CONTRAST=PAIRWISE PADJ
UST=BONFERRONI
  /EMMEANS TABLES=Group*Speed SCALE=ORIGINAL COMPARE=Group CONTRAST=PAIRWIS
E
    PADJUST=BONFERRONI
      /EMMEANS TABLES=Group*Speed SCALE=ORIGINAL COMPARE=Speed CONTRAST=PAI
RWISE
    PADJUST=BONFERRONI
  /REPEATED SUBJECT=Subject SORT=YES CORRTYPE=INDEPENDENT ADJUSTCORR=YES CO
VB=ROBUST
```

```
/MISSING CLASSMISSING=EXCLUDE
/PRINT CPS DESCRIPTIVES MODELINFO FIT SUMMARY SOLUTION.
```

Generalized Linear Models

Notes

Output Created		13-MAY-2023 16:51:50
Comments		
Input	Data	C:\Users\andre\Documents\Andre\Pesquisa\Artigos para Publicar\Henrique Bianchi Mestrado\Statistics\Statistics Sheets\Statistics_Sheet.sav
	Active Dataset	DataSet1
	Filter	<none>
	Weight	<none>
	Split File	<none>
	N of Rows in Working Data File	68
Missing Value Handling	Definition of Missing	User-defined missing values for factor, subject and within-subject variables are treated as missing.
	Cases Used	Statistics are based on cases with valid data for all variables in the model.
Weight Handling		not applicable

Supplementary Material 2
Statistical
Output Sumamry SPSS software Title: Pendular mechanism determinants and elastic energy usage during walking of obese and non-obese children Authors: Peyré-Tartaruga et al. This documents includes all statistical procedures: 1. GEE for comparison
2. Pearson's correlation test

Notes

Syntax	GENLIN MPEEStored BY Group Speed (ORDER=ASCENDING)<br>/MODEL Group Speed Group*Speed INTERCEPT=YES<br>DISTRIBUTION=NORMAL LINK=IDENTITY<br>/CRITERIA SCALE=MLE PCONVERGE=1E-006 (ABSOLUTE) SINGULAR=1E-012 ANALYSISTYPE=3 (WALD) CILEVEL=95<br>LIKELIHOOD=FULL<br>/EMMEANS TABLES=Group SCALE=ORIGINAL COMPARE=Group CONTRAST=PAIRWISE PADJUST=BONFERRONI<br>/EMMEANS TABLES=Speed SCALE=ORIGINAL COMPARE=Speed CONTRAST=PAIRWISE PADJUST=BONFERRONI<br>/EMMEANS TABLES=Group*Speed SCALE=ORIGINAL COMPARE=Group CONTRAST=PAIRWISE<br>PADJUST=BONFERRONI<br>/EMMEANS TABLES=Group*Speed SCALE=ORIGINAL COMPARE=Speed CONTRAST=PAIRWISE<br>PADJUST=BONFERRONI<br>/REPEATED SUBJECT=Subject SORT=YES CORRTYPE=INDEPENDENT ADJUSTCORR=YES COVB=ROBUST<br>/MISSING CLASSMISSING=EXCLUDE<br>/PRINT CPS DESCRIPTIVES MODELINFO FIT SUMMARY SOLUTION.

Supplementary Material 2
Statistical
Output Sumamry SPSS software Title: Pendular mechanism determinants and elastic energy usage during walking of obese and non-obese children Authors: Peyré-Tartaruga et al. This documents includes all statistical procedures: 1. GEE for comparison
2. Pearson's correlation test

Notes

Resources	Processor Time	00:00:00.14
	Elapsed Time	00:00:00.16

```
[DataSet1] C:\Users\andre\Documents\Andre\Pesquisa\Artigos para Publicar\He
nrique Bianchi Mestrado\Statistics\Statistics Sheets\Statistics_Sheet.sav
```

Model Information

Dependent Variable		MPEE Stored (J/kg/m)
Probability Distribution		Normal
Link Function		Identity
Subject Effect	1	Subject
Working Correlation Matrix Structure		Independent

Case Processing Summary

	N	Percent
Included	68	100.0%
Excluded	0	0.0%
Total	68	100.0%

Correlated Data Summary

Number of Levels	Subject Effect	Subject	17
Number of Subjects			17
Number of Measurements per Subject	Minimum		2
	Maximum		5
Correlation Matrix Dimension			5

Supplementary Material 2
Statistical
Output Sumamry SPSS software Title: Pendular mechanism determinants and elastic energy usage during walking of obese and non-obese children Authors: Peyré-Tartaruga et al. This documents includes all statistical procedures: 1. GEE for comparison
2. Pearson's correlation test

Categorical Variable Information

			N	Percent
Factor	Group	EUT	36	52.9%
		OB	32	47.1%
		Total	68	100.0%
	Speed	1	16	23.5%
		2	14	20.6%
		3	13	19.1%
		4	12	17.6%
		5	13	19.1%
		Total	68	100.0%

Continuous Variable Information

		N	Minimum	Maximum	Mean
Dependent Variable	MPEE Stored (J/kg/m)	68	.055	.221	.13190

Continuous Variable Information

		Std. Deviation
Dependent Variable	MPEE Stored (J/kg/m)	.036215

Goodness of Fit[a]

	Value
Quasi Likelihood under Independence Model Criterion (QIC)[b]	18.987
Corrected Quasi Likelihood under Independence Model Criterion (QICC)[b]	20.080

Dependent Variable: MPEE Stored (J/kg/m)
Model: (Intercept), Group, Speed, Group * Speed[a]

a. Information criteria are in smaller-is-better form.

b. Computed using the full log quasi-likelihood function.

Tests of Model Effects

Source	Type III Wald Chi-Square	df	Sig.
(Intercept)	783.736	1	.000
Group	1.458	1	.227
Speed	1.821	4	.769
Group * Speed	3.669	4	.453

Dependent Variable: MPEE Stored (J/kg/m)
Model: (Intercept), Group, Speed, Group * Speed

Parameter Estimates

Parameter	B	Std. Error	95% Wald Confidence Interval Lower	Upper	Hypothesis Test Wald Chi-Square
(Intercept)	.115	.0144	.087	.143	63.424
[Group=1]	.025	.0206	-.015	.066	1.518
[Group=2]	0[a]	.	.	.	.
[Speed=1]	.005	.0198	-.034	.044	.066
[Speed=2]	.018	.0165	-.015	.050	1.168
[Speed=3]	.017	.0176	-.018	.051	.905
[Speed=4]	.015	.0178	-.020	.050	.681
[Speed=5]	0[a]	.	.	.	.
[Group=1] * [Speed=1]	.002	.0274	-.052	.055	.003
[Group=1] * [Speed=2]	-.011	.0266	-.063	.041	.160
[Group=1] * [Speed=3]	-.035	.0276	-.089	.020	1.569
[Group=1] * [Speed=4]	-.026	.0270	-.079	.027	.951
[Group=1] * [Speed=5]	0[a]	.	.	.	.
[Group=2] * [Speed=1]	0[a]	.	.	.	.
[Group=2] * [Speed=2]	0[a]	.	.	.	.
[Group=2] * [Speed=3]	0[a]	.	.	.	.
[Group=2] * [Speed=4]	0[a]	.	.	.	.
[Group=2] * [Speed=5]	0[a]	.	.	.	.
(Scale)	.001				

Parameter Estimates

	Hypothesis Test	
Parameter	df	Sig.
(Intercept)	1	.000
[Group=1]	1	.218
[Group=2]	.	.
[Speed=1]	1	.797
[Speed=2]	1	.280
[Speed=3]	1	.341
[Speed=4]	1	.409
[Speed=5]	.	.
[Group=1] * [Speed=1]	1	.956
[Group=1] * [Speed=2]	1	.690
[Group=1] * [Speed=3]	1	.210
[Group=1] * [Speed=4]	1	.329
[Group=1] * [Speed=5]	.	.
[Group=2] * [Speed=1]	.	.
[Group=2] * [Speed=2]	.	.
[Group=2] * [Speed=3]	.	.
[Group=2] * [Speed=4]	.	.
[Group=2] * [Speed=5]	.	.
(Scale)		

Dependent Variable: MPEE Stored (J/kg/m)
Model: (Intercept), Group, Speed, Group * Speed

a. Set to zero because this parameter is redundant.

Estimated Marginal Means 1: Group

Estimates

Group	Mean	Std. Error	95% Wald Confidence Interval Lower	Upper
EUT	.13700	.005930	.12538	.14862
OB	.12567	.007271	.11142	.13992

Pairwise Comparisons

(I) Group	(J) Group	Mean Difference (I-J)	Std. Error	df	Bonferroni Sig.	95% Wald Confidence ...	
						Lower	Upper
EUT	OB	.01133	.009383	1	.227	-.00706	.02972
OB	EUT	-.01133	.009383	1	.227	-.02972	.00706

Pairwise comparisons of estimated marginal means based on the original scale of dependent variable MPEE Stored (J/kg/m)

Overall Test Results

Wald Chi-Square	df	Sig.
1.458	1	.227

The Wald chi-square tests the effect of Group. This test is based on the linearly independent pairwise comparisons among the estimated marginal means.

Estimated Marginal Means 2: Speed

Estimates

Speed	Mean	Std. Error	95% Wald Confidence Interval	
			Lower	Upper
1	.13330	.010126	.11346	.15315
2	.14002	.008071	.12420	.15584
3	.12689	.008865	.10952	.14427
4	.12900	.006994	.11529	.14271
5	.12746	.010276	.10732	.14760

Pairwise Comparisons

(I) Speed	(J) Speed	Mean Difference (I-J)	Std. Error	df	Bonferroni Sig.	95% Wald Confidence ...
						Lower
1	2	-.00672	.011193	1	1.000	-.03814
	3	.00641	.010037	1	1.000	-.02177
	4	.00430	.011990	1	1.000	-.02935
	5	.00584	.013723	1	1.000	-.03268
2	1	.00672	.011193	1	1.000	-.02470
	3	.01313	.010886	1	1.000	-.01743
	4	.01102	.012111	1	1.000	-.02298
	5	.01256	.013287	1	1.000	-.02474
3	1	-.00641	.010037	1	1.000	-.03458
	2	-.01313	.010886	1	1.000	-.04369
	4	-.00211	.009023	1	1.000	-.02744
	5	-.00057	.013789	1	1.000	-.03927
4	1	-.00430	.011990	1	1.000	-.03796
	2	-.01102	.012111	1	1.000	-.04502
	3	.00211	.009023	1	1.000	-.02322
	5	.00154	.013497	1	1.000	-.03635
5	1	-.00584	.013723	1	1.000	-.04436
	2	-.01256	.013287	1	1.000	-.04985
	3	.00057	.013789	1	1.000	-.03814
	4	-.00154	.013497	1	1.000	-.03943

Pairwise Comparisons

(I) Speed	(J) Speed	95% Wald Confidence ... Upper
1	2	.02470
	3	.03458
	4	.03796
	5	.04436
2	1	.03814
	3	.04369
	4	.04502
	5	.04985
3	1	.02177
	2	.01743
	4	.02322
	5	.03814
4	1	.02935
	2	.02298
	3	.02744
	5	.03943
5	1	.03268
	2	.02474
	3	.03927
	4	.03635

Pairwise comparisons of estimated marginal means based on the original scale of dependent variable MPEE Stored (J/kg/m)

Overall Test Results

Wald Chi-Square	df	Sig.
1.821	4	.769

The Wald chi-square tests the effect of Speed. This test is based on the linearly independent pairwise comparisons among the estimated marginal means.

Estimated Marginal Means 3: Group* Speed

Estimates

Group	Speed	Mean	Std. Error	95% Wald Confidence Interval Lower	Upper
EUT	1	.14671	.013624	.12001	.17342
	2	.14737	.011422	.12499	.16976
	3	.12229	.014579	.09371	.15086
	4	.12850	.009760	.10937	.14763
	5	.14013	.014648	.11141	.16884
OB	1	.11989	.014983	.09052	.14926
	2	.13267	.011406	.11031	.15502
	3	.13150	.010088	.11173	.15127
	4	.12950	.010019	.10986	.14914
	5	.11480	.014415	.08655	.14305

Pairwise Comparisons

Speed	(I) Group	(J) Group	Mean Difference (I-J)	Std. Error	df	Bonferroni Sig.
1	EUT	OB	.02683	.020252	1	.185
	OB	EUT	-.02683	.020252	1	.185
2	EUT	OB	.01471	.016142	1	.362
	OB	EUT	-.01471	.016142	1	.362
3	EUT	OB	-.00921	.017729	1	.603
	OB	EUT	.00921	.017729	1	.603
4	EUT	OB	-.00100	.013987	1	.943
	OB	EUT	.00100	.013987	1	.943
5	EUT	OB	.02532	.020552	1	.218
	OB	EUT	-.02532	.020552	1	.218

Pairwise Comparisons

Speed	(I) Group	(J) Group	95% Wald Confidence Interval for Difference	
			Lower	Upper
1	EUT	OB	-.01287	.06652
	OB	EUT	-.06652	.01287
2	EUT	OB	-.01693	.04635
	OB	EUT	-.04635	.01693
3	EUT	OB	-.04396	.02553
	OB	EUT	-.02553	.04396
4	EUT	OB	-.02841	.02641
	OB	EUT	-.02641	.02841
5	EUT	OB	-.01496	.06561
	OB	EUT	-.06561	.01496

Pairwise comparisons of estimated marginal means based on the original scale of dependent variable MPEE Stored (J/kg/m)

Overall Test Results

Speed	Wald Chi-Square	df	Sig.
1	1.755	1	.185
2	.830	1	.362
3	.270	1	.603
4	.005	1	.943
5	1.518	1	.218

Each Wald chi-square tests the simple effects of Group within each level combination of the other factors shown. These tests are based on the linearly independent pairwise comparisons among the estimated marginal means.

Estimated Marginal Means 4: Group* Speed

Estimates

Group	Speed	Mean	Std. Error	95% Wald Confidence Interval	
				Lower	Upper
EUT	1	.14671	.013624	.12001	.17342
	2	.14737	.011422	.12499	.16976
	3	.12229	.014579	.09371	.15086
	4	.12850	.009760	.10937	.14763
	5	.14013	.014648	.11141	.16884
OB	1	.11989	.014983	.09052	.14926
	2	.13267	.011406	.11031	.15502
	3	.13150	.010088	.11173	.15127
	4	.12950	.010019	.10986	.14914
	5	.11480	.014415	.08655	.14305

Pairwise Comparisons

Group	(I) Speed	(J) Speed	Mean Difference (I-J)	Std. Error	df	Bonferroni Sig.
EUT	1	2	-.00066	.017353	1	1.000
		3	.02443	.013207	1	.644
		4	.01821	.018662	1	1.000
		5	.00659	.018985	1	1.000
	2	1	.00066	.017353	1	1.000
		3	.02509	.016495	1	1.000
		4	.01887	.017050	1	1.000
		5	.00725	.020804	1	1.000
	3	1	-.02443	.013207	1	.644
		2	-.02509	.016495	1	1.000
		4	-.00621	.016415	1	1.000
		5	-.01784	.021271	1	1.000
	4	1	-.01821	.018662	1	1.000
		2	-.01887	.017050	1	1.000
		3	.00621	.016415	1	1.000
		5	-.01162	.020288	1	1.000

Pairwise Comparisons

			95% Wald Confidence Interval for Difference	
Group	(I) Speed	(J) Speed	Lower	Upper
EUT	1	2	-.04937	.04805
		3	-.01264	.06150
		4	-.03417	.07060
		5	-.04670	.05988
	2	1	-.04805	.04937
		3	-.02121	.07139
		4	-.02899	.06674
		5	-.05115	.06565
	3	1	-.06150	.01264
		2	-.07139	.02121
		4	-.05229	.03986
		5	-.07755	.04187
	4	1	-.07060	.03417
		2	-.06674	.02899
		3	-.03986	.05229
		5	-.06857	.04532

Pairwise Comparisons

Group	(I) Speed	(J) Speed	Mean Difference (I-J)	Std. Error	df	Bonferroni Sig.
	5	1	-.00659	.018985	1	1.000
		2	-.00725	.020804	1	1.000
		3	.01784	.021271	1	1.000
		4	.01162	.020288	1	1.000
OB	1	2	-.01278	.014141	1	1.000
		3	-.01161	.015118	1	1.000
		4	-.00961	.015058	1	1.000
		5	.00509	.019821	1	1.000
	2	1	.01278	.014141	1	1.000
		3	.00117	.014211	1	1.000
		4	.00317	.017206	1	1.000
		5	.01787	.016533	1	1.000
	3	1	.01161	.015118	1	1.000
		2	-.00117	.014211	1	1.000
		4	.00200	.007498	1	1.000
		5	.01670	.017551	1	1.000
	4	1	.00961	.015058	1	1.000
		2	-.00317	.017206	1	1.000
		3	-.00200	.007498	1	1.000
		5	.01470	.017808	1	1.000
	5	1	-.00509	.019821	1	1.000
		2	-.01787	.016533	1	1.000
		3	-.01670	.017551	1	1.000
		4	-.01470	.017808	1	1.000

Pairwise Comparisons

Group	(I) Speed	(J) Speed	95% Wald Confidence Interval for Difference Lower	Upper
	5	1	-.05988	.04670
		2	-.06565	.05115
		3	-.04187	.07755
		4	-.04532	.06857
OB	1	2	-.05247	.02692
		3	-.05405	.03083
		4	-.05188	.03266
		5	-.05055	.06073
	2	1	-.02692	.05247
		3	-.03872	.04106
		4	-.04513	.05146
		5	-.02854	.06428
	3	1	-.03083	.05405
		2	-.04106	.03872
		4	-.01905	.02305
		5	-.03257	.06597
	4	1	-.03266	.05188
		2	-.05146	.04513
		3	-.02305	.01905
		5	-.03529	.06469
	5	1	-.06073	.05055
		2	-.06428	.02854
		3	-.06597	.03257
		4	-.06469	.03529

Pairwise comparisons of estimated marginal means based on the original scale of dependent variable MPEE Stored (J/kg/m)

Overall Test Results

Group	Wald Chi-Square	df	Sig.
EUT	4.622	4	.328
OB	2.461	4	.652

Each Wald chi-square tests the simple effects of Speed within each level combination of the other factors shown. These tests are based on the linearly independent pairwise comparisons among the estimated marginal means.

```
  GENLIN MPEEReleased BY Group Speed (ORDER=ASCENDING)
 /MODEL Group Speed Group*Speed INTERCEPT=YES
DISTRIBUTION=NORMAL LINK=IDENTITY
 /CRITERIA SCALE=MLE PCONVERGE=1E-006(ABSOLUTE) SINGULAR=1E-012 ANALYSISTY
PE=3(WALD) CILEVEL=95
    LIKELIHOOD=FULL
 /EMMEANS TABLES=Group SCALE=ORIGINAL COMPARE=Group CONTRAST=PAIRWISE PADJ
UST=BONFERRONI
 /EMMEANS TABLES=Speed SCALE=ORIGINAL COMPARE=Speed CONTRAST=PAIRWISE PADJ
UST=BONFERRONI
 /EMMEANS TABLES=Group*Speed SCALE=ORIGINAL COMPARE=Group CONTRAST=PAIRWIS
E
    PADJUST=BONFERRONI
      /EMMEANS TABLES=Group*Speed SCALE=ORIGINAL COMPARE=Speed CONTRAST=PAI
RWISE
    PADJUST=BONFERRONI
 /REPEATED SUBJECT=Subject SORT=YES CORRTYPE=INDEPENDENT ADJUSTCORR=YES CO
VB=ROBUST
 /MISSING CLASSMISSING=EXCLUDE
 /PRINT CPS DESCRIPTIVES MODELINFO FIT SUMMARY SOLUTION.
```

Generalized Linear Models

Supplementary Material 2
Statistical
Output Sumamry SPSS software Title: Pendular mechanism determinants and elastic energy usage during walking of obese and non-obese children Authors: Peyré-Tartaruga et al. This documents includes all statistical procedures: 1. GEE for comparison 2. Pearson's correlation test

Model Information

Dependent Variable	MPEE Released (J/kg/m)
Probability Distribution	Normal
Link Function	Identity
Subject Effect 1	Subject
Working Correlation Matrix Structure	Independent

Case Processing Summary

	N	Percent
Included	68	100.0%
Excluded	0	0.0%
Total	68	100.0%

Notes

Output Created		13-MAY-2023 16:51:50
Comments		
Input	Data	C: \Users\andre\Documents\Andre\Pesquisa\Artigos para Publicar\Henrique Bianchi Mestrado\Statistics\Statistics Sheets\Statistics_Sheet.sav
	Active Dataset	DataSet1
	Filter	<none>
	Weight	<none>
	Split File	<none>
	N of Rows in Working Data File	68

Notes

Missing Value Handling	Definition of Missing	User-defined missing values for factor, subject and within-subject variables are treated as missing.
	Cases Used	Statistics are based on cases with valid data for all variables in the model.
Weight Handling		not applicable

Supplementary Material 2
Statistical
Output Sumamry SPSS software Title: Pendular mechanism determinants and elastic energy usage during walking of obese and non-obese children Authors: Peyré-Tartaruga et al. This documents includes all statistical procedures: 1. GEE for comparison
2. Pearson's correlation test

Notes

Syntax	GENLIN MPEEReleased BY Group Speed (ORDER=ASCENDING) /MODEL Group Speed Group*Speed INTERCEPT=YES DISTRIBUTION=NORMAL LINK=IDENTITY /CRITERIA SCALE=MLE PCONVERGE=1E-006 (ABSOLUTE) SINGULAR=1E-012 ANALYSISTYPE=3 (WALD) CILEVEL=95 LIKELIHOOD=FULL /EMMEANS TABLES=Group SCALE=ORIGINAL COMPARE=Group CONTRAST=PAIRWISE PADJUST=BONFERRONI /EMMEANS TABLES=Speed SCALE=ORIGINAL COMPARE=Speed CONTRAST=PAIRWISE PADJUST=BONFERRONI /EMMEANS TABLES=Group*Speed SCALE=ORIGINAL COMPARE=Group CONTRAST=PAIRWISE PADJUST=BONFERRONI /EMMEANS TABLES=Group*Speed SCALE=ORIGINAL COMPARE=Speed CONTRAST=PAIRWISE PADJUST=BONFERRONI /REPEATED SUBJECT=Subject SORT=YES CORRTYPE=INDEPENDENT ADJUSTCORR=YES COVB=ROBUST /MISSING CLASSMISSING=EXCLUDE /PRINT CPS DESCRIPTIVES MODELINFO FIT SUMMARY SOLUTION.

Supplementary Material 2
Statistical
Output Sumamry SPSS software Title: Pendular mechanism determinants and elastic energy usage during walking of obese and non-obese children Authors: Peyré-Tartaruga et al. This documents includes all statistical procedures: 1. GEE for comparison
2. Pearson's correlation test

Notes

Resources	Processor Time	00:00:00.23
	Elapsed Time	00:00:00.19

Correlated Data Summary

Number of Levels	Subject Effect	Subject	17
Number of Subjects			17
Number of Measurements per Subject	Minimum		2
	Maximum		5
Correlation Matrix Dimension			5

Categorical Variable Information

			N	Percent
Factor	Group	EUT	36	52.9%
		OB	32	47.1%
		Total	68	100.0%
	Speed	1	16	23.5%
		2	14	20.6%
		3	13	19.1%
		4	12	17.6%
		5	13	19.1%
		Total	68	100.0%

Continuous Variable Information

		N	Minimum	Maximum	Mean
Dependent Variable	MPEE Released (J/kg/m)	68	.012	.167	.05282

Continuous Variable Information

		Std. Deviation
Dependent Variable	MPEE Released (J/kg/m)	.033302

Supplementary Material 2
Statistical
Output Sumamry SPSS software Title: Pendular mechanism determinants and elastic energy usage during walking of obese and non-obese children Authors: Peyré-Tartaruga et al. This documents includes all statistical procedures: 1. GEE for comparison
2. Pearson's correlation test

Goodness of Fit[a]

	Value
Quasi Likelihood under Independence Model Criterion (QIC)[b]	18.504
Corrected Quasi Likelihood under Independence Model Criterion (QICC)[b]	20.022

Dependent Variable: MPEE Released (J/kg/m)
Model: (Intercept), Group, Speed, Group * Speed[a]

a. Information criteria are in smaller-is-better form.

b. Computed using the full log quasi-likelihood function.

Tests of Model Effects

Source	Type III		
	Wald Chi-Square	df	Sig.
(Intercept)	381.638	1	.000
Group	26.826	1	.000
Speed	88.266	4	.000
Group * Speed	9.190	4	.057

Dependent Variable: MPEE Released (J/kg/m)
Model: (Intercept), Group, Speed, Group * Speed

Parameter Estimates

Parameter	B	Std. Error	95% Wald Confidence Interval		Hypothesis Test
			Lower	Upper	Wald Chi-Square
(Intercept)	.032	.0056	.021	.043	33.594
[Group=1]	-.010	.0068	-.023	.003	2.191
[Group=2]	0[a]	.	.	.	.
[Speed=1]	.076	.0123	.052	.100	38.599
[Speed=2]	.046	.0083	.030	.063	31.027
[Speed=3]	.018	.0068	.005	.032	7.166
[Speed=4]	.020	.0073	.006	.035	7.937
[Speed=5]	0[a]	.	.	.	.
[Group=1] * [Speed=1]	-.031	.0153	-.061	-.001	4.113
[Group=1] * [Speed=2]	-.026	.0103	-.046	-.005	6.209
[Group=1] * [Speed=3]	-.006	.0088	-.024	.011	.543
[Group=1] * [Speed=4]	-.022	.0088	-.039	-.005	6.304
[Group=1] * [Speed=5]	0[a]	.	.	.	.
[Group=2] * [Speed=1]	0[a]	.	.	.	.
[Group=2] * [Speed=2]	0[a]	.	.	.	.
[Group=2] * [Speed=3]	0[a]	.	.	.	.
[Group=2] * [Speed=4]	0[a]	.	.	.	.
[Group=2] * [Speed=5]	0[a]	.	.	.	.
(Scale)	.000				

Parameter Estimates

Parameter	Hypothesis Test	
	df	Sig.
(Intercept)	1	.000
[Group=1]	1	.139
[Group=2]	.	.
[Speed=1]	1	.000
[Speed=2]	1	.000
[Speed=3]	1	.007
[Speed=4]	1	.005
[Speed=5]	.	.
[Group=1] * [Speed=1]	1	.043
[Group=1] * [Speed=2]	1	.013
[Group=1] * [Speed=3]	1	.461
[Group=1] * [Speed=4]	1	.012
[Group=1] * [Speed=5]	.	.
[Group=2] * [Speed=1]	.	.
[Group=2] * [Speed=2]	.	.
[Group=2] * [Speed=3]	.	.
[Group=2] * [Speed=4]	.	.
[Group=2] * [Speed=5]	.	.
(Scale)		

Dependent Variable: MPEE Released (J/kg/m)
Model: (Intercept), Group, Speed, Group * Speed

a. Set to zero because this parameter is redundant.

Estimated Marginal Means 1: Group

Estimates

Group	Mean	Std. Error	95% Wald Confidence Interval	
			Lower	Upper
EUT	.03755	.003817	.03007	.04503
OB	.06465	.003578	.05763	.07166

Pairwise Comparisons

(I) Group	(J) Group	Mean Difference (I-J)	Std. Error	df	Bonferroni Sig.	95% Wald Confidence ... Lower	95% Wald Confidence ... Upper
EUT	OB	-.02710[a]	.005231	1	.000	-.03735	-.01684
OB	EUT	.02710[a]	.005231	1	.000	.01684	.03735

Pairwise comparisons of estimated marginal means based on the original scale of dependent variable MPEE Released (J/kg/m)

a. The mean difference is significant at the .05 level.

Overall Test Results

Wald Chi-Square	df	Sig.
26.826	1	.000

The Wald chi-square tests the effect of Group. This test is based on the linearly independent pairwise comparisons among the estimated marginal means.

Estimated Marginal Means 2: Speed

Estimates

Speed	Mean	Std. Error	95% Wald Confidence Interval Lower	95% Wald Confidence Interval Upper
1	.08812	.006002	.07636	.09988
2	.06083	.004733	.05156	.07011
3	.04240	.004808	.03298	.05183
4	.03675	.003249	.03038	.04312
5	.02739	.003386	.02075	.03402

Pairwise Comparisons

(I) Speed	(J) Speed	Mean Difference (I-J)	Std. Error	df	Bonferroni Sig.	95% Wald Confidence ... Lower
1	2	.02729[a]	.006632	1	.000	.00867
	3	.04571[a]	.007015	1	.000	.02602
	4	.05137[a]	.005918	1	.000	.03476
	5	.06073[a]	.007660	1	.000	.03923
2	1	-.02729[a]	.006632	1	.000	-.04590
	3	.01843[a]	.006515	1	.047	.00014
	4	.02408[a]	.005407	1	.000	.00890
	5	.03345[a]	.005145	1	.000	.01900
3	1	-.04571[a]	.007015	1	.000	-.06541
	2	-.01843[a]	.006515	1	.047	-.03672
	4	.00565	.004779	1	1.000	-.00776
	5	.01502[a]	.004409	1	.007	.00264
4	1	-.05137[a]	.005918	1	.000	-.06798
	2	-.02408[a]	.005407	1	.000	-.03926
	3	-.00565	.004779	1	1.000	-.01907
	5	.00936	.004409	1	.337	-.00301
5	1	-.06073[a]	.007660	1	.000	-.08223
	2	-.03345[a]	.005145	1	.000	-.04789
	3	-.01502[a]	.004409	1	.007	-.02739
	4	-.00936	.004409	1	.337	-.02174

Pairwise Comparisons

(I) Speed	(J) Speed	95% Wald Confidence ... Upper
1	2	.04590
	3	.06541
	4	.06798
	5	.08223
2	1	-.00867
	3	.03672
	4	.03926
	5	.04789
3	1	-.02602
	2	-.00014
	4	.01907
	5	.02739
4	1	-.03476
	2	-.00890
	3	.00776
	5	.02174
5	1	-.03923
	2	-.01900
	3	-.00264
	4	.00301

Pairwise comparisons of estimated marginal means based on the original scale of dependent variable MPEE Released (J/kg/m)

a. The mean difference is significant at the .05 level.

Overall Test Results

Wald Chi-Square	df	Sig.
88.266	4	.000

The Wald chi-square tests the effect of Speed. This test is based on the linearly independent pairwise comparisons among the estimated marginal means.

Estimated Marginal Means 3: Group* Speed

Estimates

Group	Speed	Mean	Std. Error	95% Wald Confidence Interval Lower	95% Wald Confidence Interval Upper
EUT	1	.06757	.008010	.05187	.08327
	2	.04300	.008544	.02625	.05975
	3	.03414	.004842	.02465	.04363
	4	.02067	.003186	.01442	.02691
	5	.02238	.003824	.01488	.02987
OB	1	.10867	.008940	.09114	.12619
	2	.07867	.004073	.07068	.08665
	3	.05067	.008309	.03438	.06695
	4	.05283	.005664	.04173	.06393
	5	.03240	.005590	.02144	.04336

Pairwise Comparisons

Speed	(I) Group	(J) Group	Mean Difference (I-J)	Std. Error	df	Bonferroni Sig.
1	EUT	OB	-.04110[a]	.012003	1	.001
	OB	EUT	.04110[a]	.012003	1	.001
2	EUT	OB	-.03567[a]	.009465	1	.000
	OB	EUT	.03567[a]	.009465	1	.000
3	EUT	OB	-.01652	.009617	1	.086
	OB	EUT	.01652	.009617	1	.086
4	EUT	OB	-.03217[a]	.006498	1	.000
	OB	EUT	.03217[a]	.006498	1	.000
5	EUT	OB	-.01002	.006773	1	.139
	OB	EUT	.01002	.006773	1	.139

Pairwise Comparisons

Speed	(I) Group	(J) Group	95% Wald Confidence Interval for Difference	
			Lower	Upper
1	EUT	OB	-.06462	-.01757
	OB	EUT	.01757	.06462
2	EUT	OB	-.05422	-.01711
	OB	EUT	.01711	.05422
3	EUT	OB	-.03537	.00232
	OB	EUT	-.00232	.03537
4	EUT	OB	-.04490	-.01943
	OB	EUT	.01943	.04490
5	EUT	OB	-.02330	.00325
	OB	EUT	-.00325	.02330

Pairwise comparisons of estimated marginal means based on the original scale of dependent variable MPEE Released (J/kg/m)

a. The mean difference is significant at the .05 level.

Overall Test Results

Speed	Wald Chi-Square	df	Sig.
1	11.721	1	.001
2	14.199	1	.000
3	2.952	1	.086
4	24.503	1	.000
5	2.191	1	.139

Each Wald chi-square tests the simple effects of Group within each level combination of the other factors shown. These tests are based on the linearly independent pairwise comparisons among the estimated marginal means.

Estimated Marginal Means 4: Group* Speed

Estimates

Group	Speed	Mean	Std. Error	95% Wald Confidence Interval	
				Lower	Upper
EUT	1	.06757	.008010	.05187	.08327
	2	.04300	.008544	.02625	.05975
	3	.03414	.004842	.02465	.04363
	4	.02067	.003186	.01442	.02691
	5	.02238	.003824	.01488	.02987
OB	1	.10867	.008940	.09114	.12619
	2	.07867	.004073	.07068	.08665
	3	.05067	.008309	.03438	.06695
	4	.05283	.005664	.04173	.06393
	5	.03240	.005590	.02144	.04336

Pairwise Comparisons

Group	(I) Speed	(J) Speed	Mean Difference (I-J)	Std. Error	df	Bonferroni Sig.
EUT	1	2	.02457	.009383	1	.088
		3	.03343[a]	.007741	1	.000
		4	.04690[a]	.007478	1	.000
		5	.04520[a]	.009165	1	.000
	2	1	-.02457	.009383	1	.088
		3	.00886	.009008	1	1.000
		4	.02233	.008368	1	.076
		5	.02062[a]	.006075	1	.007
	3	1	-.03343[a]	.007741	1	.000
		2	-.00886	.009008	1	1.000
		4	.01348	.005513	1	.145
		5	.01177	.005586	1	.351
	4	1	-.04690[a]	.007478	1	.000
		2	-.02233	.008368	1	.076
		3	-.01348	.005513	1	.145
		5	-.00171	.005016	1	1.000
	5	1	-.04520[a]	.009165	1	.000
		2	-.02062[a]	.006075	1	.007
		3	-.01177	.005586	1	.351
		4	.00171	.005016	1	1.000
OB	1	2	.03000[a]	.009375	1	.014
		3	.05800[a]	.011701	1	.000
		4	.05583[a]	.009175	1	.000
		5	.07627[a]	.012276	1	.000
	2	1	-.03000[a]	.009375	1	.014
		3	.02800[a]	.009416	1	.029
		4	.02583[a]	.006851	1	.002
		5	.04627[a]	.008306	1	.000

Pairwise Comparisons

Group	(I) Speed	(J) Speed	95% Wald Confidence Interval for Difference	
			Lower	Upper
EUT	1	2	-.00177	.05091
		3	.01170	.05516
		4	.02591	.06790
		5	.01947	.07092
	2	1	-.05091	.00177
		3	-.01643	.03414
		4	-.00116	.04582
		5	.00357	.03768
	3	1	-.05516	-.01170
		2	-.03414	.01643
		4	-.00200	.02895
		5	-.00391	.02745
	4	1	-.06790	-.02591
		2	-.04582	.00116
		3	-.02895	.00200
		5	-.01579	.01237
	5	1	-.07092	-.01947
		2	-.03768	-.00357
		3	-.02745	.00391
		4	-.01237	.01579
OB	1	2	.00368	.05632
		3	.02515	.09085
		4	.03008	.08159
		5	.04181	.11073
	2	1	-.05632	-.00368
		3	.00157	.05443
		4	.00660	.04506
		5	.02295	.06958

Pairwise Comparisons

Group	(I) Speed	(J) Speed	Mean Difference (I-J)	Std. Error	df	Bonferroni Sig.
	3	1	-.05800[a]	.011701	1	.000
		2	-.02800[a]	.009416	1	.029
		4	-.00217	.007808	1	1.000
		5	.01827	.006824	1	.074
	4	1	-.05583[a]	.009175	1	.000
		2	-.02583[a]	.006851	1	.002
		3	.00217	.007808	1	1.000
		5	.02043[a]	.007253	1	.048
	5	1	-.07627[a]	.012276	1	.000
		2	-.04627[a]	.008306	1	.000
		3	-.01827	.006824	1	.074
		4	-.02043[a]	.007253	1	.048

Pairwise Comparisons

Group	(I) Speed	(J) Speed	95% Wald Confidence Interval for Difference	
			Lower	Upper
	3	1	-.09085	-.02515
		2	-.05443	-.00157
		4	-.02408	.01975
		5	-.00089	.03742
	4	1	-.08159	-.03008
		2	-.04506	-.00660
		3	-.01975	.02408
		5	.00007	.04079
	5	1	-.11073	-.04181
		2	-.06958	-.02295
		3	-.03742	.00089
		4	-.04079	-.00007

Pairwise comparisons of estimated marginal means based on the original scale of dependent variable MPEE Released (J/kg/m)

a. The mean difference is significant at the .05 level.

Overall Test Results

Group	Wald Chi-Square	df	Sig.
EUT	40.205	4	.000
OB	50.742	4	.000

Each Wald chi-square tests the simple effects of Speed within each level combination of the other factors shown. These tests are based on the linearly independent pairwise comparisons among the estimated marginal means.

```
CORRELATIONS
  /VARIABLES=PCI Recovery MPEEUsaged
  /PRINT=TWOTAIL NOSIG
  /STATISTICS DESCRIPTIVES
  /MISSING=PAIRWISE.
```

Correlations

Notes

Output Created		12-MAY-2023 12:23:50
Comments		
Input	Data	C: \Users\andre\Documents\Andre\Pesquisa\Artigos para Publicar\Henrique Bianchi Mestrado\Statistics\Statistics Sheets\Statistics_Sheet_OB.sav
	Active Dataset	DataSet6
	Filter	<none>
	Weight	<none>
	Split File	<none>
	N of Rows in Working Data File	32
Missing Value Handling	Definition of Missing	User-defined missing values are treated as missing.
	Cases Used	Statistics for each pair of variables are based on all the cases with valid data for that pair.
Syntax		CORRELATIONS /VARIABLES=PCI Recovery MPEEUsaged /PRINT=TWOTAIL NOSIG /STATISTICS DESCRIPTIVES /MISSING=PAIRWISE.
Resources	Processor Time	00:00:00.02
	Elapsed Time	00:00:00.01

Descriptive Statistics

	Mean	Std. Deviation	N
PCI (°)	5.284	3.5040	32
Recovery (%)	54.823	14.6292	32
MPEE Usaged (J/kg/m)	.06169	.024757	32

Correlations

		PCI (°)	Recovery (%)	MPEE Usaged (J/kg/m)
PCI (°)	Pearson Correlation	1	-.627**	.485**
	Sig. (2-tailed)		.000	.005
	N	32	32	32
Recovery (%)	Pearson Correlation	-.627**	1	-.750**
	Sig. (2-tailed)	.000		.000
	N	32	32	32
MPEE Usaged (J/kg/m)	Pearson Correlation	.485**	-.750**	1
	Sig. (2-tailed)	.005	.000	
	N	32	32	32

**. Correlation is significant at the 0.01 level (2-tailed).

```
 GET
  FILE='C:\Users\andre\Documents\Andre\Pesquisa\Artigos para Publicar\Henri
que Bianchi Mestrado\Statistics\Statistics Sheets\Statistics_Sheet_EUT.sav'
.
DATASET NAME DataSet7 WINDOW=FRONT.
CORRELATIONS
  /VARIABLES=PCI Recovery MPEEUsaged
  /PRINT=TWOTAIL NOSIG
  /STATISTICS DESCRIPTIVES
  /MISSING=PAIRWISE.
```

Correlations

Notes

Output Created		12-MAY-2023 12:24:19
Comments		
Input	Data	C:\Users\andre\Documents\Andre\Pesquisa\Artigos para Publicar\Henrique Bianchi Mestrado\Statistics\Statistics Sheets\Statistics_Sheet_EUT.sav
	Active Dataset	DataSet7
	Filter	<none>
	Weight	<none>
	Split File	<none>
	N of Rows in Working Data File	36
Missing Value Handling	Definition of Missing	User-defined missing values are treated as missing.
	Cases Used	Statistics for each pair of variables are based on all the cases with valid data for that pair.
Syntax		CORRELATIONS /VARIABLES=PCI Recovery MPEEUsaged /PRINT=TWOTAIL NOSIG /STATISTICS DESCRIPTIVES /MISSING=PAIRWISE.
Resources	Processor Time	00:00:00.02
	Elapsed Time	00:00:00.01

```
[DataSet7] C:\Users\andre\Documents\Andre\Pesquisa\Artigos para Publicar\Henrique Bianchi Mestrado\Statistics\Statistics Sheets\Statistics_Sheet_EUT.sav
```

Descriptive Statistics

	Mean	Std. Deviation	N
PCI (°)	5.635	4.2537	36
Recovery (%)	61.657	12.2871	36
MPEE Usaged (J/kg/m)	.03725	.024000	36

Correlations

		PCI (°)	Recovery (%)	MPEE Usaged (J/kg/m)
PCI (°)	Pearson Correlation	1	-.604**	.342*
	Sig. (2-tailed)		.000	.041
	N	36	36	36
Recovery (%)	Pearson Correlation	-.604**	1	-.710**
	Sig. (2-tailed)	.000		.000
	N	36	36	36
MPEE Usaged (J/kg/m)	Pearson Correlation	.342*	-.710**	1
	Sig. (2-tailed)	.041	.000	
	N	36	36	36

**. Correlation is significant at the 0.01 level (2-tailed).

*. Correlation is significant at the 0.05 level (2-tailed).

```
 DATASET ACTIVATE DataSet6.
DATASET CLOSE DataSet7.
```

```
 DATASET ACTIVATE DataSet1.
* Generalized Estimating Equations.
GENLIN Recovery BY Group Speed (ORDER=ASCENDING)
  /MODEL Group Speed INTERCEPT=YES
 DISTRIBUTION=NORMAL LINK=IDENTITY
  /CRITERIA SCALE=MLE PCONVERGE=1E-006(ABSOLUTE) SINGULAR=1E-012 ANALYSISTY
PE=3(WALD) CILEVEL=95
    LIKELIHOOD=FULL
  /EMMEANS TABLES=Group SCALE=ORIGINAL COMPARE=Group CONTRAST=PAIRWISE PADJ
UST=LSD
  /EMMEANS TABLES=Speed SCALE=ORIGINAL COMPARE=Speed CONTRAST=PAIRWISE PADJ
UST=LSD
  /REPEATED SUBJECT=Subject SORT=YES CORRTYPE=INDEPENDENT ADJUSTCORR=YES CO
VB=ROBUST
  /MISSING CLASSMISSING=EXCLUDE
  /PRINT CPS DESCRIPTIVES MODELINFO FIT SUMMARY SOLUTION.
```

COVARIANCE TESTS FOR LOWER LIMB LENGTH

Generalized Linear Models

Notes

Output Created		14-AUG-2023 13:23:40
Comments		
Input	Data	C: \Users\andre\Documents\Andre\Pesquisa\Artigos para Publicar\Henrique Bianchi Mestrado\Statistics\Statistics Sheets\Statistics_Sheet_withFroude.sav
	Active Dataset	DataSet1
	Filter	<none>
	Weight	<none>
	Split File	<none>
	N of Rows in Working Data File	68
Missing Value Handling	Definition of Missing	User-defined missing values for factor, subject and within-subject variables are treated as missing.
	Cases Used	Statistics are based on cases with valid data for all variables in the model.
Weight Handling		not applicable

Notes

Syntax		GENLIN Recovery BY Group Speed (ORDER=ASCENDING)<br>/MODEL Group Speed INTERCEPT=YES<br><br>DISTRIBUTION=NORMAL LINK=IDENTITY<br>/CRITERIA SCALE=MLE PCONVERGE=1E-006 (ABSOLUTE) SINGULAR=1E-012 ANALYSISTYPE=3 (WALD) CILEVEL=95<br>LIKELIHOOD=FULL<br>/EMMEANS TABLES=Group SCALE=ORIGINAL COMPARE=Group CONTRAST=PAIRWISE PADJUST=LSD<br>/EMMEANS TABLES=Speed SCALE=ORIGINAL COMPARE=Speed CONTRAST=PAIRWISE PADJUST=LSD<br>/REPEATED SUBJECT=Subject SORT=YES CORRTYPE=INDEPENDENT ADJUSTCORR=YES COVB=ROBUST<br>/MISSING CLASSMISSING=EXCLUDE<br>/PRINT CPS DESCRIPTIVES MODELINFO FIT SUMMARY SOLUTION.
Resources	Processor Time	00:00:00.05
	Elapsed Time	00:00:00.05

Model Information

Dependent Variable		Recovery (%)
Probability Distribution		Normal
Link Function		Identity
Subject Effect	1	Subject
Working Correlation Matrix Structure		Independent

Case Processing Summary

	N	Percent
Included	68	100.0%
Excluded	0	0.0%
Total	68	100.0%

Correlated Data Summary

Number of Levels	Subject Effect	Subject	17
Number of Subjects			17
Number of Measurements per Subject	Minimum		2
	Maximum		5
Correlation Matrix Dimension			5

Categorical Variable Information

			N	Percent
Factor	Group	EUT	36	52.9%
		OB	32	47.1%
		Total	68	100.0%
	Speed	1	16	23.5%
		2	14	20.6%
		3	13	19.1%
		4	12	17.6%
		5	13	19.1%
		Total	68	100.0%

Continuous Variable Information

		N	Minimum	Maximum	Mean	Std. Deviation
Dependent Variable	Recovery (%)	68	28.7	83.1	58.441	13.7730

Goodness of Fit[a]

	Value
Quasi Likelihood under Independence Model Criterion (QIC)[b]	2890.421
Corrected Quasi Likelihood under Independence Model Criterion (QICC)[b]	2889.219

Dependent Variable: Recovery (%)
Model: (Intercept), Group, Speed

a. Information criteria are in smaller-is-better form.

b. Computed using the full log quasi-likelihood function.

Tests of Model Effects

Source	Type III		
	Wald Chi-Square	df	Sig.
(Intercept)	3368.519	1	.000
Group	5.821	1	.016
Speed	255.698	4	.000

Dependent Variable: Recovery (%)
Model: (Intercept), Group, Speed

Parameter Estimates

Parameter	B	Std. Error	95% Wald Confidence Interval		Hypothesis Test	
			Lower	Upper	Wald Chi-Square	df
(Intercept)	68.983	2.6165	63.855	74.111	695.075	1
[Group=1]	5.027	2.0833	.943	9.110	5.821	1
[Group=2]	0[a]	.	.	.	.	.
[Speed=1]	-29.178	2.9983	-35.054	-23.301	94.699	1
[Speed=2]	-21.789	2.2453	-26.190	-17.389	94.179	1
[Speed=3]	-6.363	2.6006	-11.460	-1.265	5.986	1
[Speed=4]	-3.599	2.2769	-8.061	.864	2.498	1
[Speed=5]	0[a]	.	.	.	.	.
(Scale)	46.407					

Parameter Estimates

Parameter	Hypothesis .. Sig.
(Intercept)	.000
[Group=1]	.016
[Group=2]	.
[Speed=1]	.000
[Speed=2]	.000
[Speed=3]	.014
[Speed=4]	.114
[Speed=5]	.
(Scale)	

Dependent Variable: Recovery (%)
Model: (Intercept), Group, Speed

a. Set to zero because this parameter is redundant.

Estimated Marginal Means 1: Group

Estimates

Group	Mean	Std. Error	95% Wald Confidence Interval	
			Lower	Upper
EUT	61.824	1.3447	59.188	64.459
OB	56.797	1.5654	53.729	59.865

Pairwise Comparisons

(I) Group	(J) Group	Mean Difference (I-J)	Std. Error	df	Sig.	95% Wald Confidence ... Lower
EUT	OB	5.027[a]	2.0833	1	.016	.943
OB	EUT	-5.027[a]	2.0833	1	.016	-9.110

Pairwise Comparisons

(I) Group	(J) Group	95% Wald Confidence ... Upper
EUT	OB	9.110
OB	EUT	-.943

Pairwise comparisons of estimated marginal means based on the original scale of dependent variable Recovery (%)

a. The mean difference is significant at the .05 level.

Overall Test Results

Wald Chi-Square	df	Sig.
5.821	1	.016

The Wald chi-square tests the effect of Group. This test is based on the linearly independent pairwise comparisons among the estimated marginal means.

Estimated Marginal Means 2: Speed

Estimates

Speed	Mean	Std. Error	95% Wald Confidence Interval	
			Lower	Upper
1	42.319	2.0041	38.391	46.247
2	49.707	1.4251	46.914	52.500
3	65.134	1.2753	62.634	67.633
4	67.898	1.6742	64.616	71.179
5	71.496	2.1628	67.257	75.735

Pairwise Comparisons

(I) Speed	(J) Speed	Mean Difference (I-J)	Std. Error	df	Sig.	95% Wald Confidence ...
						Lower
1	2	-7.388[a]	1.9348	1	.000	-11.180
	3	-22.815[a]	1.8905	1	.000	-26.520
	4	-25.579[a]	2.5306	1	.000	-30.539
	5	-29.178[a]	2.9983	1	.000	-35.054
2	1	7.388[a]	1.9348	1	.000	3.596
	3	-15.427[a]	1.5029	1	.000	-18.373
	4	-18.191[a]	2.1149	1	.000	-22.336
	5	-21.789[a]	2.2453	1	.000	-26.190
3	1	22.815[a]	1.8905	1	.000	19.110
	2	15.427[a]	1.5029	1	.000	12.481
	4	-2.764	1.7980	1	.124	-6.288
	5	-6.363[a]	2.6006	1	.014	-11.460
4	1	25.579[a]	2.5306	1	.000	20.619
	2	18.191[a]	2.1149	1	.000	14.046

Pairwise Comparisons

(I) Speed	(J) Speed	95% Wald Confidence ... Upper
1	2	-3.596
	3	-19.110
	4	-20.619
	5	-23.301
2	1	11.180
	3	-12.481
	4	-14.046
	5	-17.389
3	1	26.520
	2	18.373
	4	.760
	5	-1.265
4	1	30.539
	2	22.336

Pairwise Comparisons

(I) Speed	(J) Speed	Mean Difference (I-J)	Std. Error	df	Sig.	95% Wald Confidence ... Lower
	3	2.764	1.7980	1	.124	-.760
	5	-3.599	2.2769	1	.114	-8.061
5	1	29.178[a]	2.9983	1	.000	23.301
	2	21.789[a]	2.2453	1	.000	17.389
	3	6.363[a]	2.6006	1	.014	1.265
	4	3.599	2.2769	1	.114	-.864

Pairwise Comparisons

(I) Speed	(J) Speed	95% Wald Confidence ... Upper
	3	6.288
	5	.864
5	1	35.054
	2	26.190
	3	11.460
	4	8.061

Pairwise comparisons of estimated marginal means based on the original scale of dependent variable Recovery (%)

a. The mean difference is significant at the .05 level.

Overall Test Results

Wald Chi-Square	df	Sig.
255.698	4	.000

The Wald chi-square tests the effect of Speed. This test is based on the linearly independent pairwise comparisons among the estimated marginal means.

```
 * Generalized Estimating Equations.
GENLIN Recovery BY Group Speed (ORDER=ASCENDING) WITH CMI
  /MODEL Group Speed CMI INTERCEPT=YES
 DISTRIBUTION=NORMAL LINK=IDENTITY
  /CRITERIA SCALE=MLE PCONVERGE=1E-006(ABSOLUTE) SINGULAR=1E-012 ANALYSISTY
PE=3(WALD) CILEVEL=95
    LIKELIHOOD=FULL
  /EMMEANS TABLES=Group SCALE=ORIGINAL COMPARE=Group CONTRAST=PAIRWISE PADJ
UST=LSD
  /EMMEANS TABLES=Speed SCALE=ORIGINAL COMPARE=Speed CONTRAST=PAIRWISE PADJ
UST=LSD
  /REPEATED SUBJECT=Subject SORT=YES CORRTYPE=INDEPENDENT ADJUSTCORR=YES CO
VB=ROBUST
  /MISSING CLASSMISSING=EXCLUDE
  /PRINT CPS DESCRIPTIVES MODELINFO FIT SUMMARY SOLUTION.
```

Generalized Linear Models

Notes

Output Created		14-AUG-2023 13:23:40
Comments		
Input	Data	C: \Users\andre\Documents\Andre\Pesquisa\Artigos para Publicar\Henrique Bianchi Mestrado\Statistics\Statistics Sheets\Statistics_Sheet_withFroude.sav
	Active Dataset	DataSet1
	Filter	<none>
	Weight	<none>
	Split File	<none>
	N of Rows in Working Data File	68
Missing Value Handling	Definition of Missing	User-defined missing values for factor, subject and within-subject variables are treated as missing.
	Cases Used	Statistics are based on cases with valid data for all variables in the model.
Weight Handling		not applicable

Notes

Syntax		GENLIN Recovery BY Group Speed (ORDER=ASCENDING) WITH CMI<br>/MODEL Group Speed CMI INTERCEPT=YES<br><br>DISTRIBUTION=NORMAL LINK=IDENTITY<br>/CRITERIA SCALE=MLE PCONVERGE=1E-006 (ABSOLUTE) SINGULAR=1E-012 ANALYSISTYPE=3 (WALD) CILEVEL=95<br>LIKELIHOOD=FULL<br>/EMMEANS TABLES=Group SCALE=ORIGINAL COMPARE=Group CONTRAST=PAIRWISE PADJUST=LSD<br>/EMMEANS TABLES=Speed SCALE=ORIGINAL COMPARE=Speed CONTRAST=PAIRWISE PADJUST=LSD<br>/REPEATED SUBJECT=Subject SORT=YES CORRTYPE=INDEPENDENT ADJUSTCORR=YES COVB=ROBUST<br>/MISSING CLASSMISSING=EXCLUDE<br>/PRINT CPS DESCRIPTIVES MODELINFO FIT SUMMARY SOLUTION.
Resources	Processor Time	00:00:00.05
	Elapsed Time	00:00:00.04

Model Information

Dependent Variable		Recovery (%)
Probability Distribution		Normal
Link Function		Identity
Subject Effect	1	Subject
Working Correlation Matrix Structure		Independent

Case Processing Summary

	N	Percent
Included	68	100.0%
Excluded	0	0.0%
Total	68	100.0%

Correlated Data Summary

Number of Levels	Subject Effect	Subject	17
Number of Subjects			17
Number of Measurements per Subject	Minimum		2
	Maximum		5
Correlation Matrix Dimension			5

Categorical Variable Information

			N	Percent
Factor	Group	EUT	36	52.9%
		OB	32	47.1%
		Total	68	100.0%
	Speed	1	16	23.5%
		2	14	20.6%
		3	13	19.1%
		4	12	17.6%
		5	13	19.1%
		Total	68	100.0%

Continuous Variable Information

		N	Minimum	Maximum	Mean	Std. Deviation
Dependent Variable	Recovery (%)	68	28.7	83.1	58.441	13.7730
Covariate	CMI	68	.620	.835	.69147	.053577

Goodness of Fit[a]

	Value
Quasi Likelihood under Independence Model Criterion (QIC)[b]	2676.329
Corrected Quasi Likelihood under Independence Model Criterion (QICC)[b]	2674.337

Dependent Variable: Recovery (%)
Model: (Intercept), Group, Speed, CMI

a. Information criteria are in smaller-is-better form.

b. Computed using the full log quasi-likelihood function.

Tests of Model Effects

Source	Type III Wald Chi-Square	df	Sig.
(Intercept)	2.395	1	.122
Group	8.881	1	.003
Speed	221.992	4	.000
CMI	3.203	1	.074

Dependent Variable: Recovery (%)
Model: (Intercept), Group, Speed, CMI

Parameter Estimates

Parameter	B	Std. Error	95% Wald Confidence Interval Lower	Upper	Hypothesis Test Wald Chi-Square	df
(Intercept)	35.282	18.7395	-1.447	72.011	3.545	1
[Group=1]	8.360	2.8053	2.862	13.858	8.881	1
[Group=2]	0[a]	.	.	.	.	.
[Speed=1]	-29.035	2.9566	-34.830	-23.240	96.439	1
[Speed=2]	-21.465	2.1037	-25.588	-17.342	104.109	1
[Speed=3]	-6.159	2.5718	-11.200	-1.119	5.736	1
[Speed=4]	-3.307	2.3344	-7.883	1.268	2.007	1
[Speed=5]	0[a]	.	.	.	.	.
CMI	45.910	25.6527	-4.369	96.188	3.203	1
(Scale)	43.612					

Parameter Estimates

Parameter	Hypothesis .. Sig.
(Intercept)	.060
[Group=1]	.003
[Group=2]	.
[Speed=1]	.000
[Speed=2]	.000
[Speed=3]	.017
[Speed=4]	.157
[Speed=5]	.
CMI	.074
(Scale)	

Dependent Variable: Recovery (%)
Model: (Intercept), Group, Speed, CMI

a. Set to zero because this parameter is redundant.

Estimated Marginal Means 1: Group

Estimates

Group	Mean	Std. Error	95% Wald Confidence Interval	
			Lower	Upper
EUT	63.394	1.7045	60.053	66.735
OB	55.034	1.6043	51.890	58.179

Covariates appearing in the model are fixed at the following values: CMI=.69147

Pairwise Comparisons

(I) Group	(J) Group	Mean Difference (I-J)	Std. Error	df	Sig.	95% Wald Confidence ...
						Lower
EUT	OB	8.360[a]	2.8053	1	.003	2.862
OB	EUT	-8.360[a]	2.8053	1	.003	-13.858

Pairwise Comparisons

(I) Group	(J) Group	95% Wald Confidence ...
		Upper
EUT	OB	13.858
OB	EUT	-2.862

Pairwise comparisons of estimated marginal means based on the original scale of dependent variable Recovery (%)

a. The mean difference is significant at the .05 level.

Overall Test Results

Wald Chi-Square	df	Sig.
8.881	1	.003

The Wald chi-square tests the effect of Group. This test is based on the linearly independent pairwise comparisons among the estimated marginal means.

Estimated Marginal Means 2: Speed

Estimates

Speed	Mean	Std. Error	95% Wald Confidence Interval	
			Lower	Upper
1	42.173	1.9014	38.446	45.899
2	49.743	1.4144	46.970	52.515
3	65.048	1.3113	62.478	67.618
4	67.900	1.6823	64.603	71.197
5	71.207	1.8937	67.496	74.919

Covariates appearing in the model are fixed at the following values: CMI=.69147

Pairwise Comparisons

(I) Speed	(J) Speed	Mean Difference (I-J)	Std. Error	df	Sig.	95% Wald Confidence ...
						Lower
1	2	-7.570[a]	1.8455	1	.000	-11.187
	3	-22.876[a]	1.9352	1	.000	-26.669
	4	-25.727[a]	2.5507	1	.000	-30.727
	5	-29.035[a]	2.9566	1	.000	-34.830
2	1	7.570[a]	1.8455	1	.000	3.953
	3	-15.305[a]	1.6704	1	.000	-18.579
	4	-18.157[a]	2.1224	1	.000	-22.317
	5	-21.465[a]	2.1037	1	.000	-25.588
3	1	22.876[a]	1.9352	1	.000	19.083
	2	15.305[a]	1.6704	1	.000	12.032
	4	-2.852	1.8088	1	.115	-6.397
	5	-6.159[a]	2.5718	1	.017	-11.200
4	1	25.727[a]	2.5507	1	.000	20.728
	2	18.157[a]	2.1224	1	.000	13.997
	3	2.852	1.8088	1	.115	-.693
	5	-3.307	2.3344	1	.157	-7.883
5	1	29.035[a]	2.9566	1	.000	23.240
	2	21.465[a]	2.1037	1	.000	17.342
	3	6.159[a]	2.5718	1	.017	1.119
	4	3.307	2.3344	1	.157	-1.268

Pairwise Comparisons

(I) Speed	(J) Speed	95% Wald Confidence ... Upper
1	2	-3.953
	3	-19.083
	4	-20.728
	5	-23.240
2	1	11.187
	3	-12.032
	4	-13.997
	5	-17.342
3	1	26.669
	2	18.579
	4	.693
	5	-1.119
4	1	30.727
	2	22.317
	3	6.397
	5	1.268
5	1	34.830
	2	25.588
	3	11.200
	4	7.883

Pairwise comparisons of estimated marginal means based on the original scale of dependent variable Recovery (%)

a. The mean difference is significant at the .05 level.

Overall Test Results

Wald Chi-Square	df	Sig.
221.992	4	.000

The Wald chi-square tests the effect of Speed. This test is based on the linearly independent pairwise comparisons among the estimated marginal means.

```
* Generalized Estimating Equations.
```

```
GENLIN MPEEUsaged BY Group Speed (ORDER=ASCENDING)
  /MODEL Group Speed INTERCEPT=YES
 DISTRIBUTION=NORMAL LINK=IDENTITY
  /CRITERIA SCALE=MLE PCONVERGE=1E-006(ABSOLUTE) SINGULAR=1E-012 ANALYSISTY
PE=3(WALD) CILEVEL=95
    LIKELIHOOD=FULL
  /EMMEANS TABLES=Group SCALE=ORIGINAL COMPARE=Group CONTRAST=PAIRWISE PADJ
UST=LSD
  /EMMEANS TABLES=Speed SCALE=ORIGINAL COMPARE=Speed CONTRAST=PAIRWISE PADJ
UST=LSD
  /REPEATED SUBJECT=Subject SORT=YES CORRTYPE=INDEPENDENT ADJUSTCORR=YES CO
VB=ROBUST
  /MISSING CLASSMISSING=EXCLUDE
  /PRINT CPS DESCRIPTIVES MODELINFO FIT SUMMARY SOLUTION.
```

Generalized Linear Models

Notes

Output Created		14-AUG-2023 13:24:41
Comments		
Input	Data	C:\Users\andre\Documents\Andre\Pesquisa\Artigos para Publicar\Henrique Bianchi Mestrado\Statistics\Statistics Sheets\Statistics_Sheet_withFroude.sav
	Active Dataset	DataSet1
	Filter	<none>
	Weight	<none>
	Split File	<none>
	N of Rows in Working Data File	68
Missing Value Handling	Definition of Missing	User-defined missing values for factor, subject and within-subject variables are treated as missing.
	Cases Used	Statistics are based on cases with valid data for all variables in the model.
Weight Handling		not applicable

Notes

Syntax		GENLIN MPEEUsaged BY Group Speed (ORDER=ASCENDING)<br>/MODEL Group Speed INTERCEPT=YES<br><br>DISTRIBUTION=NORMAL LINK=IDENTITY<br>/CRITERIA SCALE=MLE PCONVERGE=1E-006 (ABSOLUTE) SINGULAR=1E-012 ANALYSISTYPE=3 (WALD) CILEVEL=95<br>LIKELIHOOD=FULL<br>/EMMEANS TABLES=Group SCALE=ORIGINAL COMPARE=Group CONTRAST=PAIRWISE PADJUST=LSD<br>/EMMEANS TABLES=Speed SCALE=ORIGINAL COMPARE=Speed CONTRAST=PAIRWISE PADJUST=LSD<br>/REPEATED SUBJECT=Subject SORT=YES CORRTYPE=INDEPENDENT ADJUSTCORR=YES COVB=ROBUST<br>/MISSING CLASSMISSING=EXCLUDE<br>/PRINT CPS DESCRIPTIVES MODELINFO FIT SUMMARY SOLUTION.
Resources	Processor Time	00:00:00.06
	Elapsed Time	00:00:00.07

Model Information

Dependent Variable		MPEE Usaged (J/kg/m)
Probability Distribution		Normal
Link Function		Identity
Subject Effect	1	Subject
Working Correlation Matrix Structure		Independent

Case Processing Summary

	N	Percent
Included	68	100.0%
Excluded	0	0.0%
Total	68	100.0%

Correlated Data Summary

Number of Levels	Subject Effect	Subject	17
Number of Subjects			17
Number of Measurements per Subject	Minimum		2
	Maximum		5
Correlation Matrix Dimension			5

Categorical Variable Information

			N	Percent
Factor	Group	EUT	36	52.9%
		OB	32	47.1%
		Total	68	100.0%
	Speed	1	16	23.5%
		2	14	20.6%
		3	13	19.1%
		4	12	17.6%
		5	13	19.1%
		Total	68	100.0%

Continuous Variable Information

		N	Minimum	Maximum	Mean
Dependent Variable	MPEE Usaged (J/kg/m)	68	.012	.114	.04875

Continuous Variable Information

		Std. Deviation
Dependent Variable	MPEE Usaged (J/kg/m)	.027120

Goodness of Fit[a]

	Value
Quasi Likelihood under Independence Model Criterion (QIC)[b]	12.044
Corrected Quasi Likelihood under Independence Model Criterion (QICC)[b]	12.021

Dependent Variable: MPEE Usaged (J/kg/m)
Model: (Intercept), Group, Speed

a. Information criteria are in smaller-is-better form.

b. Computed using the full log quasi-likelihood function.

Tests of Model Effects

Source	Type III Wald Chi-Square	df	Sig.
(Intercept)	441.319	1	.000
Group	20.899	1	.000
Speed	47.455	4	.000

Dependent Variable: MPEE Usaged (J/kg/m)
Model: (Intercept), Group, Speed

Parameter Estimates

Parameter	B	Std. Error	95% Wald Confidence Interval		Hypothesis Test	
			Lower	Upper	Wald Chi-Square	df
(Intercept)	.039	.0041	.032	.047	94.225	1
[Group=1]	-.022	.0047	-.031	-.012	20.899	1
[Group=2]	0[a]	.	.	.	.	.
[Speed=1]	.045	.0078	.029	.060	32.662	1
[Speed=2]	.030	.0056	.019	.041	28.508	1
[Speed=3]	.014	.0047	.005	.023	8.596	1
[Speed=4]	.008	.0051	-.002	.018	2.504	1
[Speed=5]	0[a]	.	.	.	.	.
(Scale)	.000					

Parameter Estimates

Parameter	Hypothesis .. Sig.
(Intercept)	.000
[Group=1]	.000
[Group=2]	.
[Speed=1]	.000
[Speed=2]	.000
[Speed=3]	.003
[Speed=4]	.114
[Speed=5]	.
(Scale)	

Dependent Variable: MPEE Usaged (J/kg/m)
Model: (Intercept), Group, Speed

a. Set to zero because this parameter is redundant.

Estimated Marginal Means 1: Group

Estimates

Group	Mean	Std. Error	95% Wald Confidence Interval	
			Lower	Upper
EUT	.03718	.003792	.02975	.04461
OB	.05871	.002670	.05348	.06395

Pairwise Comparisons

(I) Group	(J) Group	Mean Difference (I-J)	Std. Error	df	Sig.	95% Wald Confidence ... Lower
EUT	OB	-.02153[a]	.004709	1	.000	-.03076
OB	EUT	.02153[a]	.004709	1	.000	.01230

Pairwise Comparisons

(I) Group	(J) Group	95% Wald Confidence ... Upper
EUT	OB	-.01230
OB	EUT	.03076

Pairwise comparisons of estimated marginal means based on the original scale of dependent variable MPEE Usaged (J/kg/m)

a. The mean difference is significant at the .05 level.

Overall Test Results

Wald Chi-Square	df	Sig.
20.899	1	.000

The Wald chi-square tests the effect of Group. This test is based on the linearly independent pairwise comparisons among the estimated marginal means.

Estimated Marginal Means 2: Speed

Estimates

Speed	Mean	Std. Error	95% Wald Confidence Interval	
			Lower	Upper
1	.07328	.005576	.06235	.08421
2	.05839	.005194	.04822	.06857
3	.04260	.004716	.03335	.05184
4	.03675	.003594	.02971	.04379
5	.02871	.003469	.02192	.03551

Pairwise Comparisons

(I) Speed	(J) Speed	Mean Difference (I-J)	Std. Error	df	Sig.	95% Wald Confidence ...
						Lower
1	2	.01488[a]	.006575	1	.024	.00200
	3	.03068[a]	.007640	1	.000	.01571
	4	.03653[a]	.006965	1	.000	.02288
	5	.04456[a]	.007798	1	.000	.02928
2	1	-.01488[a]	.006575	1	.024	-.02777
	3	.01580[a]	.006814	1	.020	.00244
	4	.02164[a]	.005783	1	.000	.01031
	5	.02968[a]	.005559	1	.000	.01878
3	1	-.03068[a]	.007640	1	.000	-.04566
	2	-.01580[a]	.006814	1	.020	-.02915
	4	.00585	.005113	1	.253	-.00417
	5	.01388[a]	.004735	1	.003	.00460
4	1	-.03653[a]	.006965	1	.000	-.05018
	2	-.02164[a]	.005783	1	.000	-.03298

Pairwise Comparisons

(I) Speed	(J) Speed	95% Wald Confidence ... Upper
1	2	.02777
	3	.04566
	4	.05018
	5	.05985
2	1	-.00200
	3	.02915
	4	.03298
	5	.04058
3	1	-.01571
	2	-.00244
	4	.01587
	5	.02316
4	1	-.02288
	2	-.01031

Pairwise Comparisons

(I) Speed	(J) Speed	Mean Difference (I-J)	Std. Error	df	Sig.	95% Wald Confidence ... Lower
	3	-.00585	.005113	1	.253	-.01587
	5	.00804	.005078	1	.114	-.00192
5	1	-.04456[a]	.007798	1	.000	-.05985
	2	-.02968[a]	.005559	1	.000	-.04058
	3	-.01388[a]	.004735	1	.003	-.02316
	4	-.00804	.005078	1	.114	-.01799

Pairwise Comparisons

(I) Speed	(J) Speed	95% Wald Confidence ... Upper
	3	.00417
	5	.01799
5	1	-.02928
	2	-.01878
	3	-.00460
	4	.00192

Pairwise comparisons of estimated marginal means based on the original scale of dependent variable MPEE Usaged (J/kg/m)

a. The mean difference is significant at the .05 level.

Overall Test Results

Wald Chi-Square	df	Sig.
47.455	4	.000

The Wald chi-square tests the effect of Speed. This test is based on the linearly independent pairwise comparisons among the estimated marginal means.

```
 * Generalized Estimating Equations.
GENLIN MPEEUsaged BY Group Speed (ORDER=ASCENDING) WITH CMI
  /MODEL Group Speed CMI INTERCEPT=YES
 DISTRIBUTION=NORMAL LINK=IDENTITY
  /CRITERIA SCALE=MLE PCONVERGE=1E-006(ABSOLUTE) SINGULAR=1E-012 ANALYSISTY
PE=3(WALD) CILEVEL=95
    LIKELIHOOD=FULL
  /EMMEANS TABLES=Group SCALE=ORIGINAL COMPARE=Group CONTRAST=PAIRWISE PADJ
UST=LSD
  /EMMEANS TABLES=Speed SCALE=ORIGINAL COMPARE=Speed CONTRAST=PAIRWISE PADJ
UST=LSD
  /REPEATED SUBJECT=Subject SORT=YES CORRTYPE=INDEPENDENT ADJUSTCORR=YES CO
VB=ROBUST
  /MISSING CLASSMISSING=EXCLUDE
  /PRINT CPS DESCRIPTIVES MODELINFO FIT SUMMARY SOLUTION.
```

Generalized Linear Models

Notes

Output Created		14-AUG-2023 13:24:41
Comments		
Input	Data	C: \Users\andre\Documents\Andre\Pesquisa\Artigos para Publicar\Henrique Bianchi Mestrado\Statistics\Statistics Sheets\Statistics_Sheet_withFroude.sav
	Active Dataset	DataSet1
	Filter	<none>
	Weight	<none>
	Split File	<none>
	N of Rows in Working Data File	68
Missing Value Handling	Definition of Missing	User-defined missing values for factor, subject and within-subject variables are treated as missing.
	Cases Used	Statistics are based on cases with valid data for all variables in the model.
Weight Handling		not applicable

Notes

Syntax		GENLIN MPEEUsaged BY Group Speed (ORDER=ASCENDING) WITH CMI<br>/MODEL Group Speed CMI INTERCEPT=YES<br><br>DISTRIBUTION=NORMAL LINK=IDENTITY<br>/CRITERIA SCALE=MLE PCONVERGE=1E-006 (ABSOLUTE) SINGULAR=1E-012 ANALYSISTYPE=3 (WALD) CILEVEL=95<br>LIKELIHOOD=FULL<br>/EMMEANS TABLES=Group SCALE=ORIGINAL COMPARE=Group CONTRAST=PAIRWISE PADJUST=LSD<br>/EMMEANS TABLES=Speed SCALE=ORIGINAL COMPARE=Speed CONTRAST=PAIRWISE PADJUST=LSD<br>/REPEATED SUBJECT=Subject SORT=YES CORRTYPE=INDEPENDENT ADJUSTCORR=YES COVB=ROBUST<br>/MISSING CLASSMISSING=EXCLUDE<br>/PRINT CPS DESCRIPTIVES MODELINFO FIT SUMMARY SOLUTION.
Resources	Processor Time	00:00:00.05
	Elapsed Time	00:00:00.04

Model Information

Dependent Variable		MPEE Usaged (J/kg/m)
Probability Distribution		Normal
Link Function		Identity
Subject Effect	1	Subject
Working Correlation Matrix Structure		Independent

Case Processing Summary

	N	Percent
Included	68	100.0%
Excluded	0	0.0%
Total	68	100.0%

Correlated Data Summary

Number of Levels	Subject Effect	Subject	17
Number of Subjects			17
Number of Measurements per Subject	Minimum		2
	Maximum		5
Correlation Matrix Dimension			5

Categorical Variable Information

			N	Percent
Factor	Group	EUT	36	52.9%
		OB	32	47.1%
		Total	68	100.0%
	Speed	1	16	23.5%
		2	14	20.6%
		3	13	19.1%
		4	12	17.6%
		5	13	19.1%
		Total	68	100.0%

Continuous Variable Information

		N	Minimum	Maximum	Mean
Dependent Variable	MPEE Usaged (J/kg/m)	68	.012	.114	.04875
Covariate	CMI	68	.620	.835	.69147

Continuous Variable Information

		Std. Deviation
Dependent Variable	MPEE Usaged (J/kg/m)	.027120
Covariate	CMI	.053577

Goodness of Fit[a]

	Value
Quasi Likelihood under Independence Model Criterion (QIC)[b]	13.159
Corrected Quasi Likelihood under Independence Model Criterion (QICC)[b]	14.021

Dependent Variable: MPEE Usaged (J/kg/m)
Model: (Intercept), Group, Speed, CMI

a. Information criteria are in smaller-is-better form.

b. Computed using the full log quasi-likelihood function.

Tests of Model Effects

Source	Type III		
	Wald Chi-Square	df	Sig.
(Intercept)	.161	1	.688
Group	7.463	1	.006
Speed	47.147	4	.000
CMI	1.507	1	.220

Dependent Variable: MPEE Usaged (J/kg/m)
Model: (Intercept), Group, Speed, CMI

Parameter Estimates

Parameter	B	Std. Error	95% Wald Confidence Interval Lower	95% Wald Confidence Interval Upper	Hypothesis Test Wald Chi-Square	Hypothesis Test df	Hypothesis Test Sig.
(Intercept)	.001	.0326	-.063	.065	.002	1	.965
[Group=1]	-.018	.0065	-.031	-.005	7.463	1	.006
[Group=2]	0[a]	.	.	.	.	.	.
[Speed=1]	.045	.0078	.030	.060	33.177	1	.000
[Speed=2]	.030	.0056	.019	.041	28.673	1	.000
[Speed=3]	.014	.0046	.005	.023	9.332	1	.002
[Speed=4]	.008	.0050	-.001	.018	2.805	1	.094
[Speed=5]	0[a]	.	.	.	.	.	.
CMI	.052	.0422	-.031	.135	1.507	1	.220
(Scale)	.000						

Parameter Estimates

Dependent Variable: MPEE Usaged (J/kg/m)
Model: (Intercept), Group, Speed, CMI

a. Set to zero because this parameter is redundant.

Estimated Marginal Means 1: Group

Estimates

Group	Mean	Std. Error	95% Wald Confidence Interval	
			Lower	Upper
EUT	.03896	.004278	.03057	.04734
OB	.05672	.003599	.04967	.06377

Covariates appearing in the model are fixed at the following values: CMI=.69147

Pairwise Comparisons

(I) Group	(J) Group	Mean Difference (I-J)	Std. Error	df	Sig.	95% Wald Confidence ...
						Lower
EUT	OB	-.01776[a]	.006503	1	.006	-.03051
OB	EUT	.01776[a]	.006503	1	.006	.00502

Pairwise Comparisons

(I) Group	(J) Group	95% Wald Confidence ...
		Upper
EUT	OB	-.00502
OB	EUT	.03051

Pairwise comparisons of estimated marginal means based on the original scale of dependent variable MPEE Usaged (J/kg/m)

a. The mean difference is significant at the .05 level.

Overall Test Results

Wald Chi-Square	df	Sig.
7.463	1	.006

The Wald chi-square tests the effect of Group. This test is based on the linearly independent pairwise comparisons among the estimated marginal means.

Estimated Marginal Means 2: Speed

Estimates

Speed	Mean	Std. Error	95% Wald Confidence Interval	
			Lower	Upper
1	.07311	.005691	.06196	.08427
2	.05844	.005177	.04829	.06858
3	.04250	.004406	.03386	.05114
4	.03675	.003453	.02999	.04352
5	.02839	.003559	.02141	.03536

Covariates appearing in the model are fixed at the following values: CMI=.69147

Pairwise Comparisons

(I) Speed	(J) Speed	Mean Difference (I-J)	Std. Error	df	Sig.	95% Wald Confidence ...
						Lower
1	2	.01468[a]	.006653	1	.027	.00164
	3	.03061[a]	.007499	1	.000	.01592
	4	.03636[a]	.006906	1	.000	.02283
	5	.04473[a]	.007765	1	.000	.02951
2	1	-.01468[a]	.006653	1	.027	-.02772
	3	.01593[a]	.006628	1	.016	.00294
	4	.02168[a]	.005766	1	.000	.01038
	5	.03005[a]	.005611	1	.000	.01905
3	1	-.03061[a]	.007499	1	.000	-.04531
	2	-.01593[a]	.006628	1	.016	-.02892
	4	.00575	.005115	1	.261	-.00428
	5	.01411[a]	.004620	1	.002	.00506
4	1	-.03636[a]	.006906	1	.000	-.04990
	2	-.02168[a]	.005766	1	.000	-.03298
	3	-.00575	.005115	1	.261	-.01577
	5	.00836	.004994	1	.094	-.00142
5	1	-.04473[a]	.007765	1	.000	-.05995
	2	-.03005[a]	.005611	1	.000	-.04104
	3	-.01411[a]	.004620	1	.002	-.02317
	4	-.00836	.004994	1	.094	-.01815

Pairwise Comparisons

(I) Speed	(J) Speed	95% Wald Confidence ... Upper
1	2	.02772
	3	.04531
	4	.04990
	5	.05995
2	1	-.00164
	3	.02892
	4	.03298
	5	.04104
3	1	-.01592
	2	-.00294
	4	.01577
	5	.02317
4	1	-.02283
	2	-.01038
	3	.00428
	5	.01815
5	1	-.02951
	2	-.01905
	3	-.00506
	4	.00142

Pairwise comparisons of estimated marginal means based on the original scale of dependent variable MPEE Usaged (J/kg/m)

a. The mean difference is significant at the .05 level.

Overall Test Results

Wald Chi-Square	df	Sig.
47.147	4	.000

The Wald chi-square tests the effect of Speed. This test is based on the linearly independent pairwise comparisons among the estimated marginal means.

```
* Generalized Estimating Equations.
```

```
GENLIN PCI BY Group Speed (ORDER=ASCENDING)
  /MODEL Group Speed INTERCEPT=YES
 DISTRIBUTION=NORMAL LINK=IDENTITY
  /CRITERIA SCALE=MLE PCONVERGE=1E-006(ABSOLUTE) SINGULAR=1E-012 ANALYSISTY
PE=3(WALD) CILEVEL=95
    LIKELIHOOD=FULL
  /EMMEANS TABLES=Group SCALE=ORIGINAL COMPARE=Group CONTRAST=PAIRWISE PADJ
UST=LSD
  /EMMEANS TABLES=Speed SCALE=ORIGINAL COMPARE=Speed CONTRAST=PAIRWISE PADJ
UST=LSD
  /REPEATED SUBJECT=Subject SORT=YES CORRTYPE=INDEPENDENT ADJUSTCORR=YES CO
VB=ROBUST
  /MISSING CLASSMISSING=EXCLUDE
  /PRINT CPS DESCRIPTIVES MODELINFO FIT SUMMARY SOLUTION.
```

Generalized Linear Models

Notes

Output Created		14-AUG-2023 13:25:36
Comments		
Input	Data	C:\Users\andre\Documents\Andre\Pesquisa\Artigos para Publicar\Henrique Bianchi Mestrado\Statistics\Statistics Sheets\Statistics_Sheet_withFroude.sav
	Active Dataset	DataSet1
	Filter	<none>
	Weight	<none>
	Split File	<none>
	N of Rows in Working Data File	68
Missing Value Handling	Definition of Missing	User-defined missing values for factor, subject and within-subject variables are treated as missing.
	Cases Used	Statistics are based on cases with valid data for all variables in the model.
Weight Handling		not applicable

Notes

Syntax		GENLIN PCI BY Group Speed (ORDER=ASCENDING)<br>/MODEL Group Speed INTERCEPT=YES<br><br>DISTRIBUTION=NORMAL LINK=IDENTITY<br>/CRITERIA SCALE=MLE PCONVERGE=1E-006 (ABSOLUTE) SINGULAR=1E-012 ANALYSISTYPE=3 (WALD) CILEVEL=95<br>LIKELIHOOD=FULL<br>/EMMEANS TABLES=Group SCALE=ORIGINAL COMPARE=Group CONTRAST=PAIRWISE PADJUST=LSD<br>/EMMEANS TABLES=Speed SCALE=ORIGINAL COMPARE=Speed CONTRAST=PAIRWISE PADJUST=LSD<br>/REPEATED SUBJECT=Subject SORT=YES CORRTYPE=INDEPENDENT ADJUSTCORR=YES COVB=ROBUST<br>/MISSING CLASSMISSING=EXCLUDE<br>/PRINT CPS DESCRIPTIVES MODELINFO FIT SUMMARY SOLUTION.
Resources	Processor Time	00:00:00.09
	Elapsed Time	00:00:00.06

Model Information

Dependent Variable		PCI (°)
Probability Distribution		Normal
Link Function		Identity
Subject Effect	1	Subject
Working Correlation Matrix Structure		Independent

Case Processing Summary

	N	Percent
Included	68	100.0%
Excluded	0	0.0%
Total	68	100.0%

Correlated Data Summary

Number of Levels	Subject Effect	Subject	17
Number of Subjects			17
Number of Measurements per Subject	Minimum		2
	Maximum		5
Correlation Matrix Dimension			5

Categorical Variable Information

			N	Percent
Factor	Group	EUT	36	52.9%
		OB	32	47.1%
		Total	68	100.0%
	Speed	1	16	23.5%
		2	14	20.6%
		3	13	19.1%
		4	12	17.6%
		5	13	19.1%
		Total	68	100.0%

Continuous Variable Information

		N	Minimum	Maximum	Mean	Std. Deviation
Dependent Variable	PCI (°)	68	1.2	18.8	5.470	3.8941

Goodness of Fit[a]

	Value
Quasi Likelihood under Independence Model Criterion (QIC)[b]	489.846
Corrected Quasi Likelihood under Independence Model Criterion (QICC)[b]	489.612

Dependent Variable: PCI (°)
Model: (Intercept), Group, Speed

a. Information criteria are in smaller-is-better form.

b. Computed using the full log quasi-likelihood function.

Tests of Model Effects

Source	Type III		
	Wald Chi-Square	df	Sig.
(Intercept)	212.755	1	.000
Group	1.028	1	.311
Speed	72.053	4	.000

Dependent Variable: PCI (°)
Model: (Intercept), Group, Speed

Parameter Estimates

Parameter	B	Std. Error	95% Wald Confidence Interval		Hypothesis Test	
			Lower	Upper	Wald Chi-Square	df
(Intercept)	1.908	.4034	1.117	2.698	22.367	1
[Group=1]	.820	.8084	-.765	2.404	1.028	1
[Group=2]	0[a]	.	.	.	.	.
[Speed=1]	7.220	1.0419	5.178	9.262	48.017	1
[Speed=2]	4.919	.8040	3.344	6.495	37.440	1
[Speed=3]	1.254	.4586	.355	2.153	7.475	1
[Speed=4]	1.001	.4182	.181	1.820	5.724	1
[Speed=5]	0[a]	.	.	.	.	.
(Scale)	7.703					

Parameter Estimates

Parameter	Hypothesis .. Sig.
(Intercept)	.000
[Group=1]	.311
[Group=2]	.
[Speed=1]	.000
[Speed=2]	.000
[Speed=3]	.006
[Speed=4]	.017
[Speed=5]	.
(Scale)	

Dependent Variable: PCI (°)
Model: (Intercept), Group, Speed

a. Set to zero because this parameter is redundant.

Estimated Marginal Means 1: Group

Estimates

Group	Mean	Std. Error	95% Wald Confidence Interval Lower	95% Wald Confidence Interval Upper
EUT	5.606	.6761	4.281	6.932
OB	4.787	.3514	4.098	5.475

Pairwise Comparisons

(I) Group	(J) Group	Mean Difference (I-J)	Std. Error	df	Sig.	95% Wald Confidence ... Lower
EUT	OB	.820	.8084	1	.311	-.765
OB	EUT	-.820	.8084	1	.311	-2.404

Pairwise Comparisons

(I) Group	(J) Group	95% Wald Confidence ... Upper
EUT	OB	2.404
OB	EUT	.765

Pairwise comparisons of estimated marginal means based on the original scale of dependent variable PCI (°)

Overall Test Results

Wald Chi-Square	df	Sig.
1.028	1	.311

The Wald chi-square tests the effect of Group. This test is based on the linearly independent pairwise comparisons among the estimated marginal means.

Estimated Marginal Means 2: Speed

Estimates

Speed	Mean	Std. Error	95% Wald Confidence Interval	
			Lower	Upper
1	9.538	1.0742	7.432	11.643
2	7.237	.8003	5.669	8.806
3	3.572	.3866	2.814	4.329
4	3.318	.4062	2.522	4.114
5	2.318	.2039	1.918	2.717

Pairwise Comparisons

(I) Speed	(J) Speed	Mean Difference (I-J)	Std. Error	df	Sig.	95% Wald Confidence ...
						Lower
1	2	2.300[a]	.9638	1	.017	.412
	3	5.966[a]	1.1402	1	.000	3.731
	4	6.219[a]	1.0632	1	.000	4.135
	5	7.220[a]	1.0419	1	.000	5.178
2	1	-2.300[a]	.9638	1	.017	-4.189
	3	3.666[a]	1.0049	1	.000	1.696
	4	3.919[a]	.8412	1	.000	2.270
	5	4.919[a]	.8040	1	.000	3.344
3	1	-5.966[a]	1.1402	1	.000	-8.201
	2	-3.666[a]	1.0049	1	.000	-5.635
	4	.253	.6202	1	.683	-.962
	5	1.254[a]	.4586	1	.006	.355
4	1	-6.219[a]	1.0632	1	.000	-8.303
	2	-3.919[a]	.8412	1	.000	-5.567

Pairwise Comparisons

(I) Speed	(J) Speed	95% Wald Confidence ... Upper
1	2	4.189
	3	8.201
	4	8.303
	5	9.262
2	1	-.412
	3	5.635
	4	5.567
	5	6.495
3	1	-3.731
	2	-1.696
	4	1.469
	5	2.153
4	1	-4.135
	2	-2.270

Pairwise Comparisons

(I) Speed	(J) Speed	Mean Difference (I-J)	Std. Error	df	Sig.	95% Wald Confidence ... Lower
	3	-.253	.6202	1	.683	-1.469
	5	1.001[a]	.4182	1	.017	.181
5	1	-7.220[a]	1.0419	1	.000	-9.262
	2	-4.919[a]	.8040	1	.000	-6.495
	3	-1.254[a]	.4586	1	.006	-2.153
	4	-1.001[a]	.4182	1	.017	-1.820

Pairwise Comparisons

(I) Speed	(J) Speed	95% Wald Confidence ... Upper
	3	.962
	5	1.820
5	1	-5.178
	2	-3.344
	3	-.355
	4	-.181

Pairwise comparisons of estimated marginal means based on the original scale of dependent variable PCI (°)

a. The mean difference is significant at the .05 level.

Overall Test Results

Wald Chi-Square	df	Sig.
72.053	4	.000

The Wald chi-square tests the effect of Speed. This test is based on the linearly independent pairwise comparisons among the estimated marginal means.

```
 * Generalized Estimating Equations.
GENLIN PCI BY Group Speed (ORDER=ASCENDING) WITH CMI
  /MODEL Group Speed CMI INTERCEPT=YES
 DISTRIBUTION=NORMAL LINK=IDENTITY
  /CRITERIA SCALE=MLE PCONVERGE=1E-006(ABSOLUTE) SINGULAR=1E-012 ANALYSISTY
PE=3(WALD) CILEVEL=95
    LIKELIHOOD=FULL
  /EMMEANS TABLES=Group SCALE=ORIGINAL COMPARE=Group CONTRAST=PAIRWISE PADJ
UST=LSD
  /EMMEANS TABLES=Speed SCALE=ORIGINAL COMPARE=Speed CONTRAST=PAIRWISE PADJ
UST=LSD
  /REPEATED SUBJECT=Subject SORT=YES CORRTYPE=INDEPENDENT ADJUSTCORR=YES CO
VB=ROBUST
  /MISSING CLASSMISSING=EXCLUDE
  /PRINT CPS DESCRIPTIVES MODELINFO FIT SUMMARY SOLUTION.
```

Generalized Linear Models

Notes

Output Created		14-AUG-2023 13:25:36
Comments		
Input	Data	C:\Users\andre\Documents\Andre\Pesquisa\Artigos para Publicar\Henrique Bianchi Mestrado\Statistics\Statistics Sheets\Statistics_Sheet_withFroude.sav
	Active Dataset	DataSet1
	Filter	<none>
	Weight	<none>
	Split File	<none>
	N of Rows in Working Data File	68
Missing Value Handling	Definition of Missing	User-defined missing values for factor, subject and within-subject variables are treated as missing.
	Cases Used	Statistics are based on cases with valid data for all variables in the model.
Weight Handling		not applicable

Notes

Syntax		GENLIN PCI BY Group Speed (ORDER=ASCENDING) WITH CMI<br>/MODEL Group Speed CMI INTERCEPT=YES<br><br>DISTRIBUTION=NORMAL LINK=IDENTITY<br>/CRITERIA SCALE=MLE PCONVERGE=1E-006 (ABSOLUTE) SINGULAR=1E-012 ANALYSISTYPE=3 (WALD) CILEVEL=95<br>LIKELIHOOD=FULL<br>/EMMEANS TABLES=Group SCALE=ORIGINAL COMPARE=Group CONTRAST=PAIRWISE PADJUST=LSD<br>/EMMEANS TABLES=Speed SCALE=ORIGINAL COMPARE=Speed CONTRAST=PAIRWISE PADJUST=LSD<br>/REPEATED SUBJECT=Subject SORT=YES CORRTYPE=INDEPENDENT ADJUSTCORR=YES COVB=ROBUST<br>/MISSING CLASSMISSING=EXCLUDE<br>/PRINT CPS DESCRIPTIVES MODELINFO FIT SUMMARY SOLUTION.
Resources	Processor Time	00:00:00.05
	Elapsed Time	00:00:00.04

Model Information

Dependent Variable		PCI (°)
Probability Distribution		Normal
Link Function		Identity
Subject Effect	1	Subject
Working Correlation Matrix Structure		Independent

Case Processing Summary

	N	Percent
Included	68	100.0%
Excluded	0	0.0%
Total	68	100.0%

Correlated Data Summary

Number of Levels	Subject Effect	Subject	17
Number of Subjects			17
Number of Measurements per Subject	Minimum		2
	Maximum		5
Correlation Matrix Dimension			5

Categorical Variable Information

			N	Percent
Factor	Group	EUT	36	52.9%
		OB	32	47.1%
		Total	68	100.0%
	Speed	1	16	23.5%
		2	14	20.6%
		3	13	19.1%
		4	12	17.6%
		5	13	19.1%
		Total	68	100.0%

Continuous Variable Information

		N	Minimum	Maximum	Mean	Std. Deviation
Dependent Variable	PCI (°)	68	1.2	18.8	5.470	3.8941
Covariate	CMI	68	.620	.835	.69147	.053577

Goodness of Fit[a]

	Value
Quasi Likelihood under Independence Model Criterion (QIC)[b]	491.028
Corrected Quasi Likelihood under Independence Model Criterion (QICC)[b]	491.387

Dependent Variable: PCI (°)
Model: (Intercept), Group, Speed, CMI

a. Information criteria are in smaller-is-better form.

b. Computed using the full log quasi-likelihood function.

Tests of Model Effects

	Type III		
Source	Wald Chi-Square	df	Sig.
(Intercept)	.775	1	.379
Group	.989	1	.320
Speed	69.055	4	.000
CMI	.045	1	.831

Dependent Variable: PCI (°)
Model: (Intercept), Group, Speed, CMI

Parameter Estimates

Parameter	B	Std. Error	95% Wald Confidence Interval Lower	Upper	Hypothesis Test Wald Chi-Square	df
(Intercept)	.822	5.0758	-9.127	10.770	.026	1
[Group=1]	.927	.9325	-.900	2.755	.989	1
[Group=2]	0[a]	.	.	.	.	.
[Speed=1]	7.225	1.0488	5.169	9.280	47.447	1
[Speed=2]	4.930	.8253	3.312	6.547	35.685	1
[Speed=3]	1.260	.4506	.377	2.144	7.823	1
[Speed=4]	1.010	.4130	.200	1.820	5.980	1
[Speed=5]	0[a]	.	.	.	.	.
CMI	1.480	6.9492	-12.141	15.100	.045	1
(Scale)	7.826					

Parameter Estimates

Parameter	Hypothesis .. Sig.
(Intercept)	.871
[Group=1]	.320
[Group=2]	.
[Speed=1]	.000
[Speed=2]	.000
[Speed=3]	.005
[Speed=4]	.014
[Speed=5]	.
CMI	.831
(Scale)	

Dependent Variable: PCI (°)
Model: (Intercept), Group, Speed, CMI

a. Set to zero because this parameter is redundant.

Estimated Marginal Means 1: Group

Estimates

Group	Mean	Std. Error	95% Wald Confidence Interval Lower	Upper
EUT	5.657	.7400	4.207	7.107
OB	4.730	.3677	4.009	5.450

Covariates appearing in the model are fixed at the following values: CMI=.69147

Pairwise Comparisons

(I) Group	(J) Group	Mean Difference (I-J)	Std. Error	df	Sig.	95% Wald Confidence ... Lower
EUT	OB	.927	.9325	1	.320	-.900
OB	EUT	-.927	.9325	1	.320	-2.755

Pairwise Comparisons

(I) Group	(J) Group	95% Wald Confidence ... Upper
EUT	OB	2.755
OB	EUT	.900

Pairwise comparisons of estimated marginal means based on the original scale of dependent variable PCI (°)

Overall Test Results

Wald Chi-Square	df	Sig.
.989	1	.320

The Wald chi-square tests the effect of Group. This test is based on the linearly independent pairwise comparisons among the estimated marginal means.

Estimated Marginal Means 2: Speed

Estimates

Speed	Mean	Std. Error	95% Wald Confidence Interval	
			Lower	Upper
1	9.533	1.0680	7.440	11.626
2	7.238	.8007	5.669	8.808
3	3.569	.3899	2.805	4.333
4	3.318	.4095	2.516	4.121
5	2.308	.2042	1.908	2.709

Covariates appearing in the model are fixed at the following values: CMI=.69147

Pairwise Comparisons

(I) Speed	(J) Speed	Mean Difference (I-J)	Std. Error	df	Sig.	95% Wald Confidence ...
						Lower
1	2	2.295[a]	.9612	1	.017	.411
	3	5.964[a]	1.1378	1	.000	3.734
	4	6.214[a]	1.0563	1	.000	4.144
	5	7.225[a]	1.0488	1	.000	5.169
2	1	-2.295[a]	.9612	1	.017	-4.178
	3	3.670[a]	1.0128	1	.000	1.684
	4	3.920[a]	.8444	1	.000	2.265
	5	4.930[a]	.8253	1	.000	3.312
3	1	-5.964[a]	1.1378	1	.000	-8.194
	2	-3.670[a]	1.0128	1	.000	-5.655
	4	.250	.6218	1	.687	-.968
	5	1.260[a]	.4506	1	.005	.377
4	1	-6.214[a]	1.0563	1	.000	-8.285
	2	-3.920[a]	.8444	1	.000	-5.575
	3	-.250	.6218	1	.687	-1.469
	5	1.010[a]	.4130	1	.014	.200
5	1	-7.225[a]	1.0488	1	.000	-9.280
	2	-4.930[a]	.8253	1	.000	-6.547
	3	-1.260[a]	.4506	1	.005	-2.144
	4	-1.010[a]	.4130	1	.014	-1.820

Pairwise Comparisons

(I) Speed	(J) Speed	95% Wald Confidence ... Upper
1	2	4.178
	3	8.194
	4	8.285
	5	9.280
2	1	-.411
	3	5.655
	4	5.575
	5	6.547
3	1	-3.734
	2	-1.684
	4	1.469
	5	2.144
4	1	-4.144
	2	-2.265
	3	.968
	5	1.820
5	1	-5.169
	2	-3.312
	3	-.377
	4	-.200

Pairwise comparisons of estimated marginal means based on the original scale of dependent variable PCI (°)

a. The mean difference is significant at the .05 level.

Overall Test Results

Wald Chi-Square	df	Sig.
69.055	4	.000

The Wald chi-square tests the effect of Speed. This test is based on the linearly independent pairwise comparisons among the estimated marginal means.
